# Abstracts from the 26th European Society for Animal Cell Technology Meeting - Cell culture technologies: bridging academia and industry to provide solutions for patients: Copenhagen, Denmark. 05-08 May 2019

**DOI:** 10.1186/s12919-020-00188-y

**Published:** 2020-05-25

## ESACT 2019 - Publication of conference proceedings

### O-009 Overcoming cellular heterogeneity during cell line development

#### Leon P Pybus, Ellie Hawke, Christopher Knowles, Devika Kalsi, Nicholas Barber, Alison Young, Fay L Saunders

##### FUJIFILM Diosynth Biotechnologies, Mammalian Cell Culture Process Development, Billingham, TS23 1LH

###### **Correspondence:** Leon P Pybus (leon.pybus@fujiflm.com)

**Background**

The generation of a recombinant cell line for biomanufacturing purposes lies on the critical path to an investigational new drug (IND) submission. Therefore, acceleration of this timeline has a direct impact on the delivery of high quality, innovative medicines to patients.

A balance must be struck between speed and quality to reach quick and efficient IND approval. For example, the goal of a fast development process with minimal cell screening must be balanced with requirements including high titre, favourable product quality attributes and the maintenance of regulatory compliance.

Cell line development is traditionally a lengthy process and it is common to find development timelines exceeding 6 months. Limitations include cellular heterogeneity and the regulatory requirement for high probability and assurance of monoclonality which may require rounds of single cell cloning.

In this study we explore approaches to mitigate clonal variation and develop a next generation expression system capable of maintaining quality in an accelerated time frame.

**Materials and methods**

*Directed Evolution* – CHO-DG44 host cell lines were cultured in 2L continuous chemostat culture [1] for 51 days. Host cells were then cultured on a reduced subculture regime for 40 days.

*Fed-batch process* – Recombinant CHO-DG44 cell lines expressing one of four recombinant monoclonal antibodies (mAbs) underwent a 14 day fed-batch process in an ambr® 15 (Sartorius)

**Results**

Firstly, we utilised a directed evolution [2] approach to improve the properties of our host cell line. A number of directed evolution strategies were trialled and the resulting host cell line were compared for their ability to express different mAbs. A ~2-fold improvement in fed-batch titre (Figure 1A) was obtained by utilising a host cell line that underwent directed evolution.

Next, we combined the single cell deposition, imaging and productivity screening capability of Sphere Fluidics’ Cyto-Mine® technology [3] with the plate imaging capability of the Solentim CellMetric®. This created a novel workflow for the generation of high quality clonal cell lines with both high probability (>99%) and assurance of monoclonality in a single round of cloning with a 10-week cell line development timeline (Transfection to Research Cell Bank generation; Figure 1B).

An optimised chemically defined and protein free basal medium was also developed. On average cell line titre increased by 20% and mAb product quality was comparable. Several cell lines with high titres of 11 g/L (Figure 1C) and favourable product quality attributed (data not shown) were obtained which allows more choice for selecting the correct cell line to progress to GMP manufacture. Cell line stability was assessed over 60 generations and > 90% of cell lines maintained production titres (data not shown). Furthermore, all cell lines produced mAb with consistent product quality attributes.

**Conclusion**

Fast tracking cell line development whilst maintaining quality involved moving beyond the modulation of individual expression system components towards a more holistic strategy to maximise cell line development output.

For the host cell line we utilised a directed evolution strategy to exploit intrinsic host cell line heterogeneity and identify those with improved biomanufacturing attributes.

The introduction of new microfluidic technology (Cyto-Mine®) enables the screening of large numbers of cell lines early in development using a predictive productivity assay. High assurance and probability of monoclonality (>99%) can also be achieved by combining the Cyto-Mine® and Cell Metric®.

Furthermore, a tailor-made basal media supported high fed-batch titres (> 10 g/L) for several cell lines at the end of a 10-week cell line development timeline (Transfection to Research Cell Bank generation).

**Acknowledgements**

Mammalian Cell Culture Process Development (FUJIFILM Diosynth Biotechnologies, U.K.), Analytical Development (FUJIFILM Diosynth Biotechnologies, U.K.), Bioscience and Engineering Laboratory (FUJIFILM Corp., Japan) and Sphere Fluidics (Cambridge, U.K.).

**References**

1. Adamberg K., Valgepea K., Vilu R. Advanced cultivation methods for systems microbiology. Microbiology; 161: 1707-1719.

2. Majors B.S., Chiang G.G., Betenbaugh M.J. Protein and genome evolution in mammalian cells for biotechnology applications. Mol Biotechnol; 42: 216-223.

3. Kelly T., Tuckowski A.M., Smith K.D. Rapid generation of high-producing clonal cell lines: Using FRET-based microfluidic screening for analysis, sorting, imaging, and dispensing. Bioprocess Int. 2018; 16:19-24.

**Fig. 1 (abstract O-009).**
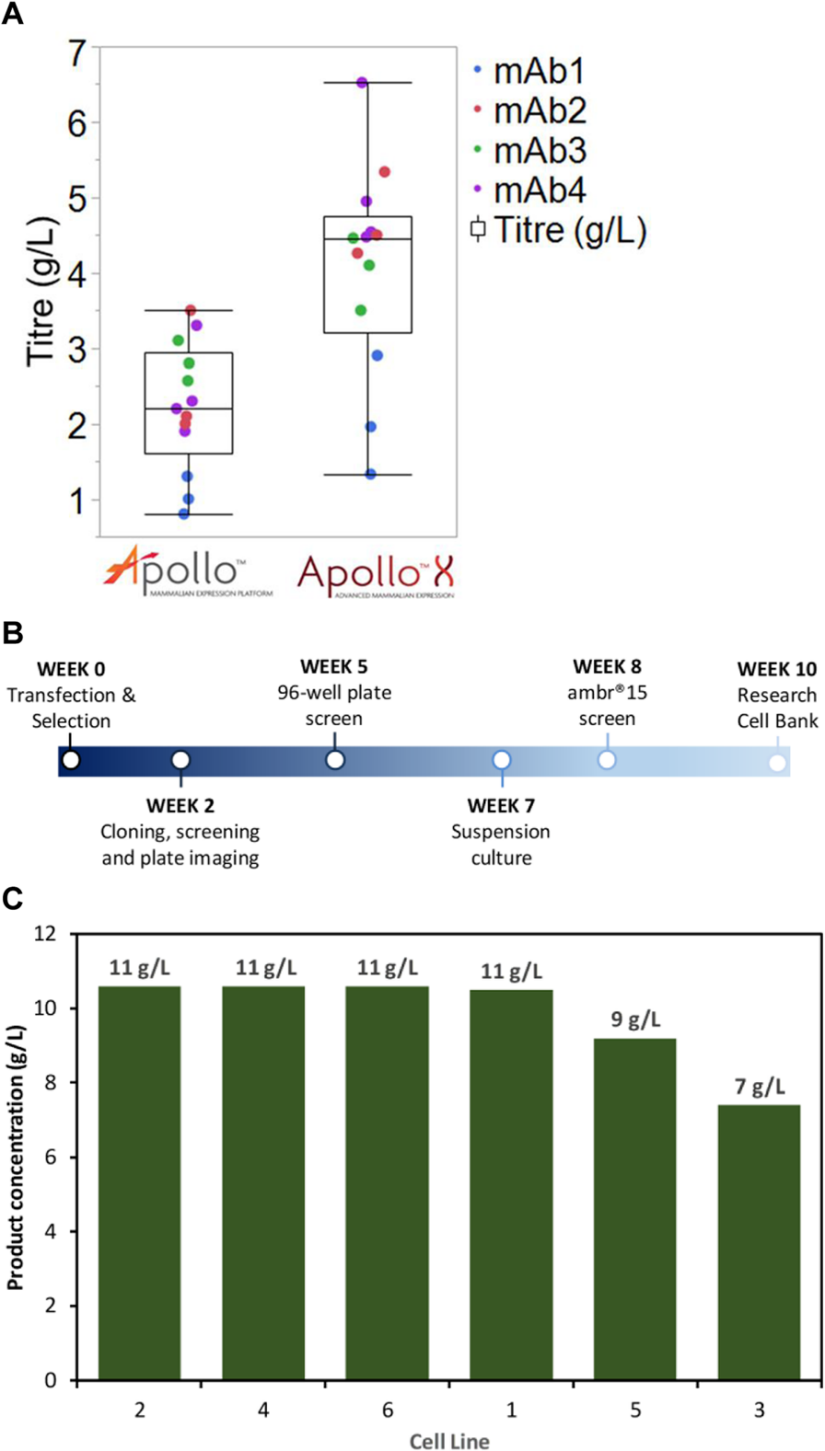
A multifaceted approach to accelerate cell line development whilst maintaining quality. (A) Protein A HPLC quantified day 14 fed-batch titres for recombinant cell lines derived from Apollo™ (limiting dilution cloning) and Apollo™ X (Chemostat) host cell lines. Four mAbs were expressed in each cell line. (B) Timeline showing transfection to research cell bank in 10 weeks, (C) Protein A HPLC quantified day 14 fed-batch titres for six recombinant DG44 cell lines expressing the same mAb

### O-028 Customized process models for cell culture processes

#### Harini Narayanan^1^, Michael Sokolov^1,2^, Alessandro Butte^1,2^, Massimo Morbidelli^1,2^

##### ^1^Institute of Chemical and Bioengineering, ETH Zurich, Switzerland; ^2^DataHow AG, Zurich, Switzerland

###### **Correspondence:** Harini Narayanan (nharini@chem.ethz.ch)

**Background**

The need for rapid and efficient process development to meet market demands, calls for the use of model-based methods to assist at various stages of development, monitoring, and control of bioprocesses. Thus, efforts have been made to develop models from historical and online spectral and sensor data.

A cell culture process undergoes a life cycle from screening where different experimental conditions are tested. Then to a development phase where the process is optimized to produce the product of certain quantity and quality. These are then validated and transferred to the production phase. Models can be of assistance at each of these phases [1] but the characteristic of the process model required is remarkably different. The aim of this work is to highlight the need for process models that are customized based on application. We demonstrate through exemplary cases, tailored toolkits for use at different stages during the cell culture process.

**Materials and methods**

Three different datasets were used in this work to illustrate the concept of customized process models. The first dataset is from a yeast culture [2], the second from a mammalian cell culture at pilot bioreactor scale (3.5 L) [3] and the third from a mammalian cell culture at AmBR 15 mL scale [4]. The detailed description of the experimental procedure and data collected is documented in the literature cited.

Process models were developed using different machine learning tools such as Principal Component Analysis (PCA)[5], Partial Least Squares (PLS) [6], Decision trees (DTs) [7] and Artificial Neural Networks which were used solely, in combination or in synergy with the mechanistic framework for instance mass balances (known as Hybrid models [8]).

**Results**

**Case1: Screening experiments**

Parallel experiments performed during screening can be monitored, replicates can be tracked and sensor failures can be detected using simple unsupervised tools such as PCA.

We showed this for yeast cell culture screening experiments that were done in parallel bioreactors systems with robotic liquid handler in [2].

**Case2: Screening to process development**

During screening, a wide range of process conditions is tested. Algorithms are needed that can automatically segregate experiments into process relevant clusters while explaining the influence of different designed factors on the final target. We developed a DT-PLS algorithm capable of doing this. Using AmBR dataset (at 15 mL scale) we demonstrated in [9] that, DT-PLS algorithm not only reduces the design space but also presents a better picture of the different influencing factor on titer and quality attribute.

**Case3: Model-based process optimization**

After having done the screening studies and identifying the relevant region in design space, it is of interest to optimize the process to get maximal benefits. This can be done *in-silico*, thus, immensely reducing experimental cost and effort. However this requires models that are robust and extrapolatable. For this application hybrid model performs best as illustrated in [10] on a pilot scale mammalian cell culture.

**Case4: Process Control**

Once the process is optimized and put to production, it is important to control the process in an adaptive manner to ensure consistent quantity and quality of product. The hybrid model in combination with filtering approaches are then suitable for making control decisions.

**Conclusions**

We have highlighted that based on application, the characteristic of process models required is very different. The role of model could be descriptive and diagnostic (case1) or diagnostic and predictive (case2) or predictive and prescriptive (case3 and case4). Thus the selection of model is highly need specific and customized toolkits must be developed. Through our previous work we have developed tools on various cell culture processes at different scales. In this work we highlight the customization of these tools in the different phases of cell culture processes.

**References**

1. Narayanan H, Luna MF, von Stosch M, Cruz Bournazou MN, Polotti G, Morbidelli M, et al. Bioprocessing in the digital age – the role of process models. Biotechnol J 2019. doi:DOI: 10.1002/biot.201900172.

2. Sawatzki A, Hans S, Narayanan H, Haby B, Krausch N, Sokolov M, et al. Accelerated Bioprocess Development of Endopolygalacturonase-Production with Saccharomyces cerevisiae Using Multivariate Prediction in a 48 Mini-Bioreactor Automated Platform. Bioengineering 2018;5:101. doi:10.3390/bioengineering5040101.

3. Rouiller Y, Solacroup T, Deparis V, Barbafieri M, Gleixner R, Broly H, et al. Application of Quality by Design to the characterization of the cell culture process of an Fc-Fusion protein. Eur J Pharm Biopharm 2012;81:426–37. doi:10.1016/j.ejpb.2012.02.018.

4. Sokolov M, Ritscher J, MacKinnon N, Souquet J, Broly H, Morbidelli M, et al. Enhanced process understanding and multivariate prediction of the relationship between cell culture process and monoclonal antibody quality. Biotechnol Prog 2017;33:1368–80. doi:10.1002/btpr.2502.

5. Abdi H, Williams LJ. Principal component analysis. Wiley Interdiscip Rev Comput Stat 2010;2:433–59. doi:10.1002/wics.101.

6. Wold S, Sjöström M, Eriksson L. PLS-regression: A basic tool of chemometrics. Chemom Intell Lab Syst 2001;58:109–30. doi:10.1016/S0169-7439(01)00155-1.

7. Breiman L, Friedman J, Stone CJ, Olshen RA. Classification and regression trees 1984;1:368. doi:10.1371/journal.pone.0015807.

8. Thompson ML, Kramer MA. Modeling chemical processes using prior knowledge and neural networks. AIChE J 1994;40:1328–40. doi:10.1002/aic.690400806.

9. Narayanan H, Sokolov M, Butté A, Morbidelli M. Decision Tree – PLS (DT ‐ PLS) algorithm for the development of process ‐ specific local prediction models. Biotechnol Prog 2019:e2818. doi:10.1002/btpr.2818.

10. Narayanan H, Sokolov M, Morbidelli M, Butté A. A new generation of predictive models–the added value of hybrid models for manufacturing processes of therapeutic proteins. Biotechnol Bioeng 2019.

**Fig. 1 (abstract O-028).**
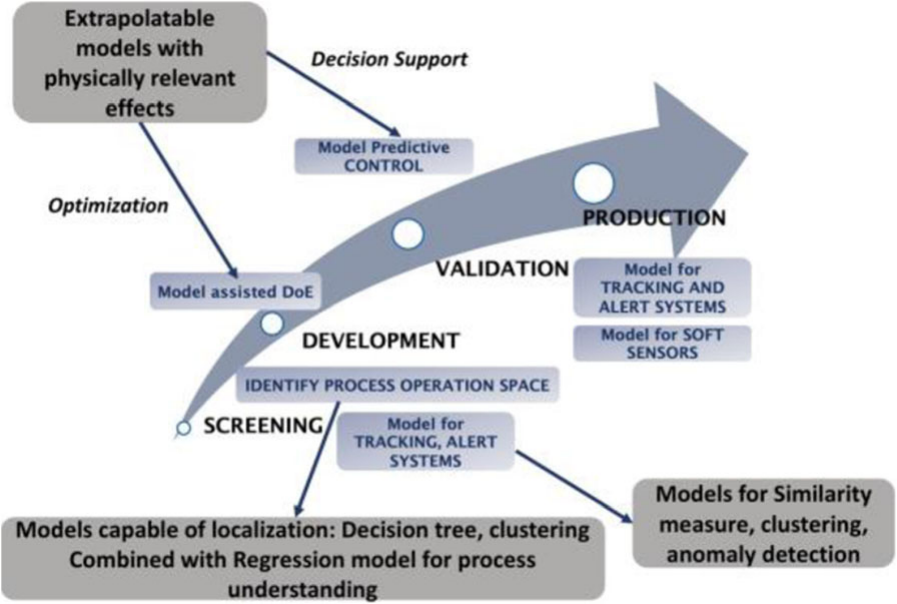
Schematics of the different phases of cell culture process with roles of models highlighted. Additionally the plausible characteristic to be possessed by models is presented

### P-103 Manipulation of organelle content in CHO Cells for increased recombinant protein production

#### Eva Pekle^1,2^, Guglielmo Rosignoli^3^ Andrew Smith^1^, Luigi Grassi^1^, Chris Sellick^1^, Claire Pearce^1^, Claire Harris^1^ and Mark Smales^2^

##### ^1^Cell Culture and Fermentation Sciences, Biopharmaceutical Development, BioPharmaceuticals R&D, AstraZeneca, Granta Park, Cambridge, CB21 6GH, UK; ^2^School of Biosciences, University of Kent, Canterbury, Kent, CT2 7NJ, UK; ^3^ADPE, AstraZeneca, Granta Park, Cambridge, CB21 6GH, UK

###### **Correspondence:** Eva Pekle (c.m.smales@kent.ac.uk)

**Background**

The aim of biopharmaceutical development is to produce recombinant proteins with the desired product quality at commercial quantities. Regulatory agencies require production cell lines to originate from a single cell. Different methods can be used to achieve clonality, such as single cell sorting via flow cytometry (FACS). Following cloning, hundreds of clones are screened to identify high producers. Enriching cells with high expression levels of the recombinant protein during the cloning process could reduce the number of clones screened and development timelines. Previous work has investigated the organelle content of a panel of cell lines with differing expression levels of a model antibody [1]. In this work lysosome content has been identified to correlate with productivity, therefore increasing lysosome content was attempted in two ways: 1) by using FACS to sort cells based on their lysosome content and 2) by chemically evolving the host cell line using a lysosomotropic agent (referred to as L).

**Materials and methods**

A mix of clonal cell lines expressing a model antibody with different productivities was sorted by FACS to generate pools based on their lysosome content (high, mid and low). Cells were assessed for productivity in a fed-batch overgrow (FBOG). For the directed evolution, the host cell line was treated with L until the treatment no longer affected its viability or growth. The L-evolved and standard host cell lines were used to generate pools producing an easy-to-express (ETE) or a difficult-to-express (DTE) mAb. Following transfection, treatment with L was stopped and cell lines were assessed in a FBOG. Transcriptomic analysis by RNA-sequencing was then undertaken to investigate any differences between the two hosts.

**Results**

Cell sorting based on high lysosome content resulted in increased titre and overall qP compared to the mid and low lysosome content sorted cells. This improvement was also seen compared to the original cell lines that were mixed prior to sorting except for one cell line (Fig. 1A). Moreover, despite similar lysosome content earlier in the culture of a FBOG, by day 10, the high lysosome content sorted cells showed an increased lysosomal capacity (Fig. 1B). Interestingly, the high lysosome content pools also had a more homogeneous HC and LC expressing population, explaining the increase in titre (Fig.1C).

For the directed evolution of the host cell line using L, the evolved host showed a 2-fold increase in productivity (both for final titre and overall qP), as well as maintaining a higher culture viability at later stages of the FBOG. Transcriptomic analysis was performed to investigate the underlying mechanisms for the increased productivity in the evolved host. This revealed an overall down-regulation of genes in the evolved host, with only a few genes up-regulated. Further analysis of transcriptomic data showed that a small number of genes were differentially expressed, which did not include any lysosomal genes. Genes related to glutathione metabolism and oxidative stress were up-regulated in the evolved host, consistent with other studies that show increased glutathione synthesis and oxidative metabolism in high titre cell lines [2, 3].

**Conclusions**

Manipulation of lysosome content, either by screening using flow cytometry or by directed evolution, has been demonstrated as a potential strategy for increasing CHO cell productivity.

**Acknowledgements**

The authors would like to thank Charlotte Godfrey, Sarah Dunn, Diane Hatton and Maiken Kristiansen

**References**

1. Pekle E, Smith A, Rosignoli G, Sellick C, Smales C.M, Peace C, Application of imaging flow cytometry for the characterization of intracellular attributes in Chinese hamster ovary cell lines at the single cell level, Biotechnol. J. 2019; 14.

2. Orellana C.A, Marcellin E, Schulz B.L, Nouwens A.S, Nielsen L.K, High-antibody-producing Chinese hamster ovary cells up-regulate intracellular protein transport and glutathione synthesis. J. Proteome Res. 2015; 14(2):609-618.

3. Templeton N, Dean J, Reddy P, Young JD, Peak antibody production is associated with increased oxidative metabolism in an industrially relevant fed-batch CHO cell culture. Biotechnol Bioeng. 2013; 110(7):2013-2014.

**Fig. 1 (abstract P-103).**
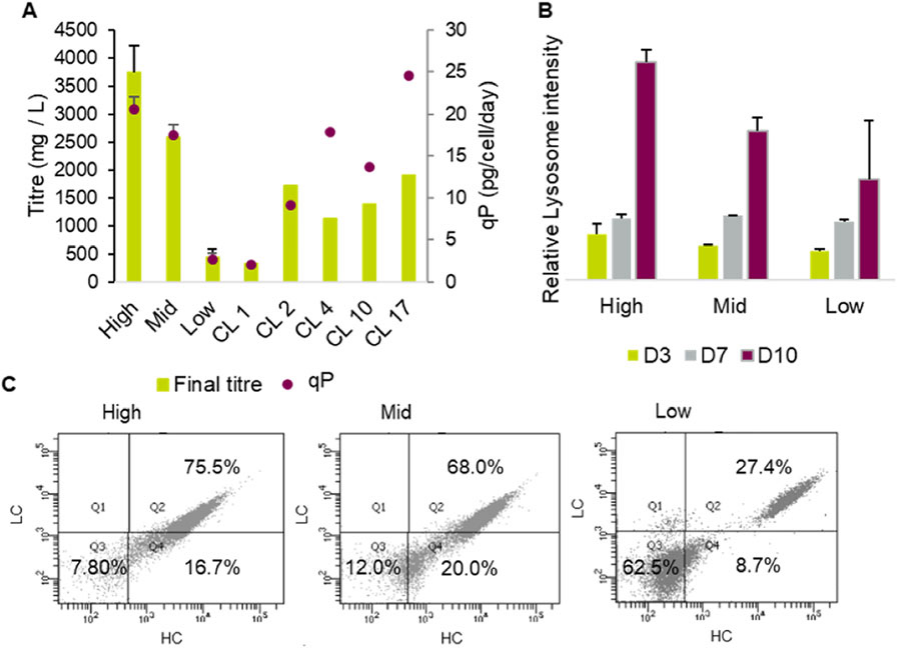
A) Titre and qP results from the High, Mid, Low sorted cells, and the original cell lines (CL1-17). B) Lysosome content throughout the FBOG. C) Intracellular HC and LC content

### P-112 Using CRISPR/Cas9 to increase HEK293 capabilities for bioprocess industry

#### Ramon Román^1^, Joan Miret^2^, Antoni Casablancas^1^, Martí Lecina^2^, Jordi J. Cairó^2^

##### ^1^Fermentation Pilot Plant, UAB, Barcelona, Spain; ^2^Chemical, Biological and Enviromental Engineering department, UAB, Barcelona, Spain

###### **Correspondence:** Ramon Román (ramon.roman@uab.cat)

**Background**

Genomic editing through CRISPR systems has pushed forward the research in all cell biology related fields. CRISPR can be applied for improving producer cell lines and expand its capabilities beyond the wild-type cell, what is of great interest for the biopharmaceutical manufacturing industry. This feature could become very interesting in cell lines like HEK293, a human cell line with several advantages for biologics production, like human glycosylation patterns in protein products, but less used than other cell lines, such as CHO. Although, HEK293 cells have been less explored and offer lower product titers, the interest in improving HEK293-based platforms for protein production has increased^1,2^.

In the present work, CRISPR was used to target a gene of interest, human interferon gamma, into a previously defined chromosome location using Micro-Homologous End Joining (MHEJ)^3^ in order to improve genomic context and raise up product titers in HEK293 cells.

**Material and Methods**

Cell lines were cultivated in shake flasks using standard conditions in SFM4Transfex media (Hyclone) supplemented with 6% (w/v) CellBoost 5 (Hyclone). Viable cell density and viability were measured using Neubauer hemocytometer and Trypan blue staining. Transfection efficiency was determined via detection of GFP expressing cells in flow cytometry 24 h post-transfection. hIFNγ yields were determined by ELISA (ThermoFisher) in culture broth supernatants. Gene copy numbers were determined by qPCR using linearized plasmid as copy number standard.

Cells were transfected at densities of 1∙10^6^ cells/mL in SFM4Transfex media with K2 Transfection kit (Biontex, Germany) and 1 mg/mL of total plasmid DNA. Clonal isolation was performed by single cell seeding in 96 well plates using Becton Dickinson FACSJazz cell sorter.

**Results**

Firstly, an eGFP expressing cell pool was generated by transfection of pIRES_eGFP/Neo expression vector. After appropriate G418 selection, the cell pool was subjected to cell sorting looking for the cells with higher fluorescence level. Several clones were selected and the eGFP copy number was determined by qPCR. N1D8 clone was selected as its present a single eGFP gene located in 6q24.3. (Fig 1A)

Secondly, a Bicistronic expression vector containing hIFNγ/Puro and Micro-homology arms (MHA) and CRISPR containing plasmid were co-transfected into the N1D8 cell line. (Fig 1B). sgRNA was designed to knock-in the transgene into the eGFP locus. Productive clones were then selected using cell sorting by pooling singles cells from non-fluorescent population. Correct insertion at desired location was checked by inverse PCR plus Sanger DNA sequencing (Fig 1C).

hIFNγ copy number in transfected clones was determined by qPCR. Most of screened clones showed single or tandem integration in the desired location, whereas the control clones, transfected only with the transgene vector without the CRISPR plasmid shows higher heterogeneity in both copy number and integration site, as expected in a not targeted, random integration. Besides, mean hIFNγ protein titters were about 5 times higher in the cell line obtained using targeted integration than in control cell line obtained by random transfection (Table 1)^4^.

**Conclusions**

In this work, we developed a CRISPR/cas9-based technique to target hIFNγ gene into a selected, advantageous chromosomal location HEK293 cell lines. This location was previously determined experimentally to be a transcriptional hotspot using eGFP^5^. This technique allows a quick transfection and selection workflow with predictable results and improved product titters, with a five-fold increase in hIFNγ production.

Thus, the obtained results show the usefulness of gene editing techniques, such as CRISPR, in order to overcome HEK293 limitations for stable cell line generation towards biotherapeutic production.

**Acknowledgements**

The authors would like to thank Dr. A. Kamen (National Research Council of Canada) for kindly providing the HEK293 cells, which this work was performed with.

**References**

1. Liste-Calleja et al. Lactate and glucose concomitant consumption as a self-regulated pH detoxification mechanism in HEK293 cell cultures. *Appl. Microbiol. Biotechnol.*, (2015) 99(23):9951-9960.

2. Martínez‐Monge et al. Metabolic flux balance analysis during lactate and glucose concomitant consumption in HEK293 cell cultures. *Biotechnol Bioeng* (2019) *116*(2): 388-404.

3. Aida, Tomomi, et al. “Gene cassette knock-in in mammalian cells and zygotes by enhanced MMEJ.” *BMC genomics (2016)* 17.1: 979.

4. Román, R., et al. Enhancing heterologous protein expression and secretion in HEK293 cells by means of combination of CMV promoter and IFNα2 signal peptide. *J.Biotechnol. (2016)*, *239*: 57-60.

5. Román, R., et al. Enabling HEK293 cells for antibiotic-free media bioprocessing through CRISPR/Cas9 gene editing. *Biochem. Eng. J. (2019) 151:*107299.

**Table 1 (abstract P-112).** Transgene copy number and hIFNγ titter of clones isolated after transfection using either targeted or random integration

Method	Clone	Copy number	Titer (mg/L)
Targeted integration	**1**	1,4±0,2	10,08
	**2**	2,8±0,1	8,42
	**3**	1,2±0,1	7,24
	**4**	2,4±0,3	6,96
	**5**	1,6±0,1	6,81
	**6**	2,3±0’2	6,51
	**7**	1,7±0,2	6,45
Random integration	**1**	5,9±0,2	2,44
	**2**	4,1±0,1	1,74
	**3**	1,3±0,1	1,69
	**4**	6,4±0,1	1,65
	**5**	3,2±0,4	1,64
	**6**	5,6±0,1	1,49
	**7**	5,9±0,2	1,41

**Fig. 1 (abstract P-112).**
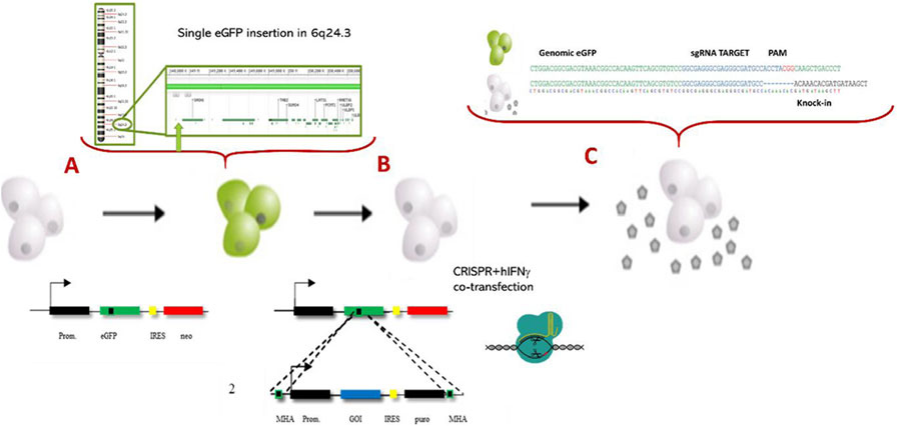
CRISPR-based targeted integration of hIFNγ transgene into HEK293 cells. HEK293 N1D8 cell line with single eGFP insertion into a chromosomal hotpost (A) was transfected with hIFNγ expression vector with 20bp homology arms together with a CRISPR expression vector containing anti-eGFP sgRNA (B). Clones were isolated and correct knock-in of hIFNγ gene in the desired spot was checked (C)

### P-115 K-D-E-L receptor 1 gene dynamics and over-expression in recombinant CHO cells

#### Andrew Samy, Kohei Kaneyoshi, Takeshi Omasa

##### Division of Advanced Science and Biotechnology, Graduate School of Engineering, Osaka University, Osaka, Japan

###### **Correspondence:** Andrew Samy (omasa@bio.eng.osaka-u.ac.jp)

**Background**

Overexpression of endoplasmic reticulum (ER) chaperones is a popular cell engineering approach to enhance the folding and assembly of recombinant proteins in Chinese hamster ovary (CHO) cells [1]. However, whether recombinant CHO cells are capable of efficiently retaining ER chaperones during either exogenous overexpression or during natural upregulation under unfolded protein response has not yet been investigated. Overexpressing transcription factors that enhance chaperone upregulation is another common method for improving productivity [2,3]. These soluble ER chaperones have a C-terminal KDEL (Lys–Asp–Glu–Leu) motif that ensures their retrieval from post ER-compartments. This motif is recognized by KDEL endoplasmic reticulum protein retention receptors, proteins with seven transmembrane domains that cycle between the ER and *cis*-Golgi to retain mis-sorted ER chaperones [4,5]. In this study, we aimed to investigate this ER retention system in terms of KDEL protein sequence homology and localization in CHO cells.

**Materials and methods**

To investigate the localization of KDELR1 in CHO cells, KDELR1 was fused to citrine, a variant of green fluorescent protein. Total RNA was extracted from CHO-K1 cells followed by first strand cDNA synthesis. Then, the open reading frame (ORF) of the *Kdelr1* gene was PCR amplified using forward and reverse primers containing *Xho*I and *Pos*it restriction sites, respectively. The reverse primer had no stop codon. Then, the DNA fragment containing *Kdelr1* was inserted in frame with DNA encoding citrine in the pcDNA3.1 backbone to encode a KDELR1–citrine fusion protein. The plasmid was endotoxin purified and transfected into CHO-K1 cells. Cells were selected using 1,100 μg/mL G418 then maintained using 800 μg/mL G418.

CHO-K1-KDELR1-citrine cells were cultured on L-lysine coated coverslips in Iscove's modified Dulbecco’s medium containing 10% fetal bovine serum. Two days later they were washed three times using phosphate-buffered saline (PBS) and fixed with 4% paraformaldehyde solution. Permeabilization was performed using 0.1% Triton X-100/PBS for 5 min.

Cells were immunostained against GM130 as a *cis*-Golgi marker. Anti GFP mAb was used to stain KDELR1-citrine fusion protein, Proper secondary antibodies were chosen accordingly. ER-ID® dye was used as an ER marker. DAPI (4′,6-diamidino-2-phenylindole) was used to stain the nucleus.

**Results**

The mRNA sequence encoding the ORF of the cloned *Kdelr1* gene was sequenced and showed full identity with the predicted transcript (Gene ID: 100750912, NCBI database). The sequenced gene was submitted to the DNA Data Bank of Japan with accession number LC342268.

The KDEL receptor family in CHO-K1 cells contains three members (KDELR1, KDELR2 and KDELR3). They show high level homology, as summarized in Table 1 and Figure1a.

Immunofluorescence staining of adherent CHO-K1 cells overexpressing KDELR1–citrine fusion protein showed the expected localization in the ER and *cis*-Golgi. In Figure 1b, pseudo-coloring shows the nucleus in blue, ER or GM130 in red, and KDELR1 in green.

**Conclusions**

In this study, we investigated the ER retention machinery in CHO cells. KDELR1 was chosen as a representative of the KDEL receptor family. A KDELR1–citrine fusion protein was found to localize to both the ER and the *cis*-Golgi. This conforms with previous reports of similar localizations in other organisms [4,6]. The future aim of this research is to study the ER retention machinery as well as KDEL-bearing chaperones in CHO cells in terms of dynamics of gene expression under ER stress. The effect of KDELR1 overexpression on recombinant protein productivity will also be investigated.

**Acknowledgements**

This research was partially supported by Developing Key Technologies for Discovering and Manufacturing Pharmaceuticals Used for Next-generation Treatments (Japan Agency for Medical Research and Development (AMED) under grant numbers JP17ae0101003, JP18ae0101056,57,58, and 66) and MEXT/JSPS KAKENHI Grant Number JP17H06157. We thank Edanz Group (www.edanzediting.com/ac) for editing a draft of this manuscript.

**References**

1. Mohan C., Park S. H., Chung J. Y., and Lee G. M., “Effect of doxycycline-regulated protein disulfide isomerase expression on the specific productivity of recombinant CHO cells: thrombopoietin and antibody,” *Biotechnol. Bioeng.*, 2007; 98: 611–615.

2. Haredy A. M., Nishizawa A., Honda K., Ohya T., Ohtake H., and Omasa T., “Improved antibody production in Chinese hamster ovary cells by ATF4 overexpression.,” *Cytotechnol.*, 2013; 65: 993–1002.

3. Ohya T. Hayashi T., Kiyama E., Nishii H., Miki H., Kobayashi K., Honda K., Omasa T., and Ohtake H., “Improved production of recombinant human antithrombin III in Chinese hamster ovary cells by ATF4 overexpression,” *Biotechnol. Bioeng.*, 2008; 100: 317–324.

4. Capitani M. and Sallese M., “The KDEL receptor: new functions for an old protein,” *FEBS Lett.*, 2009; 583: 3863–3871.

5. Munro S. and Pelham H. R., “A C-terminal signal prevents secretion of luminal ER proteins,” *Cell*, 1987; 48: 899–907.

6. Dean N. and Pelham H. R., “Recycling of proteins from the Golgi compartment to the ER in yeast,” *J. Cell Biol.*, 1990; 111: 369–377.

**Table 1 (abstract P-115).** Amino acid sequence identities of KDEL receptor family members in Chinese Hamster (*Cricetulus griseus*)

	**KDELR1**	**KDELR2**	**KDELR3**
**KDELR1**		83%	73%
**KDELR2**	83%		76%
**KDELR3**	73%	76%	

**Fig. 1 (abstract P-115).**
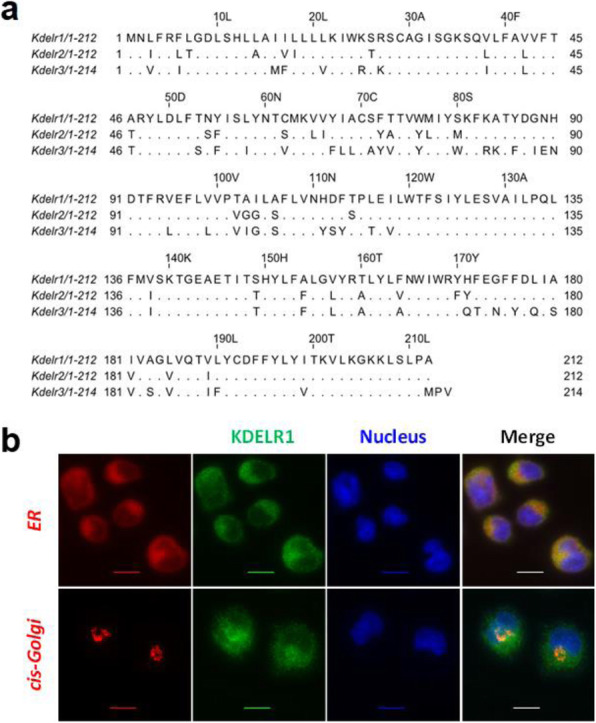
(a) Sequence alignment of the three members of the KDEL receptor family in Chinese hamster (Cricetulus griseus). (b) Localization of KDELR1–citrine fusion protein in the endoplasmic reticulum and cis-Golgi of Chinese hamster ovary cells (scale bar = 10 μm)

### P-128 Model-driven optimization of a CHO cell genome-scale metabolic model design

#### Athanasios Antonakoudis^1^, Alexandros Kiparissides^2^, Cleo Kontoravdi^1^

##### ^1^Department of Chemical Engineering, Imperial College London, London, SW7 2AZ, United Kingdom; ^2^Department of Biochemical Engineering, University College London, London, SW7 2AZ, United Kingdom

###### **Correspondence:** Athanasios Antonakoudis (cleo.kontoravdi98@imperial.ac.uk)

**Background**

Chinese hamster ovary (CHO) cells are the dominant expression platform for the production of monoclonal antibodies (mAbs), which are mainly used in the treatment of cancer and autoimmune diseases. Process optimisation of mammalian cell based manufacturing is far from trivial due to the complexity of the underlying reaction network. Therefore, model based approaches could offer significant insight on the cells’ response to changes in the culture environment. In this study, the latest consensus CHO genome-scale metabolic model, iCHO1766^1^, containing 1766 genes, 4456 metabolites and 6663 reactions was further curated by removing blocked reactions, the genes associated with them and dead-end metabolites. Subsequently, intracellular reaction fluxes were consistently constrained using carbon constrained flux balance analysis (ccFBA)^2^ in order to increase the model’s accuracy and predictive capabilities. Finally, the accuracy of gene-protein-reaction (GPR) rules was validated through single and pairwise gene essentiality studies.

**Materials and methods**

Uptake and secretion rates from a fed-batch culture conducted by Sou^3^ were used as lower and upper bound for the extracellular exchange reactions of the curated iCHO1766 model. Flux variability analysis was applied to identify and subsequently remove the reactions that could carry no flux (blocked reactions) under the studied physiology. The algorithm ccFBA was further extended to incorporate nitrogen based constrained further improving the accuracy of predicted intracellular fluxes Gene essentiality studies were performed *in silico* using the GPR rules present in the model. Each intracellular enzyme catalysed reaction is associated with its respective genes through a Boolean expression. Therefore, a (single or multiple) gene knockout can be simulated *in silico* by setting the corresponding Boolean expressions equal to 0, therefore allowing no flux through the affected reactions. Essential Genes for the growth of the cell were identified by iteratively knocking out each of the genes in a single wise and pairwise matter and then checking whether the growth rate is at least 10% of the maximum ^4^ but with a fixed production of the mAb.

**Result**

A total of 1400 reactions, 1600 metabolites and 190 genes were removed from the iCHO1766 model during the curation process described above. Experimental data was used to constrain the model to realistic physiological conditions and ccFBA augmented with additional nitrogen-based constraints was used to further refine the solution space. This led to tighter bounds applied to 60% of the model’s reactions, significantly reducing the solution space of flux balance analysis and resulting in more realistic fluxes as shown in figure 1. Importantly, elementary bounds constrained the biomass to a value much closer to the experimentally observed value: 0.0206 h^-1^ (instead of 0.0315 h^-1^) compared to the experimental growth rate which was 0.017h^-1^. Single gene knock-out simulations identified 126 genes essential for growth, out of which 51 were validated against literature data^4-7^. The rest were considered essential due to their central role in cellular metabolism as seen in table 1. Finally, the pairwise gene essentiality studies identified 25 different combinations of 2 gene-knockouts which could lead to the cell death.

**Conclusions**

In our study, we curated the iCHO1766 model, and applied experimental bounds to the uptake and secretion rates. With the use of carbon and nitrogen balance throughout the cell we constrained the allowable flux of each reaction to a realistic value, decreasing the effect of futile cycles. Lastly, the single and pairwise gene essentiality studies verified the fidelity of the GPR rules and also the most important parts of the metabolism for the CHO cell survival.

**Acknowledgments**

Funding from the UK Engineering & Physical Research Council (EPSRC) from the centre for Doctoral Training in Emergent Macromolecular Therapies hosted at University College London (Grant reference: EP/L015218/1.)

**References**

1. Hefzi, H. et al. A Consensus Genome-scale Reconstruction of Chinese Hamster Ovary Cell Metabolism. Cell Systems 3, 434-443.e438 (2016).

2. Lularevic, M., Racher, A.J., Jaques, C. & Kiparissides, A. Improving the accuracy of flux balance analysis through the implementation of carbon availability constraints for intracellular reactions. Biotechnol Bioeng 116, 2339-2352 (2019).

3. Sou, S.N. et al. How does mild hypothermia affect monoclonal antibody glycosylation? Biotechnol Bioeng 112, 1165-1176 (2015).

4. Chowdhury, R., Chowdhury, A. & Maranas, C.D. Using Gene Essentiality and Synthetic Lethality Information to Correct Yeast and CHO Cell Genome-Scale Models. Metabolites 5, 536-570 (2015).

5. Gatto, F., Miess, H., Schulze, A. & Nielsen, J. Flux balance analysis predicts essential genes in clear cell renal cell carcinoma metabolism. Sci Rep 5, 10738 (2015).

6. Kabir, M., Barradas, A., Tzotzos, G.T., Hentges, K.E. & Doig, A.J. Properties of genes essential for mouse development. PLoS One 12, e0178273 (2017).

7. Tian, D. et al. Identifying mouse developmental essential genes using machine learning. Dis Model Mech 11 (2018).

**Table 1 (abstract P-128).** Essential genes and their main role in the metabolism of the CHO cell

Number of genes	Part of the metabolism
33	Protein Synthesis
48	Oxidative Phosphorylation
14	Purine and Pyrimidine Metabolism
17	Lipid and Cholesterol Metabolism
4	Steroid Metabolism
10	Amino Acid Metabolism

**Fig. 1 (abstract P-128).**
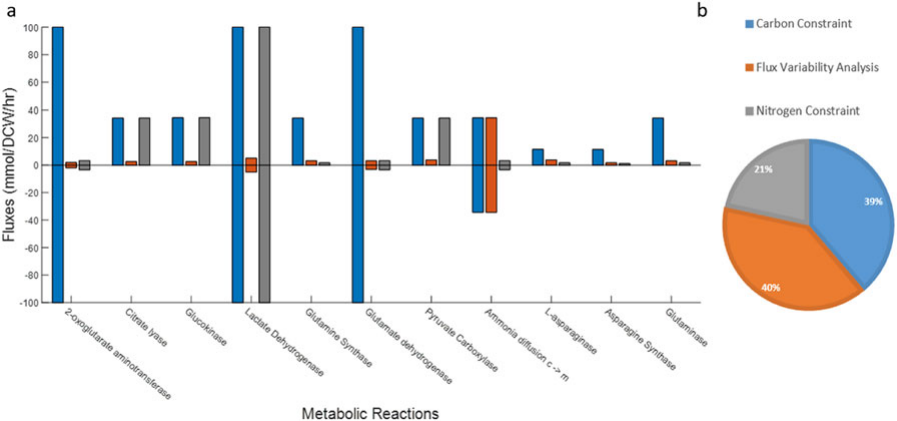
Flux variability analysis, ccFBA and nitrogen constrained FBA are used to predict bounds for the reactions of the CHO model. (a) Upper and lower bounds for certain enzymes and transport reactions taking part in the metabolism and (b) the percentage of constrained bounds using each method

### P-148 *In vivo* efficacy of recombinant factor VII produced in human cell line

#### Marcela C. Freitas^1^, Aline Bomfim^1^, Virginia Picanço-Castro^1^, Dimas Covas^1,2^

##### ^1^Blood Center of Ribeirão Preto, Ribeirão Preto, São Paulo, Zipcode 14051-140, Brazil; ^2^Medicine School, São Paulo University, Ribeirão Preto, São Paulo, Zipcode 14040-900, Brazil

###### **Correspondence:** Marcela C. Freitas (marcelafreitas@usp.br)

**Background**

Approximately 20 to 30% of patients with severe hemophilia A and 5% of patients with hemophilia B, who use factor VIII (FVIII) and factor IX (FIX) replacement therapy, respectively, develop antibodies that inhibit the activity of the infused factor. Patients with high-titer/high-responding inhibitors must be treated with bypassing agents that can achieve hemostasis [1]. Many studies have identified activated factor VII (FVIIa) as an attractive candidate for hemostasis, independent of FVIII/FIX, making this coagulation factor an alternative for hemophilia patients with inhibitory antibodies. Over the years, studies in Blood Center of Ribeirao Preto, Brazil, have been using human cell lines for production of recombinant coagulation factors. Sk-Hep-1 cells were chosen for rFVIIa production and showed, for a period of 6 months, an average of 8,03 IU/mL of rFVII yield. The goal of this study was to confirm the promising in vitro results of rFVIIa produced in Sk-Hep-1 cells using an in vivo model of hemophilia A mice.

**Materials and methods**

After the infusion of 8 mg kg -1 rFVII or NovoSeven, the mouse tail was amputated at a diameter of 3 mm under general anesthesia. Immediately upon lesion, the tail tip was submerged in isotonic saline solution (0.9%), which was kept at the physiological body temperature of the mice using a water bath, until hemostasis occurred [2]. The volume of total blood loss was calculated over an observation period of 15 min. The hemoglobin lost present in the isotonic saline was measured.

Recombinant FVII was administrated as single dose intravenously in retro-orbital vein at a dose of 3 μg/mouse. Blood samples were drawn at 2, 5, 30 and 60 min post-administration and then processed 10% sodium citrate (3.2% w/v) [3]. A commercially available ELISA-based system was used to evaluated human FVII antigen (FVII:Ag) plasma levels obtained from the hemophilic A mice.

**Results**

The results show that in both tests there was no statistical difference between NovoSeven and rFVII produced in the Sk-Hep-1 cell line.

**Conclusions**

In conclusion, this study reports the efficacy of rFVIIa produced in Sk-Hep-1 cell line in a *in vivo* model of hemophilia A mice when compared with the commercial product NovoSeven. These results suggest that this product can be used as an alternative in the treatment of hemophilic patients in the future.

**Acknowledgements**

The authors acknowledge the São Paulo Research Foundation – FAPESP (2015/19017-6), the Centro de Pesquisa, Inovação e Difusão (CEPID) (2013/08135-2), and the National Institute of Science and Technology in Stem Cell and Cell Therapy - INCTC for financial support.

**References**

1. Witmer C, Young G. Factor VIII inhibitors in hemophilia A: rationale and latest evidence. Ther Adv Hematol. 2013. 4(1): 59–72.

2. Holmberg HL, Lauritzen B, Tranholm M, Ezban M. Faster onset of effect and greater efficacy of NN1731 compared with rFVIIa, aPCC and FVIII in tail bleeding in hemophilic mice. J Thromb Haemost. 2009 Sep;7(9):1517-22.

3. Agersø H, Tranholm M. Pharmacokinetics and pharmacodynamics of rFVIIa and new improved bypassing agents for the treatment of haemophilia. Haemophilia. 2012 Jul;18 Suppl 5:6-10).

**Fig. 1 (abstract P-148).**
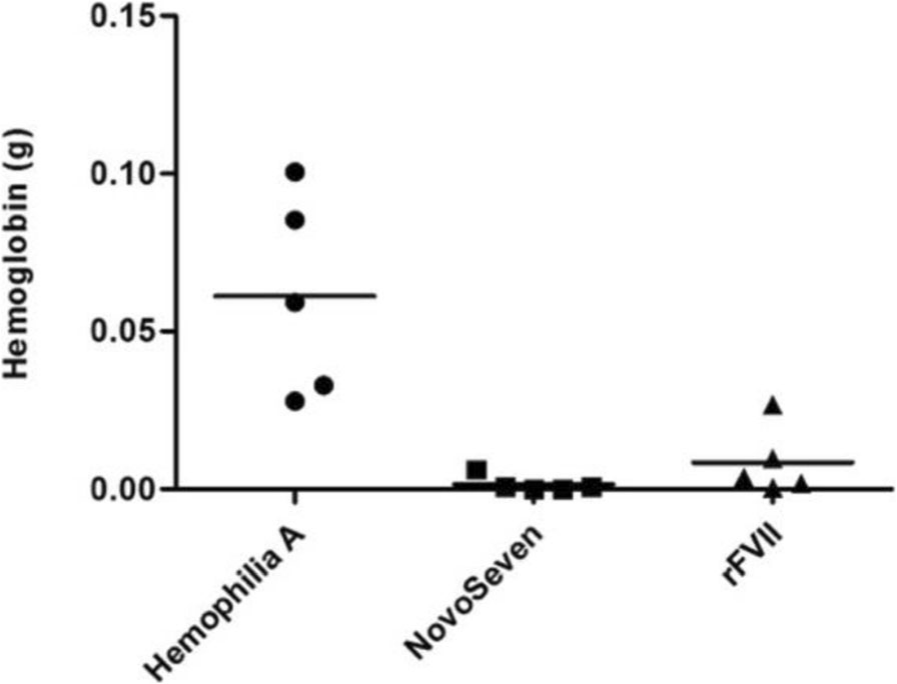
Homeostasis test. Dispersion plot showing the amount of hemoglobin lost by hemophiliac mice A after drug infusion followed by severe tail cut. There was no statistical difference between NovoSeven and rFVII (ANOVA and Bonferroni test, p-value ≤ 0.05)

### P-159 Impact of NaBu and RA on histones and nuclear signaling in CHO cells

#### Antonia Pries, Louise Schelletter, Thomas Noll, Raimund Hoffrogge

##### Institute of Cell Culture Technology, Bielefeld University, Bielefeld, Germany

###### **Correspondence:** Antonia Pries (antonia.pries@uni-bielefeld.de)

**Background**

Recently retinoic acid (RA) was found to increase CHO cell productivity, an effect long known for sodium butyrate (NaBu) and other short chain fatty acids [1]. Here we elucidate, by western blotting and a triple SILAC nLC Orbitrap experiment, the overlapping and diverging CHO signaling events correlated to histone modifications described to affect (product) gene transcription.

**Material and methods**

Effects of 150 nM RA (found to be optimal) compared to a 2 mM NaBu treatment of two CHO producer cell lines were investigated. Protein samples were taken at six timepoints (0, 15, 60, 180, 360 min) after agent addition. Histone fractions and nuclear proteins were extracted, separated by SDS- or acid urea (AU)-PAGE and analyzed by western blotting with antibodies against total and two phosphorylated variants of the retinoblastoma (Rb) protein and histone H3/H4 modifications. For a global view on the nuclear proteome and signaling, a triple SILAC cultivation with similar conditions followed by nLC ESI Orbitrap MS analysis with quantification on proteome and phosphoproteome level was carried out (Figure 1 a).

**Results**

Addition of NaBu or RA resulted in increased productivity and decreased cell growth as observed with other CHO cell lines before (Figure 1 b and c) [2,3]. For both agents changes in modification abundance of histone H4K5 acetylation and H3K4 trimethylation, correlated with elevated transcriptional activity, were detected in a time series. For NaBu no impact on amount or phosphorylation pattern of Rb protein, which functions as a central regulator of the cell cycle, was detected. In contrast for RA an increased amount of Rb and Rb phosphorylated at pS608 was detected, but no impact on Rb pS807/811. In the SILAC experiment differences and similarities between RA and NaBu in short term (60 min) influence on phosphoproteome were identified. RA addition resulted in 407 and NaBu caused 256 significantly regulated phosphopeptides after 1 h, with a p-value of 0.05 and a minimum fold change of 0.5. Further phosphoproteome analysis revealed signaling events described to be related to epigenetic processes like histone modifying enzymes and complexes. Methyltransferases SETD2, EZH2 or Suz12 showed changes in phosphorylation pattern. Also, effects at phosphosites of the acetyltransferase KAT8 and HDAC 1/2 were found (Figure 1 d). As a consequence, changes in histone methylation and acetylation arose. Further, short term effects on phosphorylation level of cell cycle affecting components like CDK2 were found, which in general correlates with alterations in cell growth.

To investigate changes (after 24 h) in the nuclear proteome, nearly 2000 proteins were quantified for samples of both agents. 243 were significantly differentially expressed after RA treatment and 355 in the NaBu approach (Table 1).

To create an overview of the affected pathways, we performed KEGG pathway mapping based on corresponding mouse identifiers. Mainly pathways of RNA transport, spliceosome and cell cycle were covered by our data. Detailed proteome analysis revealed effects on histone modifying enzymes like SETD2 methyltransferase or KAT8 acetyltransferase as already observed on phosphoproteome level. The implicated enzymes are responsible for H3K36me H3K4me (both activating), and H3K27me (inactivating) modifications. Also, diverse acetylations of histone 3 and 4 are affected (Figure 1 d).

**Conclusions**

RA and NaBu showed several similar effects on cultivation parameters, proteome and phosphoproteome level. Both caused reduced cell growth, an increased cell specific productivity and a higher abundance of transcription activating histone modifications. We proved similar and different dynamic changes in phosphorylation pattern of histone modifying enzymes 1 h after agent addition and differential expression of proteins in diverse nuclear pathways after 24 h.

**Acknowledgements**

We would like to thank the Australian Institute for Bioengineering and Nanotechnology, University of Queensland-Brisbane, Australia (AIBN) for providing the cell lines.

**References**

1. Rahimi-Zarchi M, Shojaosadati S A, Amiri M M, Jeddi-Tehrani M, Shokri F. All-trans retinoic acid in combination with sodium butyrate enhances specific monoclonal antibody productivity in recombinant CHO cell line. Bioprocess and Biosystems Engineering. 2018; 41 (7): 961–971.

2. Wippermann A, Rupp O, Brinkrolf K, Hoffrogge R, Noll T. Integrative analysis of DNA methylation and gene expression in butyrate-treated CHO cells. Journal of biotechnology. 2017; 257: 150‑161.

3. Müller B, Heinrich C, Jabs W, Kaspar-Schonefeld S, Schmidt A, Rodrigues de Carvalho N, Albaum S P, Baessmann C, Noll T, Hoffrogge R. Label-free protein quantification of sodium butyrate treated CHO cells by ESI-UHR-TOF-MS. Journal of biotechnology. 2017; 257: 87‑98.

4. Brink B, Seidel A, Kleinbölting N, Nattkemper T W, Albaum S. Omics Fusion - A Platform for Integrative Analysis of Omics Data. Journal of Integrative Bioinformatics 2016; 13: 296.

**Table 1 (abstract P-159).** Number of quantified/significantly regulated proteins

Proteins	RA	NaBu
quantified	1886	1948
significantly up regulated	100	306
significantly down regulated	143	49

**Fig. 1 (abstract P-159).**
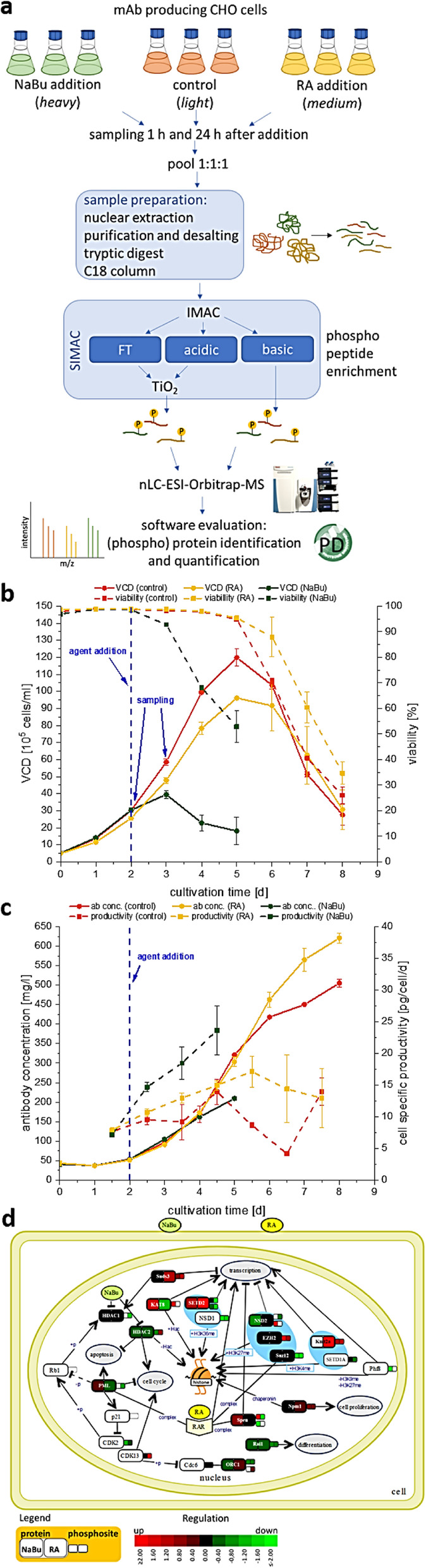
(a) Experimental setup of the triple SILAC cultivation for phosphoproteome (1 h) analysis of RA and NaBu effects; (b) Viable cell density [·10^5^ cells/ml] and viability [%] over time; (c) Cell specific productivity [pg/cell/d] and mAb concentration [mg/l]; (d) Combined phosphoproteome and proteome data mapped via in house Fusion software [4] on an individually created pathway in CellDesigner.

### P-160 Breakthrough CLD platform for bispecific antibodies expression

#### Séverine Fagète, Célia de Temmerman, Pauline Bernard, Cédric Steimer, Rémi Bussat, Pierre-Alain Girod

##### Selexis SA, Plan-Les-Ouates, CH-1288, Switzerland

###### **Correspondence:** Séverine Fagète (severine.fagete@selexis.com)

**Background**

Bispecific antibodies (bsAbs) represent a fast-growing class of molecules offering new therapeutic perspectives^1^. Their development is still hampered by the difficulty to produce such complex molecules composed of multiple polypeptide chains. Indeed, in contrast to classical IgG antibodies, which are produced under an homodimeric format, bsAbs offer a wide variety of heterodimeric formats^2^. The difficulty to produce them is even higher when by-side homodimer products cannot be distinguished with the heterodimer product of interest based on molecular weight sizing. If forced pairing by introducing point mutations such as knob into holes has been the focus of many efforts these last decades to maximize heterodimer formation, identification of clonal cell lines expressing high levels of heterodimer forms of bsAb has remained challenging. Currently, bsAbs are mainly produced *in vitro* after assembling half antibodies produced separately or *in vivo* from co-culture of two cell lines producing each half antibody^3^. Therefore, producing bsAb in a single-cell system, whatever the format, turns out to be the most attractive strategy.

**Materials and methods**

A bsAb constituted of a scFv-Fc moiety associated to a Fab-Fc was chosen as model. Co-transfection of independent SURE*tech* proprietary vectors, each harboring a different polypeptide, was used to optimize the chain-to-chain ratio.

Early screening of stable pools was done by automated microcapillary electrophoresis (μCE-SDS) using a LabChip GXII (Perkin Elmer) offering rapid and high throughput sizing, quantification and purity assessment. High-expressing stable clonal cell lines were evaluated for their performance (VCD, viability, productivity) using fed-batch cultures in different vessels including spin tube, shake flask, ambr15, ambr250 and bioreactor.

Characterization of the final purified product was assessed by protein A HPLC for quantification and by SEC HPLC to determine the aggregation level.

**Results**

Modulating relative expression of polypeptides according to chain-to-chain ratio allows for tuning both productivity and heterodimer content of the generated pools (Figure 1). Early screening of pool profiles by μCE-SDS enabled rapid identification of the pool containing the highest proportion of the bsAb heterodimer form (Figure 1). Combined with a fast process development platform, this fine characterization enabled the efficient isolation of the lead Research Cell Bank (RCB) candidates meeting both quantitative (PCD 36) and qualitative criteria (<2% aggregates).

Top clones were shown to maintain stable titer in the 5g/L range and stable heterodimeric composition above 92% at different scale and for >60 generations (Table 1 and 2).

**Conclusions**

By combining selective pairing expertise of partners with polypeptide chain ratio testing and qualitative-oriented screening, Selexis offers a unique CHO single-cell line development platform solution adapted to bsAb production.

More than 10 different bsAb formats have been produced using this technology reaching up to 6.5g/L in bioreactor with >95% purity of correct heterodimer product. Selexis SUREtechnology platform offers undeniable flexibility and has been successfully applied to the production of a large panel of multi-polypeptide molecules ranging from IgGs with poorly expressing chain to Ig- or Fc-fusion, alternative scaffold, and viruses, with a gene to GMP service in 9 months.

**References**

1. Suurs FV, Lub-de-Hooge MN, de Vries EGE, de Groot DJA. A review of bispecific antibodies and antibody constructs in oncology and clinical challenges. Pharmacol. Ther. 2019; in press.

2. Brinkmann U, Kontermann RE. The making of bispecific antibodies. MAbs. 2017; 9(2):182-212.

3. Shatz W, Domingos N, Dutina G, Wong AW, Dunshee DR, Sonoda J, Shen A, Scheer JM. An efficient route to bispecific antibody production using single-reactor mammalian co-culture. MAbs. 2016; 8(5):1487-1497.

**Fig. 1 (abstract P-160).**
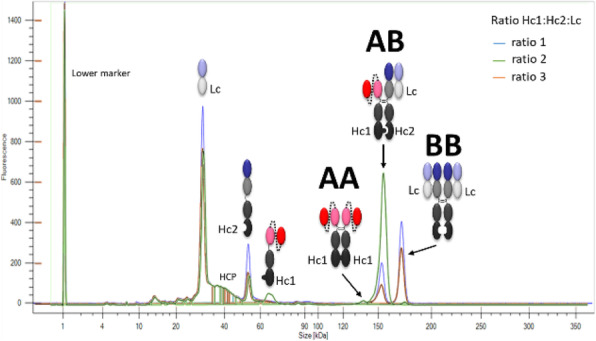
Screening of purity profiles of generated bsAb pools by μCE-SDS

**Table 1 (abstract P-160).** Scalability of production of bsAb from a small scale vessel to a large production process

Vessel	Titer (g/L)^a^	Purity (%)^b^		
		AA	AB	BB
Spin Tube	4.9	1.4	92.7	5.9
Shake Flask	4.2	0.7	95.5	3.8
Ambr15	5.6	0.6	96.5	2.9
Ambr250	4.9	1.0	93.5	4.9
Bioreactor	4.9	1.1	97.6	1.6

^a^ total protein HPLC titer (homodimers + heterodimer). Representative of up to 6 different clone samples

^b^ homodimer (AA, BB) versus heterodimer (AB) purity determined by μCE-SDS. Representative of up to 4 clone sample repeats.

**Table 2 (abstract P-160).** Stability of bsAb production in 10 day fedbatch culture in shake flask

Number of generations	PCD^a^	titer^b^	purity^c^
5	21.1	4.1	96.0
35	14.9	4.8	97.0
65	16.7	4.7	96.0

^a^ Productivity per cell per day (PCD)

^b^ total protein HPLC titer (homodimers + heterodimer)

^c^ heterodimer purity determined by μCE-SDS

### P-166 Optimized Combination of Genetic Elements Enhancing mAb Production

#### Kristin Thiele^1^, Beate Stern^2^, Michael Baunach^1^, Linda Roth^1^, Juliana Schubert^1^, Magdalena Moos^1^, Christoph Zehe^1^

##### ^1^Sartorius Stedim Biotech GmbH, 88471 Laupheim, Germany; ^2^UniTargetingResearch AS, 5006 Bergen, Norway

###### **Correspondence:** Kristin Thiele (kristin.thiele@sartorius-stedim.com)

**Background**

To satisfy the continuous demand for high-titer bio-pharmaceutical producing cell lines, enhancing the level of mRNA transcript coding for a protein of interest, is one possible engineering strategy. In mammalian cells, high-level translation and corresponding protein yield also crucially depend on mRNA stability and localization, as well as on the efficient initiation of entry of the protein into the endoplasmic reticulum. The presence of specific genetic elements, e.g. appropriate signal peptide (SP) coding regions and 5′ and 3′ untranslated regions (UTRs) play an important role in this respect.

**Materials and Methods**

The influence of individual 5′ and 3′UTRs and SPs in different expression cassettes (UTR®Betatech, supplied by UniTargetingResearch AS) for heavy chain (HC) and light chain (LC) in a double-gene vector expressing an IgG1 model antibody was investigated in fed-batches using a CHO-DG44 host cell line. After combining the most promising elements with Sartorius Stedim Biotech’s signal peptide (SP9), the optimized expression vector (vector 4.0) was tested for three different mAbs. For this purpose, stable pools were generated under MTX-free conditions, and fed-batch performance was monitored. Individual cell pools were selected and clones generated by FACS-sorting. The top 12 clones were analyzed in fed-batch experiments at shake flask or Ambr® 15 scale. Top clones identified by clone evaluation were then run in a fed-batch process in UniVessel® 5L Bioreactor system, based on the Sartorius Stedim Biotech’s CHO production process.

**Results**

The screening of different element combinations showed a high impact on mAb yield in fed-batch. Best results were obtained with the modified expression vector #11, containing a specific combination of 5′UTR, 3′UTR, SP and polyadenylation site for HC and LC, respectively, recommended by UniTargetingResearch. Pool fed-batch titers of the model IgG were increased up to 50% over those obtained with the standard vector (Table 1).

In the second approach, the new developed vector 4.0, consisting vector combination #11 and SP9 achieved an even higher increase ranging from 1.2 fold to 4.3 fold in average pool fed-batch titers for three different mAbs (mAb1-3). Top clones harboring vector 4.0 showed an average titer of 5.2 g/L for all three products and a 2.4 fold increase in mean clone titer compared to standard (Figure.1).

When transferring mAb-3 top clones to the UniVessel® 5L Bioreactor system, for all tested products, the viability of 80% could be maintained until process day 14 and the viable cell densities reached up to 250x10^5^ cells/mL at process day 7-9. Product titers evaluated in Ambr® 15 or shake flasks were successfully confirmed, reaching levels of around 5 g/L for the three mAbs (Table 2).

**Conclusion**

Our screening demonstrates that specific genetic elements significantly influence final protein concentration achievable in CHO-DG44 cells. The use of UTR®Betatech vector technology in combination with Sartorius Stedim’s SP9 lead to enhanced clone fed-batch titer, up to 119% compared to standard (= 2.37 g/L). Thus, the new expression vector 4.0 contributes to the continuous improvement of the standard expression system for mAb, ensuring high-level titer cell lines in Sartorius Stedim Biotech’s MTX-free cell line development process.

**Table 1 (abstract P-166).** Percentage increase/decrease in final fed-batch titers of CHO-DG44 pools using UTR®Betatech element combinations

Vector combination	1	2	3	4	5	6	7	8	9	10	11
Mean titer [%]	-11	-6	0	23	19	-6	1	-45	-10	-88	50

**Fig. 1 (abstract P-166).**
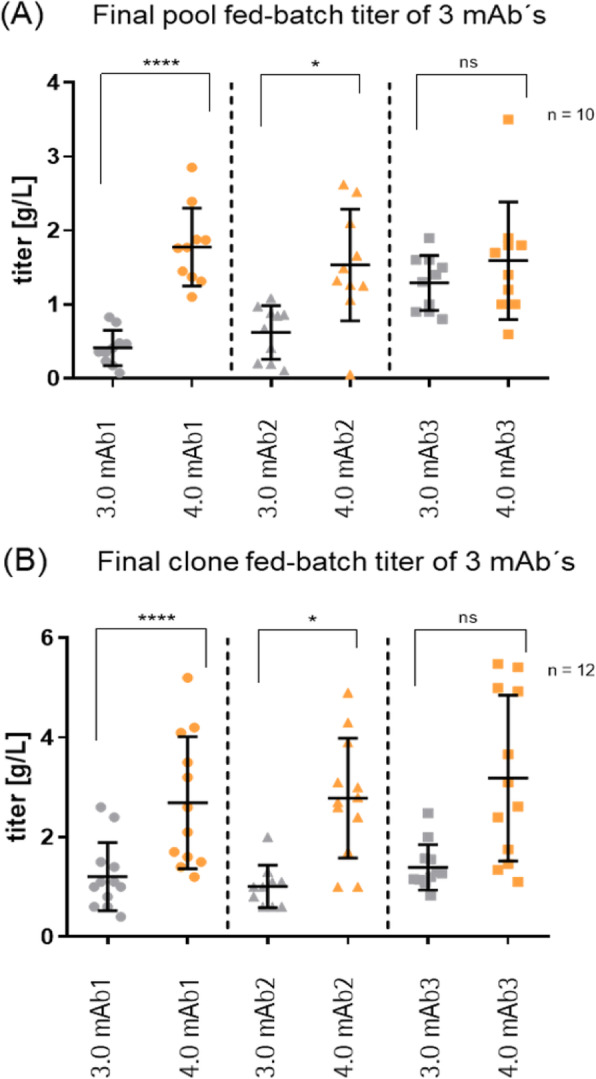
Final fed-batch titers [g/L] of pools (A) and top clones (B) in Ambr® 15 (mAb1 and mAb2) and shake flask (mAb3)

**Table 2 (abstract P-166).** Time to peak and viable cell concentration at peak, final viability and titer of top clones (on day 14) expressing mAb1-3 in fed-batch process using UniVessel® 5L Bioreactor system

	time to peak VCC [d]	peak VCC [10^5^cells/mL]	final viability [%]	final titer [g/L]
mAb1	7	253.7	79.8	5.0
mAb2	8	132.9	86.1	5.7
mAb3	9	197,0	91.3	4.9

### P-174 Biologicalisation in medicine and manufacturing: A nature-based digital revolution

#### Bill G. Whitford (bill.whitford@GE.com)

##### Biosciences AB, GE Healthcare, Uppsala, Sweden

**Background**

Biologicalisation uses such advances as 4.0 principles [1] in concert with bio-inspired materials, chemistries and functions to support efficient and sustainable manufacturing [2]. Processes are enhanced by the harmonization of newer manufacturing principals with advanced bioengineering understandings, systems and materials. This is enabling efficient, clean and robust production supporting sustainable, circular and global economies.

An example of this developing capability is a 2018 Nobel Prize winner, Frances H. Arnold. She invented systems directing the engineering of enzymes which now support the sustainable manufacturing of such diverse products as fuels, foods and pharmaceuticals [*3*].

This biological transformation of manufacturing begins with bio-inspired systems materials, chemistries, and catalysts and includes biological structures, metabolisms and even living cells. It continues with bio-integrated process design – from materials and processes to equipment and facilities (Figure 1).

**Materials and methods**

The history of acetic acid (HOAc) manufacturing illustrates our evolution from biological, to synthetic, to biologicalised manufacturing.

The old German method was a natural means of production that percolated an alcoholic solution through a tower of wood shavings harboring *Acetobacter*. Next, we generated it synthetically, using energy-demanding processes involving chlorinated hydrocarbon intermediates and troublesome waste.

Today, genetic engineering and digital biomanufacturing are promising a more sustainable, yet efficient, cell- and enzyme-based approaches. Benefits include lower energy requirements, toxic metals and solvents-free waste – and the possibility of using organic municipal wastes and agriculture residues as carbohydrate sources.

Biomanufacturing is slowly incorporating such “4.0” initiatives as digitalization, automation and operations integration. Ultimate goals include the industrial internet of things (IoT), advanced monitoring, machine learning and artificial intelligence [4]. For example, GE Healthcare is developing “digital twins” of mixers and bioreactors for both process development and the prediction of large-scale production events.

Cell-based medical systems are another implementation of this biologicalisation and begin with the fermenter and bioreactor-based production of biopharmaceuticals. The success of such cell-based therapies as tisagenlecleucel, an FDA-approved CAR T-cell therapy, illustrates the next step. 3D bioprinting and newer microfluidics and advanced biochemistries are now replacing both living animals and toxic chemical-based assays with *ex vivo* cell-based systems that provide gains in both analytical power and environmental sustainability. Advantages over live animals or their tissues include economy, efficiency and modifiability. They are also more controllable, amenable to multiplexed monitoring, and finally, are more humane.

**Results**

An existing example of biomimetic-structure based biologicalisation is in the manufacturing of Lipitor. Pfizer had previously reduced organic waste in the process by 65% [4]. Bruce Lipshutz (UCSB) further improved it by developing a self-assembling surfactant in an aqueous medium [5]. His micelles mimic a living cell in the sequestering of reactions and components from the ambient environment. These structured reactions, such as in carbon-carbon couplings, require only parts per million transition metals in an organic solvent-reduced or -free environment. Biofabrication in the context of biologicalisation is a new approach to bio-based raw material design [6] and includes such custom manufacturing techniques as 3D cell culture and bioprinting of cells.

**Conclusions**

The many contributing technologies in this new approach to manufacturing is complex. Biologicalisation begins with classical genetic engineering and evolves to Industry 4.0 directed synthetic and computational biology**.** It builds manufacturing systems from complex and bio-hybrid structures, parts, and devices using advanced biochemistry – and from such digital technologies as *in silico* modeling. Biologicalisation is also opening the door to more effective and sustainable drug development techniques, as well as medical treatments and assays.

**References**

1. Lyndon, B. Industry 4.0 - Only One-Tenth of Germany's HighTech Strategy. Automation.com [Online] https://www.automation.com/automation-news/article/industry-40-only-one-tenth-of-germanys-high-tech-strategy.

2. Byrne G. et al. Biologicalisation: Biological transformation in manufacturing. CIRP J. Manuf. Sci. Tech. 2018; 21:1–32.

3. Vogel, G. ‘Revolution based on evolution’ honored with chemistry Nobel. Science News (2018). [Online.] https://www.sciencemag.org/news/2018/10/revolution-based-evolution-honored-chemistry-nobel.

4. Sanso, C.. Solvents and sustainability, Chemistry World [Online].

https://www.chemistryworld.com/features/solvents-and-sustainability/3008751.article

5. Lipshutz, B. The Hydrophobic Effect Applied to Organic Synthesis: Recent Synthetic Chemistry “in Water” Chemistry, 2018; [24]26:6672-6695.

6. Quaglia, D. BIOFABRICATE: Biotech and Synbio for the Production of New Sustainable Materials. PLOS Blogs. https://blogs.plos.org/synbio/2016/11/24/biofabricate-biotech-and-synbio-for-the-production-of-new-sustainable-materials/ (2016). Accessed 22 February 2019.

**Fig. 1 (abstract P-174).**
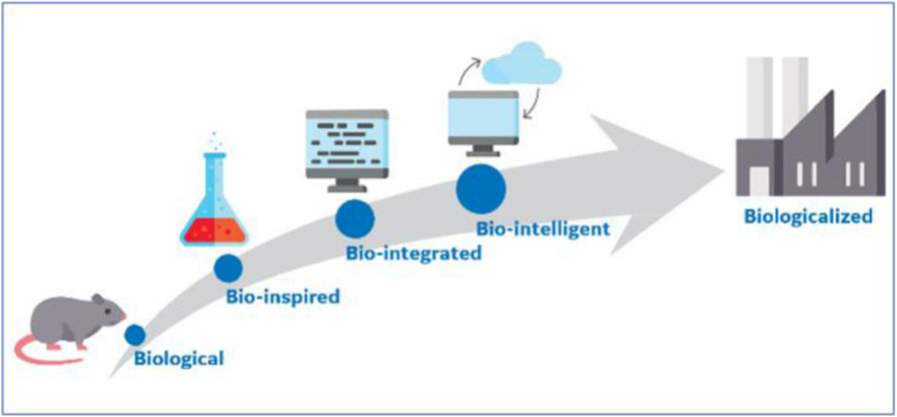
Natural to biologicalised. Biological: animals, plants, bacteria, fungi, chemistry and natural systems. Bio-Inspired: materials, surfaces, medium, pathways, chemistries, catalysts. Bio-Integrated: design, processes, assemblies, reactions, equipment, facilities. Bio-Intelligent: systems, organization, supply chains, global circular economy

### P-197 Does CHO gene expression change in response to expressed mAb variant?

#### Linda Schwaigerlehner^1^, Samia Akhtar^2^, Rachel Scholey^3^, Mauro Torres^2^, Alan J. Dickson^2^, Renate Kunert^1^

##### ^1^Department of Biotechnology, University of Natural Resources and Life Sciences, Vienna, Austria; ^2^Manchester Institute of Biotechnology, Faculty of Science and Engineering, University of Manchester, Manchester, UK; ^3^Bioinformatics Core Facility, University of Manchester, Manchester, UK

###### **Correspondence:** Linda Schwaigerlehner (linda.schwaigerlehner@boku.ac.at)

**Background**

Although recombinant Chinese hamster ovary (CHO) cell lines are developed and cultivated under comparable conditions, the monoclonal antibody (mAb) expression can differ significantly. As IgGs are secretory proteins, we assumed that insufficient secretion might result from the intrinsic antibody structure and its interaction with cellular compartments of the folding and secretion machinery [1,2].

We analyzed antibody secretion as well as differences in gene expression patterns of CHO cell lines producing a difficult-to-express (DTE) antibody 2G12 and its germline variant 353/11.

**Materials and Methods**

To exclude positional effects during gene transcription we inserted all antibody coding regions into a pre-defined chromosomal locus with invariable gene copy numbers by using an engineered CHO K1 as host applicable for recombinase-mediated cassette exchange (RMCE).

To compare 2G12 and 353/11 producing clones semi-continuous perfusion was performed in small scale bioreactors to ensure a comparable cultivation condition for analysis of the cellular transcriptome. CD-CHO medium (4 mM L-Gln, 15 mg/L phenol red, 2 μM ganciclovir) was exchanged daily to provide identical environment for all cultures. Consumption/production of key metabolites was monitored to evaluate growth behavior. RNA sequencing was performed to evaluate and identify differences in gene expression patterns which are linked with higher productivity. To avoid effects due to nutrient limitations, samples for RNA-seq were taken 4 hours after medium exchange.

**Results**

Both cell lines showed a maximum cell concentration of approx. 40∙10^6^ c/mL. The viabilities remained high throughout the whole process. 353/11 showed a significantly higher cell-specific mAb production rate than 2G12. Concentrations and consumption/production rates of key metabolites were comparable between 2G12 and 353/11 throughout the semi-continuous perfusion experiment.

RNA-seq allows accurate screening of RNA expression and expands our understanding of biological processes involved in antibody expression. Samples in quasi-steady state of the semi-continuous perfusion (day 6) were analyzed for identification of specific gene expression profiles between the two isogenic CHO cell lines. Principle component analysis of the 500 most variable genes shows clear separation of the cell lines. Comparison of RNA-seq results of DTE antibody 2G12 and its germline variant 353/11 revealed differences in their gene expression profiles.

Subcellular localization of differentially expressed genes between 2G12 and 353/11 was analyzed. The top identified gene ontology terms correlated with the plasma membrane (Figure 1). Besides alteration of the plasma membrane, a considerable number of genes with uncertain function (LOCs) were up- or downregulated between 2G12 and 353/11.

**Conclusions**

Despite comparable growth behavior of the isogenic CHO cell lines, differences in antibody expression were observed. RNA-seq allowed clear discrimination between both cell lines. Identification and localization of up- and downregulated genes between 2G12 and 353/11 provide possible new gene targets for future recombinant protein production studies.

**Acknowledgements**

This work was supported by the Austrian Science Fund (FWF) under (Grant number P 25056) and the PhD program BioToP (Biomolecular Technology of Proteins) funded by FWF under (Project W1224).

**References**

1. Mayrhofer *et al.* Accurate comparison of antibody expression levels by reproducible transgene targeting in engineered recombination-competent CHO cells. Appl. Microbiol. Biotechnol. 2014; 98:9723-9733.

2. Sommeregger *et al.* Proteomic Differences in Recombinant CHO Cells Producing Two Similar Antibody Fragments. Biotechnol. Bioeng. 2016; 113:1902-1912.

**Fig. 1 (abstract P-197).**
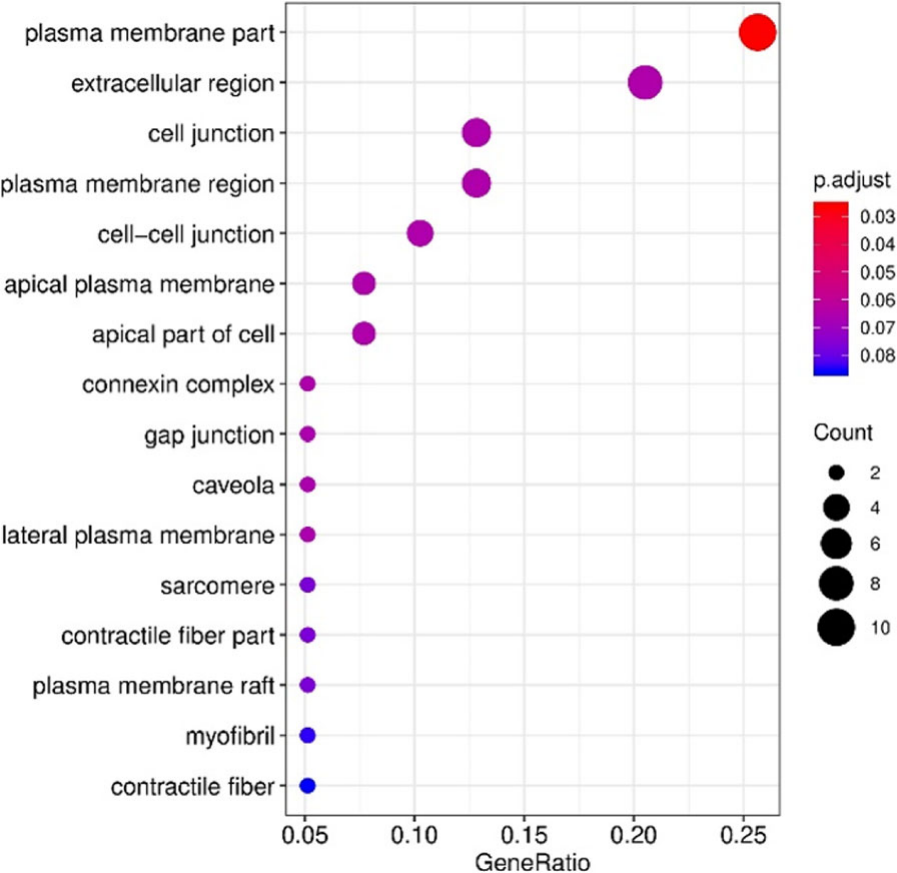
Gene ontology (GO) analysis of cells expressing 2G12 IgG and 353/11 IgG within the cellular component (CC) category

### P-215 Extracellular vesicle characterization during lentiviral production

#### Aline Do Minh, Amine Kamen

##### Bioengineering Department, McGill University, Montreal, QC, Canada

###### **Correspondence:** Aline Do Minh (aline.dominh@mail.mcgill.ca)

**Background**

Lentiviral vectors (LVs) are a powerful tool for gene therapy. Human embryonic kidney cells (HEK293) have been used extensively as a platform for viral vaccine and viral vector production. Similar to most cells and body fluids, HEK293 cells release extracellular vesicles (EVs). EVs released by cells share similar size, biophysical characteristics and even biogenesis pathway with cell-produced enveloped viruses, making it a challenge to efficiently separate EVs from LVs. Thus, EVs become “impurities” in the context of gene therapy viral vectors, as they co-purify with LVs during downstream processing. The aim of this study is to characterize EVs during LV production and to identify their changes upon lentiviral production from a proteomic, lipidomic and transcriptomic perspective.

**Materials and methods**

The inducible HEK293SF-LVP-CMVGFPq-92 expressing GFP suspension cell line (Clone 92), developed at the National Research Council Canada (NRC), was used to produce lentiviral vectors [1] (Figure 1). The first step is to optimize the EV isolation process and to separate EVs from LVs as the proteomic and RNA profiles are clearly affected by the isolation method. After isolation, EVs are characterized by transmission electron microscopy (TEM) and western blot (WB). Additionally, nucleic acid content is assessed as it is critical to quantify the amount of residual host cell DNA in biologics destined to the clinic. Mass spectrometry (MS) is used to determine the proteomic content of EVs.

**Results**

Preliminary data showed size exclusion chromatography (SEC) as an improved isolation method when compared to the gold standard ultracentrifugation. SEC yielded more intact and purer EVs as shown by TEM. WB and MS analysis confirmed the presence of EV enriched proteins (e.g. Alix, TSG101, CD9, CD63 and CD81). MS results showed that the presence of some proteins was medium dependent. However, it was observed that protein detection by MS was affected by sample preparation (e.g. addition of detergent). Results also showed that GFP was detected in EVs since the cell line used in this study expresses GFP constitutively.

**Conclusions**

An optimized process combining UF/DF and SEC yielded EVs with adequate volumes and concentrations for further characterization. Different sets of proteins were revealed with/without lentiviral induction. Fractionation leads to more enriched EVs in the first fractions (lower iodixanol concentration), while the last fractions (higher iodixanol concentration) are enriched in functional LV. A set of LV viral proteins were detected in all LV/EV fractions, but none were found in EV fractions. Quantitation and sequencing of genomic content are to come. Further mass spectrometry analyses are conducted to analyse EVs isolated with the optimised process. These studies should allow to identify EVs markers upon induction of LV production and help in assessing product safety and identity.

**Acknowledgements**

We would like to thank Alexandra Star and Sue Twine from NRC for the proteomics analyses.

We acknowledge the support of the Natural Sciences and Engineering Research Council of Canada (NSERC).

**Reference**

1. Manceur, AP, Kim, H, Misic, V et al. Scalable lentiviral vector production using stable HEK293SF producer cell lines. Hum Gene Ther Methods. 2017; 28(6):330-339.

**Fig. 1 (abstract P-215).**
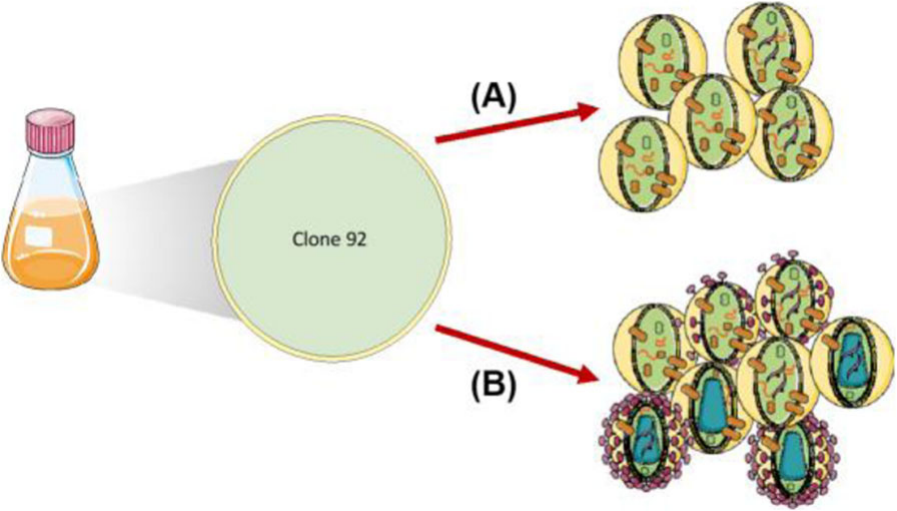
Clone 92 cells are cultivated in suspension with serum-free medium. (A) Without induction, cells release EVs that randomly incorporate GFP. (B) Addition of doxycycline and cumate induces the production of LV and intermediates entities

### DP-219 Development of perfusion suspension Vero culture process for high titer production of vesicular stomatitis virus

#### Chun Fang Shen^1^, Rénald Gilbert^1^, Claire Guilbault^1^, Xiuling Li^2^, Mehdy Elahi^1^, Sven Ansorge^1^, Amine Kamen^1,3^, Frank van Lier^1^

##### ^1^Human Health Therapeutics Research Center, NRC, Canada; ^2^China National Biotec Group, China; ^3^McGill University, Canada

###### **Correspondence:** Chun Fang Shen (chunfang.shen@cnrc-nrc.gc.ca)

**Background**

Vero cells are considered as the most widely accepted continuous cell line by the regulatory authorities for the manufacture of viral vaccines for human use [1]. The growth of Vero cells is anchorage-dependent. Scale-up and manufacturing in adherent cultures are labor intensive and complicated [2]. Adaptation of Vero cells to grow in suspension will simplify subcultivation and process scale-up significantly, and therefore reduce the production cost. Here we report on a successful adaptation of adherent Vero cells to grow in suspension and the development of perfusion culture process for high yield productions of vesicular stomatitis virus (VSV) at high cell density.

**Materials and methods**

The origin of Vero cells used in this study was ATCC CCL-81. ProVero 1 serum-free medium (Lonza) and IHM03, a serum-free and animal component-free medium developed in-house, were used to grow Vero cells and their adaptation to suspension culture. A recombinant VSV was selected as a model virus to evaluate the virus productivity of Vero cells.

Adherent Vero cell culture was maintained with either ProVero 1 or IHM03 media in T-flasks, and then adapted to grow in suspension in IHM03 medium using our proprietary adaptation technology. The adherent and suspension adapted Vero cells at various passages were authenticated using Short Tandem Repeat (STR) analysis. Suspension adapted Vero cells at a passage number of 163 were assayed for tumor formation according to a FDA Standard Protocol.

Production of VSV in both adherent and suspension Vero cells was evaluated by infecting cultures with VSV at an MOI of 0.1 with or without medium replacement. Supernatant of infected cultures was harvested at different times post infection and extra-cellular virus titer was measured by TCID50% in duplicate in 293A cells.

The perfusion culture of suspension Vero cells was performed in a 3 L bioreactor using a BioSep 10L acoustic filter as cell retainer. The culture was grown to high cell density under perfusion condition, and then infected with VSV.

**Results**

The adherent Vero cells were successfully adapted to grow in suspension in IHM03 medium. The maximum cell density in shake flask batch culture was about 2.5x10^6^ cells/mL, which is similar to or better than the cell density reported by others in microcarrier cultures using commercial serum-free media. Much higher cell density (8x10^6^ cells/mL) was achieved in a batch culture when three volumes of the culture medium were replaced during the batch culture process. The doubling time of suspension cell was 40 to 44 hours.

Authentication of both adherent and suspension Vero cells from various stages indicates that all Vero cell samples had 100% concordance with the Vero DNA control sample, indicating the suspension cells maintained their genetic stability. Furthermore, suspension Vero cells at a passage number of 163 were not found to be tumorigenic.

The data in Table 1 revealed that the suspension cell culture showed a better productivity of VSV than the adherent ones. In addition, the suspension culture could be infected at higher cell densities, thus improving the volumetric virus productivity. More than one log of increase in the VSV productivity was achieved in a 3L bioreactor perfusion culture infected at a cell density of 6.8x10^6^ cells/mL.

**Acknowledgements**

Authors greatly appreciate the support of Stephane Lanthier for the bioreactor operation, and Mélanie Leclerc for the Vero culture work.

**References**

1. Knezevic I, Stacey G, Petricciani J, Sheets R, Substrates W. Evaluation of cell substrates for the production of biologicals: Revision of WHO recommendations. Report of the WHO Study Group on Cell Substrates for the Production of Biologicals, 22–23 April 2009, Bethesda, USA. Biologicals 2010;38:162–169.

2. Madeline B, Ribaud S, Xenopoulos A, Simler J, Schwamborn K, Léon A. Culturing a duck ES-derived cell line in single-use bioreactors: a rapid, efficient, and cost-effective vaccine manufacturing system based on suspension culture. BioProcess International, 2015;13(3)S:26-33.

**Table 1 (abstract P-219).** Production of VSV in adherent and suspension adapted Vero cell cultures

Cell density at infection (x10^6^cells/mL)	VSV titer (x10^8^ TCID50/mL)
Adherent culture	
0.3	4.1±0.9
1.0	2.9±0.7
Suspension culture	
0.5	9.0±2.1
1.0	11.6±0.9

**Fig. 1 (abstract P-219).**
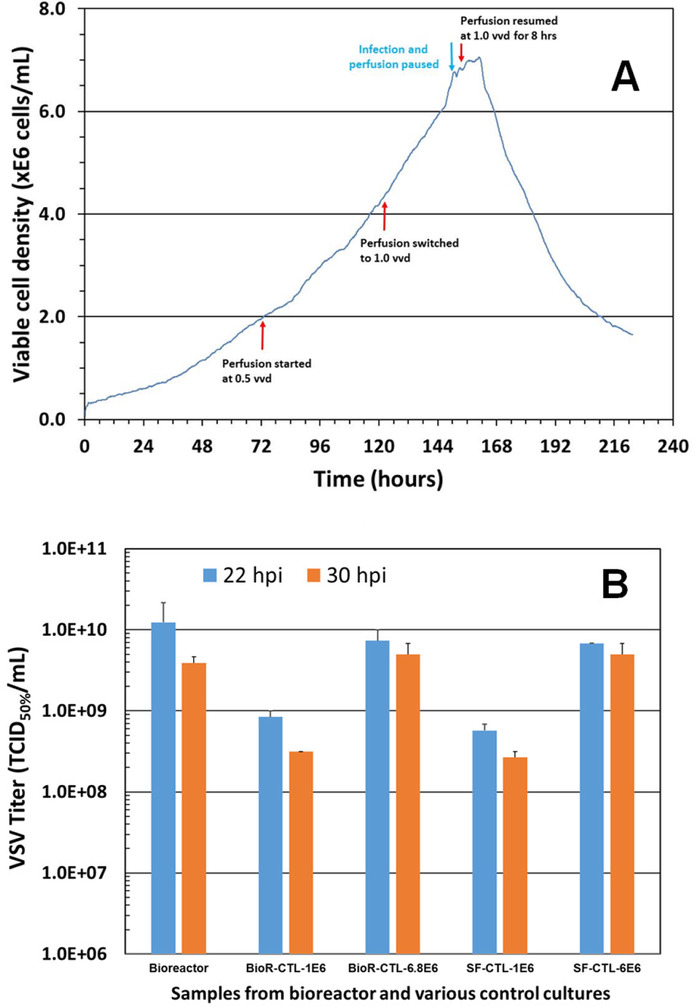
The cell density in the bioreactor reached 6.8x10^6^ cells/mL after 2 days of perfusion at 0.5 vvd and another day at 1 vvd before the virus infection (Fig. 1A). Infected culture taken from the bioreactor was maintained in shake flask as bioreactor controls (BioR-CTLs). In parallel, cells grown in shake flask (never be in bioreactor) with medium replacement was also infected as shake flask controls (SF-CTLs). The above infected cultures were also diluted with fresh medium to 1x10^6^ cells/mL (1E6) as references. The VSV titer in the bioreactor culture was similar to that achieved in the shake flask control cultures (-6E6) and was nearly one log higher than that in the control culture infected at 1x10^6^ cells/mL (Fig. 1B)

### P-225 Production of influenza virus-like particles in insect cells

#### Hideki Yamaji, Takuya Matsuda, Toshikazu Tanijima, Kyoko Masumi-Koizumi, Tomohisa Katsuda

##### Department of Chemical Science and Engineering, Graduate School of Engineering, Kobe University, 1–1 Rokkodai, Nada, Kobe 657–8501, Japan

###### **Correspondence:** Hideki Yamaji (yamaji@kobe-u.ac.jp)

**Background**

Vaccines have been widely used as one of the most effective ways of controlling and preventing infectious diseases including viral infections. Structural proteins of viruses, such as envelope and capsid proteins, spontaneously assemble into particulate structures similar to authentic virus particles. Based on this property, virus-like particles (VLPs) can be produced by expressing such viral surface proteins using recombinant DNA technology [1]. VLPs can elicit strong immune responses because they densely present viral antigens in an authentic conformation on their surface. VLPs are safe and non-replicating because they assemble without incorporation of viral DNA or RNA. Among various recombinant protein expression systems, the baculovirus–insect cell system has been widely used for the VLP production, but it has several inherent limitations including contamination with progeny baculoviruses. Recombinant insect cells can be employed as an attractive alternative to the baculovirus–insect cell system [1, 2]. In the present study, we investigated the production of influenza VLPs in recombinant insect cells.

**Materials and methods**

The DNA fragments encoding hemagglutinin (HA) and matrix protein 1 (M1) of an influenza virus A (H1N1) were individually cloned into the plasmid vectors pIHAbla and pIHAneo. The pIHAbla and pIHAneo contained the *Bombyx mori* actin promoter downstream of the *B. mori* nucleopolyhedrovirus (BmNPV) IE-1 transactivator and the BmNPV HR3 enhancer for high-level expression, together with either a blasticidin or neomycin resistance gene for use as a selectable marker, respectively [3]. After co-transfection with the resultant plasmids using FuGENE 6 transfection reagent (Promega, USA), *Trichoplusia ni* BTI-TN-5B1-4 (High Five) cells were incubated with blasticidin and G418, and cells resistant to the antibiotics were selected. Obtained recombinant High Five cells were cultivated in static or shake-flask cultures with a serum-free medium Express Five SFM (Thermo Fisher Scientific, USA). Culture supernatants were analysed by western blotting, sucrose density-gradient sedimentation analysis, negative stain transmission electron microscopy, and hemagglutination assay.

**Results and discussion**

High Five cells were co-transfected with a set of plasmid vectors individually containing either the influenza A virus HA or M1 gene along with either a blasticidin or neomycin resistance gene, followed by incubation in the presence of blasticidin and G418. Colonies of cells resistant to the antibiotics were efficiently obtained around 2 weeks after co-transfection. Western blot analysis of a culture supernatant revealed that High Five cells co-transfected with the HA and M1 genes secreted HA and M1 molecules equivalent to the authentic influenza A virus HA and M1 (Figure 1). Sucrose density-gradient sedimentation analysis and transmission electron microscopy of the culture supernatant suggested that secreted HA and M1 molecules were produced in a particulate form. Hemagglutination assay using chicken erythrocytes showed hemagglutination activity in the culture supernatant, indicating that secreted HA molecules were biologically functional. These results demonstrate that recombinant insect cells may offer a promising approach for efficient production of influenza VLPs.

**References**

1. Yamaji H: Suitability and perspectives on using recombinant insect cells for the production of virus-like particles. *Appl Microbiol Biotechnol* 2014, 98:1963–1970.

2. Yamaji H: Production of antibody in insect cells. In *Antibody expression and production. Cell engineering. Volume 7.* Edited by Al-Rubeai M. Dordrecht, Netherlands: Springer Science + Business Media; 2011. p. 53–76.

3. Yamaji H, Manabe T, Watakabe K, Muraoka M, Fujii I, Fukuda H: Production of functional antibody Fab fragment by recombinant insect cells. *Biochem Eng J* 2008, 41:203–209.

**Fig. 1 (abstract P-225).**
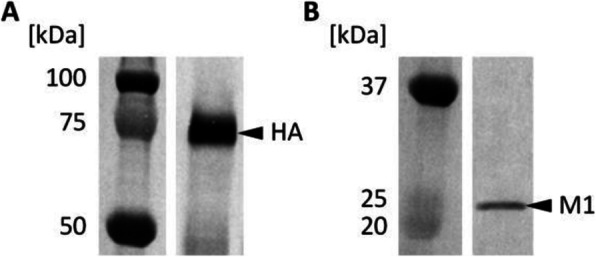
Western blot analysis of a culture supernatant using anti-influenza A virus hemagglutinin (HA) H1 antibody (A) and anti-influenza A virus M1 antibody (B). High Five cells were co-transfected with influenza A virus HA and M1 genes

### P-300 Generation of Trastuzumab Antibody-Drug Conjugates at different DARs

#### Joan Miret^1^, Marc Camps^2^, Mercè Farràs^2^, Ramón Román^1^, Isaac Priego^1^, Beatriz Bataller^1^, Antoni Casablancas^1^, Martí Lecina^1^, Jordi J. Cairó^1^

##### ^1^Chemical, Biological and Enviromental Engineering Department, UAB, Barcelona, Spain; ^2^Farmhispania SA, Montmeló, Spain

###### **Correspondence:** Joan Miret (joan.miret@uab.cat)

**Background**

Antibody Drug Conjugates (ADCs) represent an increasingly important tool for the treatment of cancer. One of their main features is the drug payload, characterized through the Drug Antibody Ratio (DAR). Approved ADCs tend to have a DAR about 3-4 and are mostly heterogeneous, consisting in a mixture of differently loaded ADC species, containing unconjugated antibodies (low therapeutic activity) as well as highly-loaded species (that could aggregate showing also low therapeutic activity). Homogeneous ADCs, where the cytotoxic drug is conjugated in a site-directed way to the antibody, have a wider therapeutic window.

HEK293 cells were selected for the production of antibodies, since the studies and knowledge of such cell lines is rapidly increasing [1, 2]. We have generated a HEK293 line producing Trastuzumab, which has been conjugated to the vcMMAE drug to obtain a DAR 4 heterogeneous ADC and a DAR 8 homogeneous ADC. We have also generated a HEK293 line producing the Trastuzumab cysteine-engineered variant Trastuzumab_cys114 [3]. This variant contains an added cysteine residue for the site-directed conjugation to vcMMAE, obtaining a DAR 2 homogeneous ADC. The *in vitro* activity of the differently loaded ADCs has been compared.

**Material and Methods**

Trastuzumab heavy (HC) and light (LC) chains sequences were cloned in the tricistronic pTRIpuro3 plasmid, which was stably transfected in HEK293 cells [4]. For homogeneous conjugation, derivate Trastuzumab_cys114 (Ala substituted by Cys, position 114 of HC, Kabat numbering) was generated by PCR mutation. Antibodies were then produced and purified with Protein A chromatography. Then, the antibody was conjugated to the cytotoxic drug vcMMAE, prior reduction with TCEP: for heterogeneous Trastuzumab-vcMMAE with an aimed DAR of 4, the drug was added immediately after the reduction step, while for homogeneous Trastuzumab-vcMMAE DAR 8, a dialysis was added between the reduction and conjugation. For Trastuzumab_cys114, after the reduction, a reoxidation step was applied; both steps followed by dialysis. The generated ADCs were analysed with HPLC-HIC/SEC, and their antiproliferative activity was determined through a MTS assay with breast cancer SKBR3 (HER2+) cells.

**Results**

Trastuzumab and Trastuzumab_cys114 were cloned and produced in HEK293, and >99% purity was achieved after purification. Trastuzumab and Trastuzumab_cys114 were conjugated to vcMMAE. The reduction with TCEP was pointed as the critical step for obtaining the desired DAR. Molar proportions TCEP:Trastuzumab of 4 and 150 allowed to obtain DARs close to 4 (heterogeneous) and 8 (homogeneous), respectively. ADC molecules with expected DARs of 2, 4 and 8 were generated and confirmed by HPLC (DARs of 1.82, 4.08, and 7.7, respectively). The recovery yield for each ADC was: 47.8, 73.5 and 40.1% for DARs of 2, 4 and 8, respectively (Table 1). The effect of the different drug load of each ADC was observed in terms of antiproliferative activity: measured by MTS, *in vitro* antiproliferative activity was directly related to the DAR, with IC_50_ values of 723, 157, and 20 pM for DARs 2, 4 and 8 (Figure 1).

**Conclusions**

In this work, we have produced Trastuzumab and Trastuzumab_cys114 variant and conjugated them to different DARs of 2, 4 and 8, using heterogeneous and homogeneous strategies. The conjugation approach had a direct impact on the yield of the conjugation process, and the reduction conditions were critical in order to obtain the ADCs with the desired DARs, showing the importance of adjusting the different parameters of the process. The antiproliferative potency of the generated ADCs correlated well with their drug load, underlining the role of the DAR in *in vitro* antiproliferative conditions.

**Acknowledgements**

This work was supported by Farmhispania SA with funding from the CDTI program (Spanish Government).

**References**

1. Liste-Calleja, Lecina, López-Repullo, Albiol, Solà, Cairó. Lactate and glucose concomitant consumption as a self-regulated pH detoxification mechanism in HEK293 cell cultures. Appl. Microbiol. Biotechnol. 2015; 99(23):9951-9960.

2. Martínez‐Monge, Albiol, Lecina, Liste‐Calleja, Miret, Solà, & Cairó. Metabolic flux balance analysis during lactate and glucose concomitant consumption in HEK293 cell cultures. Biotechnol. Bioeng. 2019; *116*(2):388-404.

3. Junutula. et al., Site-specific conjugation of a cytotoxic drug to an antibody improves the therapeutic index. Nat. Biotechnol. 2008. 26(8):925-932.

4. Román, Miret, Scalia, Casablancas, Lecina, Cairó. Enhancing heterologous protein expression and secretion in HEK293 cells by means of combination of CMV promoter and IFNα2 signal peptide. 2016. J. Biotechnol, 239:57-60.

**Table 1 (abstract P-300).** Summary of process, physicochemical and antiproliferative activity parameters of the generated ADCs

Aimed DAR	Obtained DAR	Recovery yield (%)	IC_50_ (pM)
2	1.8	47.8	723
4	4.1	73.5	157
8	7.7	40.1	20

**Fig. 1 (abstract P-300).**
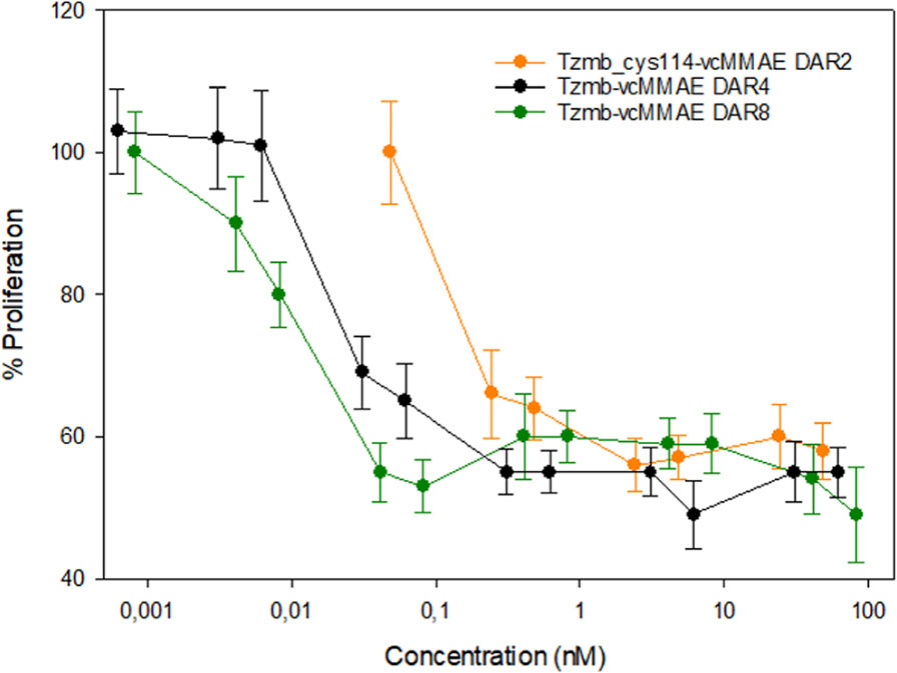
In vitro antiproliferative activity of Trastuzumab DAR 2, 4 and 8 ADCs, using HER2+ breast cancer model SKBR3 cells

### P-305 Impact of IgG and Fcγ receptors N-glycosylation upon their interaction

#### Florian Cambay^1, 2^, Olivier Henry^1^, Yves Durocher^2^, Gregory De Crescenzo^1^

##### ^1^Chemical engineering department, Ecole Polytechnique de Montréal; ^2^Human Health Therapeutics Research Center, National Research Council, Montréal, Canada

###### **Correspondence:** Florian Cambay; Gregory De Crescenzo (gregory.decrescenzo@polymtl.ca)

**Background**

The N-glycosylation profile of monoclonal antibodies (mAbs) is known to influence their pharmacodynamic and pharmacokinetic properties and is so considered as a critical quality attribute. Therapeutic agents with enhanced clinical efficacy have thus been developed via the selective enrichment of desired mAbs glycoforms. Of interest, the N-glycosylation of Fcγ receptors (FcγRs) can also affect immunoglobulin G (IgG) binding even though the mechanisms are still to be clearly determined. In this work, we fully investigated the influence of both IgG1 and FcγRs N-glycosylation upon their interaction.

**Materials and methods**

To this end, we used a surface plasmon resonance (SPR) based assay aiming at measuring the interactions between IgGs and the extracellular domain of the FcγRs immobilized on the biosensor surface with our outperforming capture strategy based on coiled-coil interactions [1]. Then, the affinity of eight well-characterized IgG1 glycoforms for FcγRs were determined while site-directed mutagenesis combined to glycoengineering were used to assess the FcγRs N-glycosylation impact on several IgG1 glycoforms binding.

**Results**

We first demonstrated that the IgG1 N-glycan profiles differently influenced the affinity for the FcγRs with an expected major effect on FcγRIIIa binding. We observed a marked impact in the absence of core fucose and a modest, yet noteworthy, impact of galactose and sialic acids regardless the fucosylation level on FcγRs binding. Moreover, affinities measured by SPR were perfectly echoed by an ADCC assay. On the other hand, both macro- and microheterogeneity of FcγRs N-glycosylation impacted to various extent their affinity for IgG glycoforms.

**Conclusions**

Altogether, our results unravelled the complex and strong influence of N-glycosylation upon the FcγRs/IgG1 binding and will be instrumental to understand the impact of FcγRs N-glycosylation in their natural forms, while helping the development of next-generation mAbs.

**Acknowledgements**

This work was supported by the National Research Council Canada: Human Health Therapeutics Research Centre (YD), the Canada Research Chair on Protein-Enhanced Biomaterials (GDC), the NSERC discovery program (GDC) and by the TransMedTech Institute (NanoBio Technology Platform) and its main funding partner, the Canada First Research Excellence Fund.

**Reference**

1. Cambay F, Henry O, Durocher Y, De Crescenzo G. Impact of N-glycosylation on Fcgamma receptor / IgG interactions: unravelling differences with an enhanced surface plasmon resonance biosensor assay based on coiled-coil interactions. MAbs. 2019; 11:435-52.

### P-309 The effect of telomere sequences on chromosomal translocations

#### Jun Ho Lee^1^, Wataru Tanaka^1^, Noriko Yamano-Adachi^1^, Yuichi Koga^1^, Takeshi Omasa^1, 2^

##### ^1^Graduate school of Engineering, Osaka University, Suita, Osaka, 560-0871, Japan; ^2^Manufacturing Technology Association of Biologics, Kobe, 650-0047, Japan

###### **Correspondence:** Jun Ho Lee (omasa@bio.eng.osaka-u.ac.jp)

**Background**

Chinese hamster ovary (CHO) cells are widely used to produce therapeutic antibodies. Construction of a stable high producer from a single CHO cell is time-consuming, and one of the bottlenecks for cell line development (CLD) is frequent chromosomal translocations. However, the loci of chromosomal translocations in CHO cells have not been identified. To control the genome stability of CHO cells, it is necessary to find the specific loci and narrow down the target protein to be engineered.

In this study, we focused on the CHO genomic BAC library-derived DNA sequence, located in every chromosome except for chromosomes 1 and 2, which are stable chromosomes with relatively low chance of chromosomal translocations [1]. The DNA sequence contains interstitial telomeric sequences (ITSs), which are telomeric (TTAGGG)n repeats found in intrachromosomal sites. The ITS is thought to be a candidate locus for chromosomal translocations and a potential cause of chromosome and recombinant gene loss [2]. Therefore, the purpose of this research is to analyze the relationship between ITSs and chromosomal translocations in Chinese hamster-derived cell lines.

**Materials and methods**

Two kinds of Chinese hamster-derived cells of different origins were used in this research. First, a CHO-DG44 derived sub-clone, CHO-DG44-SC20, with a modal chromosome number of 20 as of a standard Chinese hamster. Second, the CHL-YN cell line, obtained from Chinese hamster lung cells in our laboratory cultivated for 452 days after isolation from the Chinese hamster, was used to represent a cell line at an early stage after isolation.

Multi-color fluorescence *in situ* hybridization (mFISH) was used to observe chromosomal translocations, and standard FISH with a probe containing the telomere sequence was used to visualize ITSs. By comparing the fluorescence microscopy pictures of the stained chromatin spreads obtained from the two FISH methods, the number of chromosomal translocations and ITSs of each cell lines were analyzed and evaluated statistically.

**Results**

The FISH result showed that the number of chromosomes and ITSs in the two cell lines did not differ from each other, while the number of chromosomal translocations and ITSs at chromosomal translocation sites were greater in CHO-DG44-SC20, which has gone through a longer period of cultivation than CHL-YN (day 452) (Table 1). Also, of the chromosomal translocations, around 74% and 33% of them colocalized with ITSs in CHO-DG44-SC20 and CHL-YN, respectively (Figure 1).

**Conclusions**

In this study, we identified a clear relationship between chromosomal translocations and ITSs in Chinese hamster-derived cell lines. The higher number of chromosomal translocations in CHO-DG44-SC20 indicates that a greater number of chromosomal translocations have occurred over decades. The higher number of ITSs at chromosomal translocations shows that many of the chromosomal translocations in CHO-DG44-SC20 occurred at ITSs. The ratio of chromosomal translocations at ITSs also supports this idea. At least 30% of the chromosomal translocations for both cell lines occurred at ITSs. Therefore, our result indicates that ITSs are the loci where chromosomal translocations frequently occur in Chinese hamster-derived cells, and may be targets for improving CLD of CHO by cell engineering.

**Acknowledgements**

This research is partially supported by the developing key technologies for discovering and manufacturing pharmaceuticals used for next-generation treatments (Japan Agency for Medical Research and development (AMED) under Grant Number JP17ae0101003, JP18ae0101056, 57, 58, and 66) and MEXT/JSPS KAKENHI Grant Number JP17H06157 and JP18H05940.

**References**

1. Omasa T, Cao Y, Park JY, Takagi Y, Kimura S, Yano H, Honda K, Asakawa S, Shimizu N, Ohtake H. Bacterial artificial chromosome library for genome-wide analysis of Chinese hamster ovary cells. Biotechnol. Bioeng. 2009; 104:986-94.

2. Dancis BM, Holmquist GP. Telomere replication and fusion in eukaryotes. J. Theor. Biol. 1979; 78:211-24

**Table 1 (abstract P-309).** FISH analysis

	CHO-DG44-SC20	CHL-YN (day 452)
Chromosomes	19.9	22.1
Translocations	7.3	4.6
ITSs	13.3	14.6
ITS at translocations	5.4	1.5

**Fig. 1 (abstract P-309).**
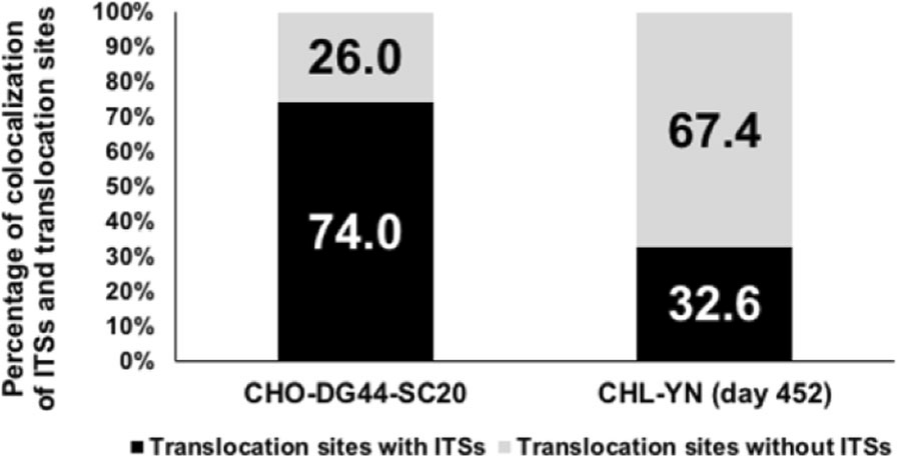
The ratio of chromosomal translocations at ITSs and non-ITSs

### P-311 A new dimeric blood-brain barrier penetrating TNFα inhibitor

#### Viana Manrique-Suárez^1^, Luis Macaya^1^, Nelson Santiago Vispo^2^ and Oliberto Sánchez^1^

##### ^1^Pharmacology department, Biology sciences faculty, Universidad de Concepción, 403000, Chile; ^2^Yachay Tech University, Urcuqui, 170522, Ecuador

###### **Correspondence:** Viana Manrique-Suárez (osanchez@udec.cl)

**Background**

In pathological conditions, microglia release a large amount of tumor necrosis factor alpha (TNFα) that plays a pathogenic role in inflammatory neurological disorders. Neutralization of TNFα and/ or blocking its binding to receptors has become a major strategy in the therapy of inflammatory diseases [1]. Among the antagonists approved by FDA are engineered monoclonal antibodies and fusion proteins containing the extracellular domain of TNFα receptors (TNFR) linked to the Fc portion of human immunoglobulin (Etanercept). The dimeric nature of these biologic inhibitors increases its affinity by 50 to 1000 folds in comparison to soluble monomeric forms [2]. However, large sizes of biological TNFα inhibitors (TNFI) impede its cross through blood-brain barrier (BBB), and therefore its therapy applications [3]. TNF receptor 2 extracellular domain (TNFR2), a TNFI, was reengineered [4] by its fusion to a BBB penetrating peptide (MTH) and the VEGF dimerization domain.

**Materials and methods**

\The Angiopeptide [5] was used to mediate transcytosis as a MTH (Molecular Trojan Horse). In silico systematic mutations on VEGF’s amino acid sequences were performed using BeAtMuSiC server. The amino acids that mediate VGF binding to its receptors KDR and FLTR-1, without affecting dimerizing properties were identified [6]. The fusion protein (**M**TH-**T**NFR2-**V**EGF*) coding sequence was cloned and expressed in stably transfected Chinese hamster ovary cells. The angiogenic activity was quantified by endothelial cell tube formation assay. Binding capacity and in vitro biological activity were determined by thermophoresis and inhibition of TNFα cytotoxicity on L929 cells assays, respectively. Immunofluorescence microscopy was used to evaluate the internalization of the recombinant protein by ECV-304 cell line.

**Results**

Two new mutations, Y21P (ΔG = 5.89Kcal/mol; 4.02 kcal/mol) and Y25G (ΔG = 5.24Kcal/mol; 5.29 kcal/mol), successfully diminish VEGF binding to both receptors, and therefore were included in the recombinant molecule. Purified MTH-TNFR2-VEGF* appeared as a glycosylated and dimeric forms even under reduced conditions. However, it wasn't observed *in vitro* significant angiogenic activity quantified by aortic ring and scratch assays. This evidence suggests no biological activity of the mutated VEGF dimerization domain. Our chimeric protein binds TNFα with the same affinity of Etanercept, without significant statistical differences in their equilibrium dissociation constant values (Kd), determined by microscale thermophoresis assay (Table 1). Furthermore, MTH-TNFR2-VEGF* exhibited similar biological activity to Etanercept by inhibiting TNFα induced cytotoxicity on L929 cells *in vitro*. Internalization of MTH-TNFR2-VEGF* in endothelial cells was also proved (Figure 1).

**Conclusions**

These results make MTH-TNFR2-VEGF* a novel anti-TNFα candidate drug of systemic administration for neurological disorder treatment. Additionally, VEGF* dimerization domain decribed would be usefull as a new strategy for the production of dimeric recombinant biomolecules.

**Acknowledgments**

This work was funded by the CONICYT PFCHA/ Beca Doctorado Nacional/ 2017- 21170539.

**References**

1. Sedger, L.M. and M.F. McDermott, TNF and TNF-receptors: From mediators of cell death and inflammation to therapeutic giants - past, present and future. Cytokine Growth Factor Rev, 2014. 25(4): p. 453-72.

2. Goffe, B. and J.C. Cather, Etanercept: An overview. J Am Acad Dermatol, 2003. 49(2 Suppl): p. S105-11.

3. Tobinick, E., Perispinal etanercept advances as a neurotherapeutic. Expert Rev Neurother, 2018. 18(6): p. 453-455.

4. Pardridge, W.M. and R.J. Boado, Reengineering biopharmaceuticals for targeted delivery across the blood-brain barrier. Methods Enzymol, 2012. 503: p. 269-92.

5. Demeule, M., et al., Involvement of the low-density lipoprotein receptor-related protein in the transcytosis of the brain delivery vector angiopep-2. J Neurochem, 2008. 106(4): p. 1534-44.

6. Keyt, B.A., et al., Identification of vascular endothelial growth factor determinants for binding KDR and FLT-1 receptors. Generation of receptor-selective VEGF variants by site-directed mutagenesis. J Biol Chem, 1996. 271(10): p. 5638-46.

**Table 1 (abstract P-311).** Kd values of Etanercept and MTH-TNFR2-VEGF* for TNFα labelled with an Alexa Fluor dye (A 647nm), based on the measured microscale thermophoresis data

Ligand	Kd
Etanercept	235±96.7nM
MTH-TNFR2-VEGF*	279±40.9nM

**Fig. 1 (abstract P-311).**
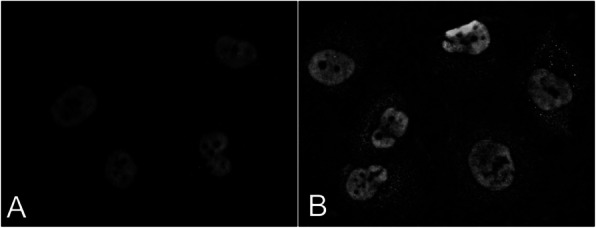
Epifluorescent microscopy of ECV-304 endothelial cells after 2 hours of incubation with **A.** PBS 1X or **B.** MTH-TNFR2-VEGF*, 250mM

### P-315 Development of Type I Allergy Therapeutics from Green Asparagus

#### Akira Iwamoto^1^, Hiroshi Hamajima^2^, Keisuke Tsuge^1^, Yumi Tsuruta^1^, Hiroaki Yotsumoto^3^, Teruyoshi Yanagita ^2,3^

##### ^1^Division of Food Industry, Industrial Technology Center of Saga, Saga city, 849-0932, Japan; ^2^Saga Food & Cosmetic Laboratory, Division of Research and Development Promotion, Saga Prefectural Regional Industry Center, Saga city, 849-0932, Japan; ^3^Development of Health and Nutrition Sciences, Nishikyushu University, Kanzaki city, 842-0015, Japan

###### **Correspondence:** Akira Iwamoto (iwamoto@saga-itc.jp)

**Background**

Type I allergic reactions, such as asthma and pollinosis, are major conditions that are common all over the world. It is well known that type I allergic reaction is provoked by the degranulation of immune granulocytes (mast cells, basophils). The bioactive components of green asparagus are thought to partly impact on these events. Although green asparagus (*Asparagus officinalis L.*) consumption has increased worldwide, these are few reports concerning its anti-allergic effects. Here, we present evidence of novel degranulation inhibitors derived from asparagus and their mechanism of action using relevant allergy models.

**Material and methods**

Anti-allergic activity of green asparagus was investigated by the calcium ionophore A23187-induced degranulation in rat basophilic leukemia cells RBL-2H3. The release of β-hexosaminidase was assessed as a marker of degranulation.

The effect of asparagus components on allergic response was evaluated by atopic dermatitis model mouse NC/Nga. Mice were orally administrated with glycolipid or phospholipid fraction derived from green asparagus, and periodically induced atopic dermatitis by picryl chloride.

**Results**

In vitro cell assay showed that the 50% EtOH extract of green asparagus markedly inhibited β-hexosaminidase release by 45% without any cytotoxicity in A23187-stimulated RBL-2H3 cells. Both glycolipid and phospholipid fractions extracted from green asparagus resulted in the decrease of β-hexosaminidase release (Figure 1). Based on in vitro cell assay, suggesting these components could be responsible for the alleviation of type I allergy. Furthermore, in vivo assay, the oral administration of glycolipid and phospholipid fractions remarkably palliated skin manifestation in both back and ears in atopic dermatitis model mice (Figure 2).

**Conclusion**

In vitro and in vivo studies suggest that glycolipid and phospholipid of green asparagus improve the atopic dermatitis through the inhibition of degranulation reaction in granulocytes.

**Fig. 1 (abstract P-315).**
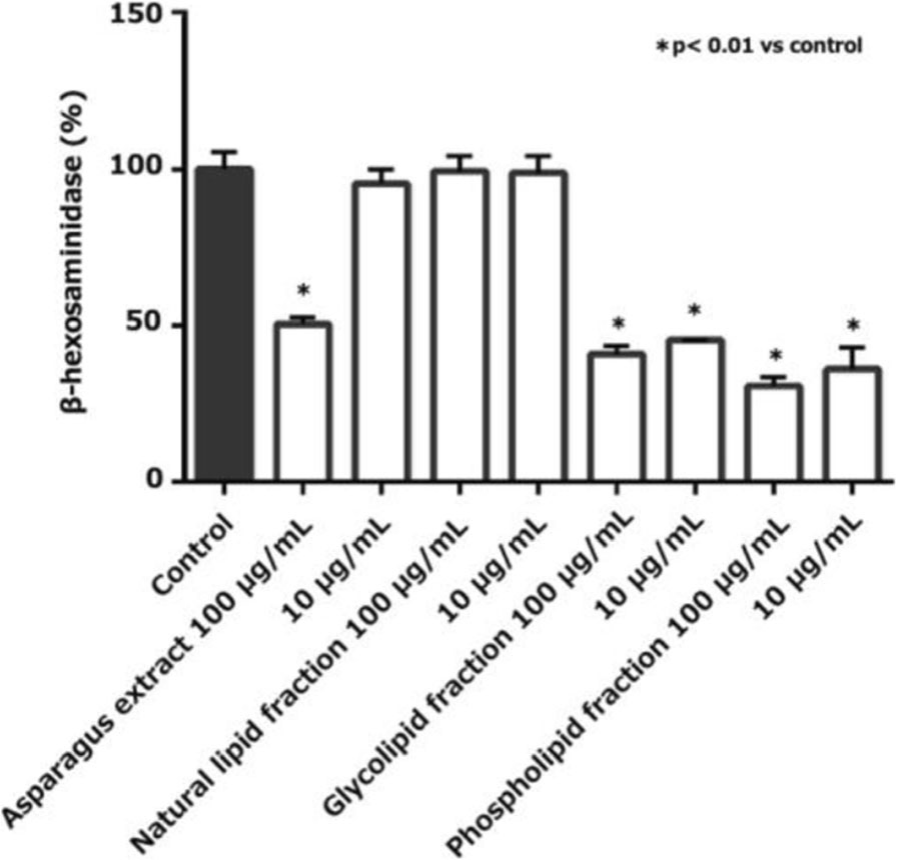
Inhibitory effect of purified fraction on the degranulation of RBL-2H3

**Fig. 2 (abstract P-315).**
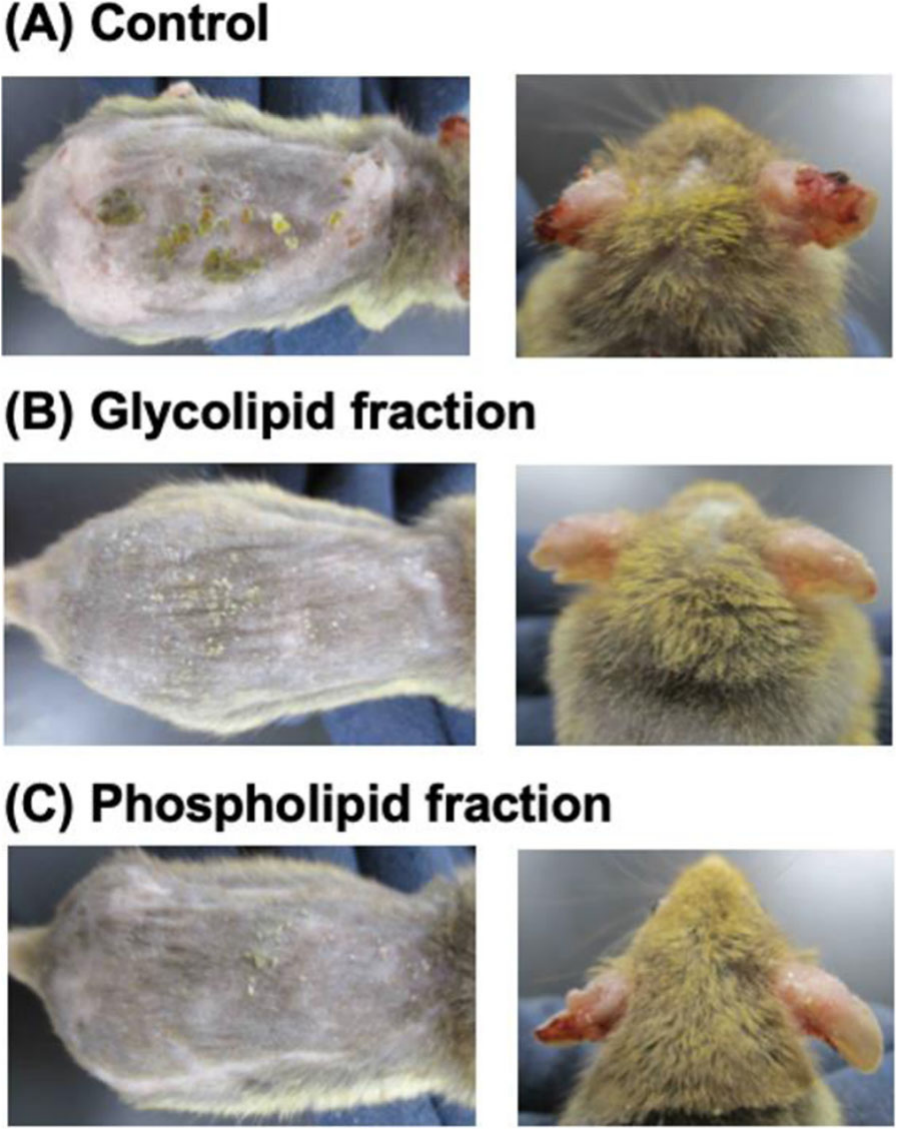
Representative clinical feature of NC/Nga mouse

### P-317 Cell culture evaluation of rhEPO neuroprotection and neuroplasticity

#### María de los Milagros Bürgi Fissolo^1^, Gabriela Aparicio^2^, Ricardo Kratje^1^, Camila Scorticati^2^, Marcos R. Oggero^1^

##### ^1^Centro Biotecnológico del Litoral, Facultad de Bioquímica y Ciencia Biológicas-Universidad Nacional del Litoral, Santa Fe, Argentina; ^2^Laboratorio de Neurobiología, IIB-UNSAM, CONICET, Buenos Aires, Argentina

###### **Correspondence:** María de los Milagros Bürgi Fissolo (moggero@fbcb.unl.edu.ar)

**Background**

Neurodegenerative diseases are incurable conditions that progressive affect nerve cells. According to the World Health Organization (WHO), neurodegenerative diseases affect millions of people around the world, mainly due to the increase in life expectancy and the concomitant increase in the world's elderly population [1]. There is not treatment for these pathologies. In this sense, human erythropoietin (hEPO) has a leading role because its antiapoptotic, antiinflammatory, and antioxidant effects have been observed in neural tissues [2, 3]. In order to evaluate the *in vitro* neuroprotection and neuroplasticity of rhEPO and putative derivatives, two cell culture-based procedures were optimized.

**Materials and methods**

The *in vitro* neuroprotective activity of different concentrations of CHO-derived rhEPO was determined as the capacity of the cytokine to reverse the staurosporine (STP)-induced apoptosis in cultures of: murine neuroblastoma cell line (N2a) and hippocampal neurons at 5 and 11 days *in vitro* (DIV). The neuroprotection was measured as the percentage of viable cells (N2a cells) or apoptotic nuclei (HPNC) after STP-induced apoptosis. Neuroplasticity was determined by measuring neuritogenesis, filopodia and synapses formation. N2a cells were incubated for 3 h with 10, 50 and 300 ng/ml of rhEPO and then, the longest neurite, the average neurite length and the number of neurites per cell were quantified using the NeuronJ plugin for ImageJ (NIH). Hippocampal neurons treated with rhEPO were used to evaluate filopodia (5 DIV) and synapses (15 DIV) formation. Briefly, the number of filopodia per 20 μm of neurite length was quantified in 40–50 neurites per group. Synapse formation was measured by colocalization of puncta, along 25 μm of dendrite length, between pre- and post-synaptic markers in 20-30 neurons per condition, using three dendritic regions per neuron. The selected neurons were at least two cell diameters away from their nearest neighbor [4]. Colocalization of puncta were determined using the plugin Puncta Analyzer of ImageJ. For statistics, significant differences were determined using one-way ANOVA followed by the Dunett’s test or two tails test, as appropriate.

**Results**

rhEPO was capable to significantly reverse the STP-induced apoptosis in a dose-response effect in both N2a cells and neurons (between p<0.01 and p<0.001). The stronger effect was observed in neurons at 11 DIV, which represents mature neurons (Figure 1).

Regarding to neuroplasticity, in the Table 1 are summarized the results as the ratio of observed treatment response vs control. N2a cells treated with 50 and 300 ng/ml of rhEPO exhibited significant increase in both neurite length (*p<0.05 and **p<0.001) and number of neurites per cell (*p<0.05 and **p<0.01). Moreover, same rhEPO concentrations increased filopodia density (*p<0.05 and **p<0.001) and the number of synapses (*p<0.01 and **p<0.01) in neurons.

**Conclusions**

N2a cell line and hippocampal neurons are useful and complementary platforms to evaluate the neuroprotective and neuroplastic actions of rhEPO. This encourages us to discover new hEPO derivatives that display both roles for the prevention and recovery of neurodegenerative disease.

**Acknowledgements**

Authors would like to thank the financial support of UNL-CAI+D 2016 (50020150100024LI) and PICT (FONCyT-ANPCYT 2015-2150).

**References**

[1] WHO, Neurological Disorders: Public Health Challenges. 2006. ISBN: 978 92 4 156336 9.

[2] Ghezzi P, Brines M. Erythropoietin as an antiapoptotic, tissue-protective cytokine. Cell Death Differ. 2004; 11 Suppl 1:S37-44.

[3] Nekoui A, Blaise G, Erythropoietin and Nonhematopoietic Effects. The American Journal of the Medical Sciences, http://dx.doi.org/10.1016/j.amjms.2016.10.009

[4] Ippolito DM., Eroglu C. Quantifying synapses: an immunocytochemistry-based assay to quantify synapse number. J Vis Exp. 2010. 16;(45). pii: 2270. doi: 10.3791/2270.

**Fig. 1 (abstract P-317).**
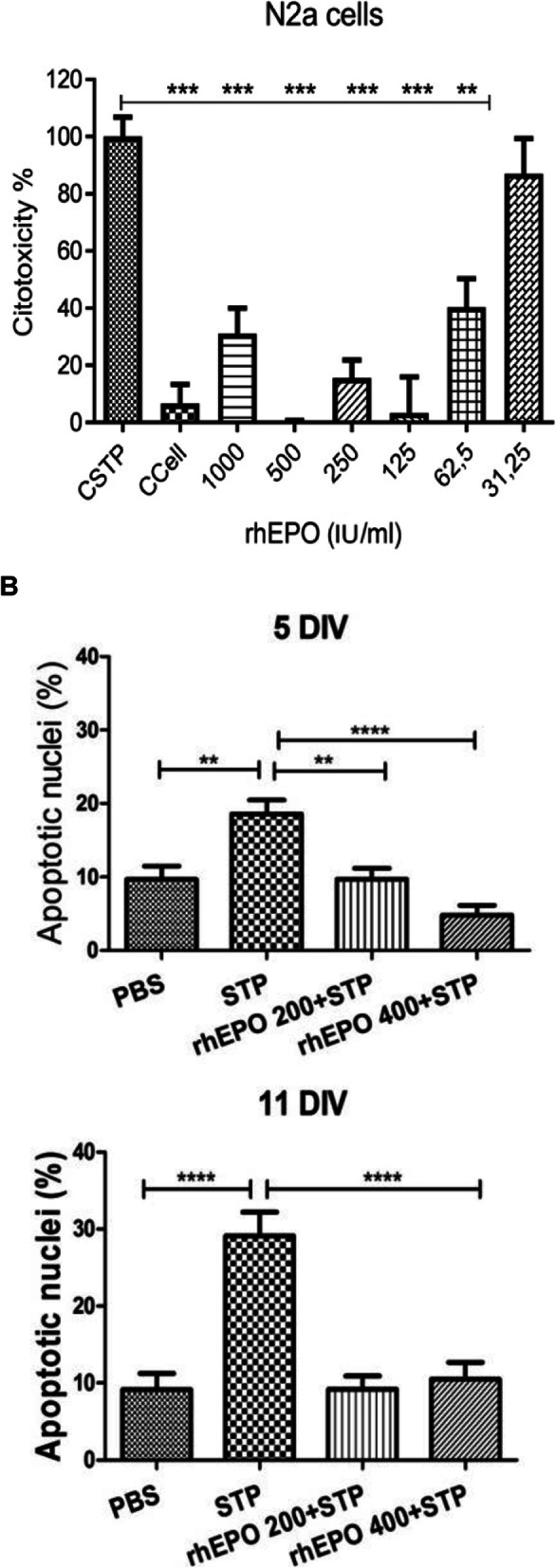
Prevention of stausporine-induced apoptosis by rhEPO in N2a cells (A) and neurons at 5 and 11DIV (B)

**Table 1 (abstract P-317).** rhEPO promotes neuroplasticity in N2a cells and hippocampal neurons

rhEPO (ng/ml)	Neuritogenesis			Filopodia	Synapses
	The longest neurite	Neurite length	Neurites per cell	Number of filopodia	Number of synapses
	1.0	1.0	1.0	1.0	1.0
**10**	1.8	1.4	2.0	1.0	1.5
**50**	2.4*	1.7	2.0*	1.4*	2.0*
**300**	3.1**	2.2	3.0**	1.6**	2.0**

### P-322 Improved *in-vitro* detection of toxic leachables in single-use material

#### Dana Lena Budde, Elke Jurkiewicz, Thorsten Adams, Gerhard Greller

##### Sartorius Stedim Biotech, Göttingen, Germany, 37079

###### **Correspondence:** Dana Lena Budde (dana.budde@sartorius-stedim.com)

**Background**

The application of single-use (SU) bioprocessing material has increased due to low cost and high process flexibility [1]. Harmful substances migrating from SU material into cell culture must be discovered, even in smallest quantities to ensure a safe drug product. Therefore, biocompatibility of SU material is evaluated according to ISO 10993-5, originally developed for medical devices, before marketing. In this study, test sensitivity of the serum dependent adherent-growing L-929 cell line, recommended in ISO 10993-5, is compared with three in-house suspension cell lines, grown in xeno-free media. In detail, biocompatibility of a PVC tubing is assessed and spiking experiments with the well-known leachable bis(2,4-di-tertbutylphenyl) phosphate (bDtBPP) [2] are carried out. Finally, the effect of serum on test sensitivity is examined.

**Material and methods**

Tubes were extracted immediately after gamma irradiation at different surface:volume ratios (S/V) in Corning® Erlenmeyer flasks. Extraction medium was cultivation medium for the respective cell line (Table 1). Samples were extracted at 36.8°C and 120 rpm. After three days, extracts were used for subsequent cell cultivation. bDtBPP (ARC Scientific, CAS: 69284-93-1) was dissolved in DMSO and further diluted in cell culture medium, ensuring a final DMSO concentration not affecting cell growth (data not shown). Cytotoxicity was evaluated by monitoring cell proliferation of HEK and CHO cells using an automated cell counter (Cedex Hires Analyzer, Roche). Viable cell density of L-929 cultures was monitored according to ISO 10993-5 [3] by the colorimetric XTT assay (AmpliChem).

**Results**

For comparison of cell sensitivity, a medical PVC tubing was extracted and extracts were cultivated (Table 1). In contrast to L-929 cells, a dose-dependent cytotoxicity was detected with CHO DG44_1 (Figure 1A). At 0.12 cm²/mL, no cell growth can be detected. In contrast, cell growth of L-929 is not impaired at any S/V indicating low sensitivity. For further examination of test sensitivity, dose-response experiments were carried out with HEK, CHO and L-929 cells in the presence of bDtBPP. The half-maximal effective concentration (EC_50_) is for both CHO and HEK cell lines by two log_10_ lower than for L-929 cells (Figure 1B). We further evaluated whether serum impacts test sensitivity. Results indicate that serum decreases cytotoxicity of bDtBPP in both HEK and CHO cells. In contrast to HEK cells, the impact of serum on CHO cells is higher (Figure 1C).

**Conclusions**

A PVC tubing that is biocompatible according to ISO 10993-5, caused drastically reduced cell growth of CHO DG4_1 cells. Results from a dose-response experiment with bDtBPP indicate that L-929 cells are much less sensitive compared to our in-house cell lines. In addition, a masking effect of serum can be assumed based on results from a spiking experiment with bDtBPP and serum. It can be concluded that sensitivity of the test system as recommended in the ISO 10993-5 is not sufficient to detect possible harmful effects of SU materials. In addition, serum masks toxic effects and thus, should be avoided in biocompatibility testing.

**Acknowledgements**

The authors thank Hanni Sun and Antje Krieter for perfect technical assistance.

**References**

1. Brecht R. Disposable Bioreactors: Maturation into Pharmaceutical Glycoprotein Manufacturing. Adv Biochem Engin/Biotechnol. 2009; 115: 1-31.

2. Hammond, M. et al. Identification of a leachable compound detrimental to cell growth in single-use bioprocess containers. PDA J Pharm Sci and Technol. 2013; 67 (2); 123-134.

3. DIN ISO 10993-5: 2009 (Appendix E), Biological evaluation of medical devices.

**Table 1 (abstract P-322).** Media and serum content used

	CHO DG44	L-929	HEK293 T
Medium	ActiCHO^1^ Seed Medium^2^	MEM (Gibco)	CD 293 (Gibco)
Serum	0%	5%	0%

^1^GE Healthcare, for CHO DG44_1

^2^Sartorius Stedim Cellca, for CHO DG44_2

**Fig. 1 (abstract P-322).**
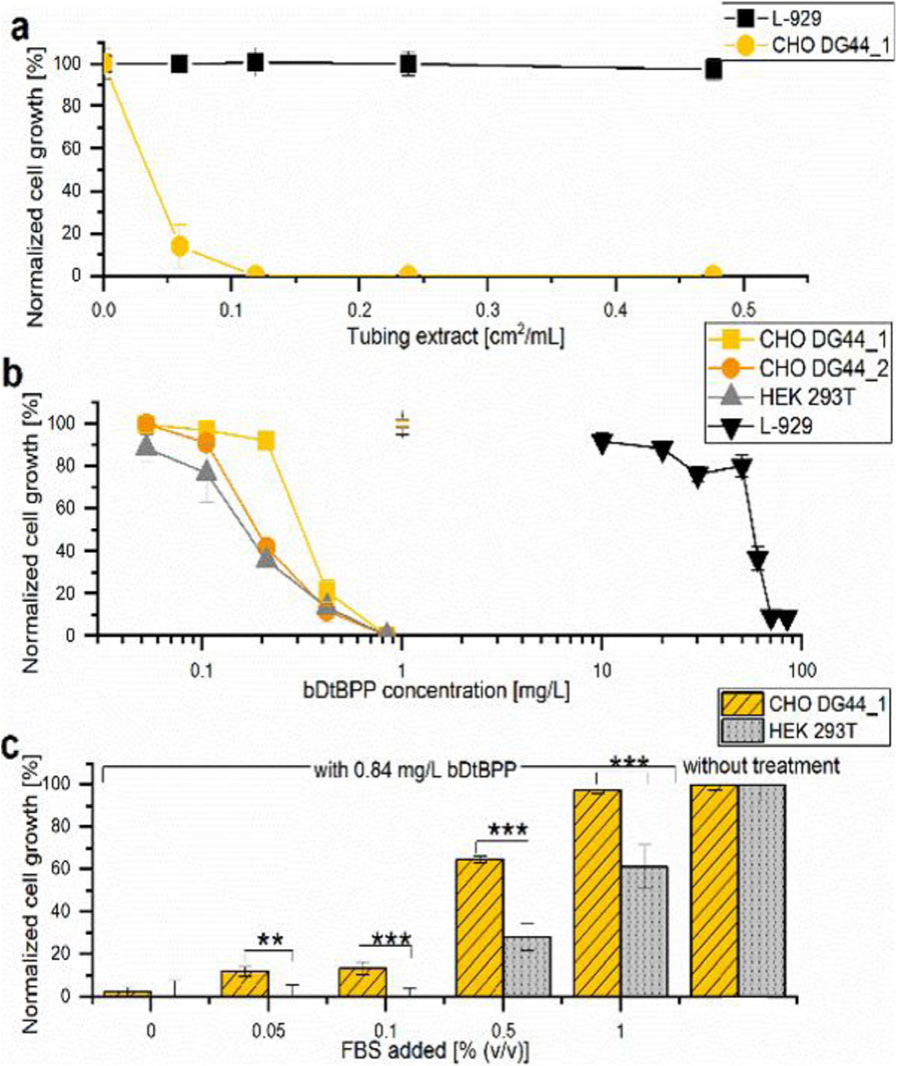
Cytotoxic effects on cell lines. **a**: Impact of tubing extracts after two (L-929, black) and three cultivation days (CHO DG44_1, yellow). **b**: Normalized cell growth of CHO DG44_1 (yellow), CHO DG44_2 (orange), L-929 (black) and HEK 293T (grey) after two (L-929) or three days of cultivation (CHO and HEK) in the presence of bDtBPP. **c**: Effect of serum on cytotoxicity of bDtBPP in CHO DG44_1 (yellow) and HEK 293T cells (grey) after three days. All tests were performed in triplicates given as mean ± standard deviation (**p<0.01, ***p<0.001, Student‘s t-test)

### P-326 Establishing fast-growing cells from Chinese hamster lung cells

#### Noriko Yamano-Adachi^1,2^, Thao Bich Nguyen^1^, Takeshi Omasa^1, 2^

##### ^1^Graduate School of Engineering, Osaka University, Osaka, Japan; ^2^Manufacturing Technology Association of Biologics, Hyogo, Japan

###### **Correspondence:** Noriko Yamano-Adachi (omasa@bio.eng.osaka-u.ac.jp)

**Background**

Chinese hamster ovary (CHO) cells are widely used as the host cells for biopharmaceutical production because of their superior post-translational ability to modify proteins [1]. However, the long doubling time (18–27±2 h) is a major drawback for these cells [2]. Therefore, we established cells with a shorter doubling time from Chinese hamster lung cells to enable the development of high-production host cells that can substitute for CHO cell lines.

**Materials and methods**

Minced lung tissues from female Chinese hamsters were placed in Iscove's Modified Dulbecco's Media (with 20% fetal bovine serum). Expanded cells were adapted to chemically defined (CD) medium by gradually decreasing the serum concentration. Cells were subjected to biosafety testing (BioReliance). An IgG1 expression vector was introduced into the cells, and the IgG1 concentration of drug-selected hetero cell pools was determined using an Octet QKe (ForteBio, CA, USA).

**Results**

A fibroblast-like morphology was evident in the primary cultured cells (Figure 1). Cells were immortalized by spontaneous transformation. The ratio of aneuploid cells increased during the immortalization process. Some translocations with the same pattern as CHO-DG44- and CHO-K1-derived cells were observed. The safety of the cell pool (named CHL-YN) was confirmed using an *in vitro* assay to detect viral contamination, sterility testing by direct inoculation, and a mycoplasma detection test.

The doubling time of CHL-YN was 10.5 h in CD medium. The cell cycle assay results suggest that the CHL-YN cells have a shorter G0/G1 cell cycle phase compared with CHO-K1 cells. The polyethylenimine-based transfection efficiency for CHL-YN exceeded 50%, the same as for CHO-K1. Between around half and almost the same amount of IgG1 was produced by CHL-YN in a shorter period compared with CHO-K1 (Table 1).

The LC-MS-based IgG1 glycan profiles displayed the same high peaks for the CHO-K1 and CHL-YN products. Selecting suitable clones and culture method optimization should assist with the development and production of new host cells.

**Conclusion**

CHL-YN cell lines are potential host cells for antibody, drug, and vaccine production. We also hope that they can be used in other applications, for example, as feeder cells.

**Acknowledgments**

This research was partially supported by the developing key technologies for discovering and manufacturing the pharmaceuticals used for next-generation treatments (Japan Agency for Medical Research and development (AMED) under Grant Nos. JP17ae0101003, JP18ae0101056, 57, 58, and 66) and MEXT/JSPS KAKENHI (Grant Nos. JP17H06157, JP18H05940). This research was also partially supported by the Cyclic Innovation for Clinical Empowerment (CiCLE) from AMED.

**References**

1. Birch JR, Onakunle Y. Biopharmaceutical Proteins: Opportunities and Challenges. Methods in Molecular Biology. 2005; 308: 1-16.

2. Xu N, Ma C, Ou J, Sun WW, Zhou L, Hu H, Liu XM. Comparative proteomic analysis of three Chinese Hamster Ovary (CHO) Host Cells. Biochemical engineering journal. 2017; 124: 122-129.

**Fig. 1 (abstract P-326).**
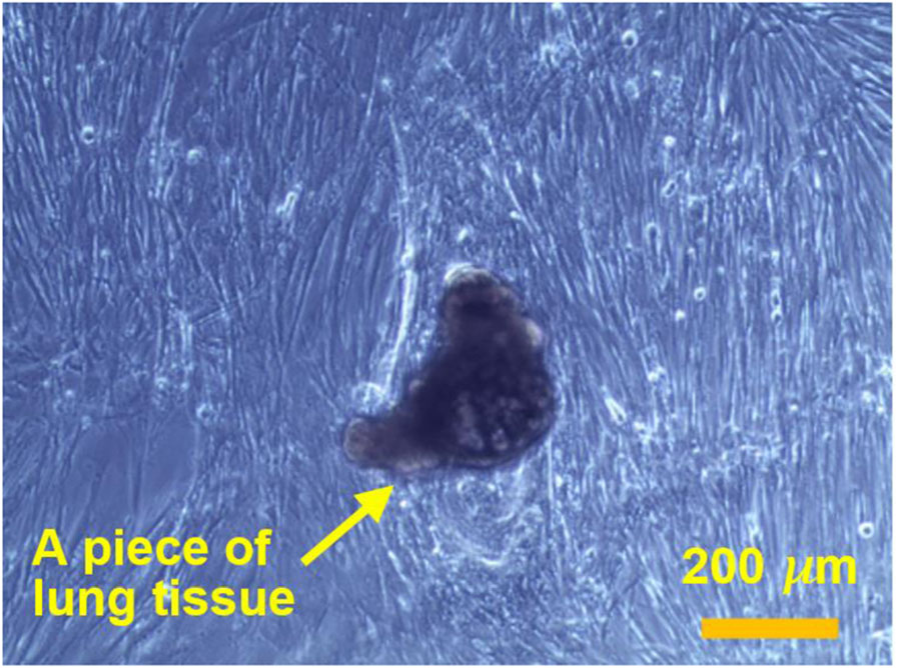
Fibroblast-like morphologies of CHL-YN cells on day 10 post-culture

**Table 1 (abstract P-326).** Productivity assessment of CHL-YN cells *vs*. CHO-K1 cells. Expression of the two IgG1 types was validated in flask-batch cultures of transfected hetero cell pools

Antibody	Medium	Cell	Specific growth rate (h^-1^)	Doubling time (h)	Specific production rate (pg cell^-1^ h^-1^)
MAB1 (IgG1)	EX-CELL CD	CHO-K1	0.035	19.8	0.51
		CHL-YN	0.062	11.1	0.86
	JX G017-MAB01	CHO-K1	0.035	19.7	0.73
		CHL-YN	0.065	10.7	1.18
MAB2 (IgG1)	EX-CELL CD	CHO-K1	0.034	20.1	0.68
		CHL-YN	0.056	12.3	0.28
	JX G017-MAB01	CHO-K1	0.034	20.6	0.71
		CHL-YN	0.050	13.9	0.46

### P-328 Scale-Up Of Clinical Grade Multipotent Mesenchymal Stromal Cells

#### Joaquim Vives^1,2,3^, Núria Marí-Buyé^4^, Alba López-Fernández^1,2^, Margarita Blanco^1^, Sílvia Torrents^1^, Clémentine Mirabel^1,2^, Paula Martínez^3^, David Horna^4^, Miquel Costa^4^, Susana G. Gómez^1^, Sergi Querol^1^

##### ^1^Servei de Teràpia Cel·lular, Banc de Sang i Teixits, Edifici Dr. Frederic Duran i Jordà, Passeig Taulat, 116, 08005 Barcelona, Spain; ^2^Musculoskeletal Tissue Engineering Group, Vall d’Hebron Research Institute (VHIR), Universitat Autònoma de Barcelona, Passeig de la Vall d’Hebron 129-139, 08035 Barcelona, Spain; ^3^Departament de Medicina, Universitat Autònoma de Barcelona, Passeig de la Vall d’Hebron 129-139, 08035 Barcelona, Spain; ^4^Aglaris Cells S.L., Calle Santiago Grisolía 2, 28760 Tres Cantos, Madrid, Spain

###### **Correspondence:** Joaquim Vives (jvives@bst.cat)

**Background**

The increasing demand of clinical-grade multipotent Mesenchymal Stromal Cells (MSC) for therapeutic use, particularly in tissue regeneration and the management of immunological disorders, has prompted developers to reconsider productive designs. In most cases, cellular therapy production is restricted to the autologous use with small batches of cells expanded in 2-dimensional (2D) planar cultures. However, the need of larger doses for use in allogeneic treatments implies the scale-up of the manufacturing process in 3-dimensional (3D) culture systems for volumetric production [1, 2]. Therefore, the scale-up leads to new challenges in the manufacture of cells for therapy that must consistently retain their critical quality attributes (CQA) from batch to batch.

Our strategies to identify suitable donors, MSC derivation from source tissue, scale-up and banking in a cost-effective manner are presented, highlighting challenges and opportunities that are not restricted to the scale up of MSC but common in the field of cell therapy manufacturing regardless of the cell type of interest.

**Materials and methods**

MSC were isolated from the Wharton’s jelly (WJ) of donated umbilical cord tissue as reported elsewhere [1, 3]. *Ex vivo* expansion of MSC was performed manually for the creation of a Master Cell Bank compatible with the Good Manufacturing Practice (GMP)-compliant production environment. Further cell culture expansion was performed manually using 5-tiered CellSTACK (Corning) for the generation of drug products used in a Phase I trial for the treatment of chronic spinal cord injury (EudraCT No. 2015-005786-23). Alternatively, WJ-MSC were expanded in bioreactors (MiniBio, Applikon) on the surface of microcarriers consisting of a polymeric core with a surface modified to enhance cell attachment and growth. The microcarriers do not require hydration, which simplifies the culture setup and their smooth surface facilitates cell harvest. In this project, microcarriers were sterilized by autoclave.

Cell adherence and culture onto microcarriers was assessed qualitatively using Hoechst staining (Invitrogen) and quantitatively by nuclei count with Nucleocounter NC-200 (Chemometec) over 10-12 days of culture [4]. Identity, purity, karyotype, multipotency and immunopotency from both planar and microcarrier-based cultures were assessed as described previously [5, 6], according to the criteria established by the International Society for Cellular Therapy [7].

**Results**

WJ-MSC were successfully expanded both in planar and microcarrier-based culture strategies maintaining CQA within specifications. Optimized harvest protocol rendered high cell recovery over 80 % even at high densities (around 250,000 cells/cm^2^). Phenotypically, all cells expressed CD105, CD73 and CD90 (≥ 95 %) and were negative for CD45 and CD31 (≥ 95 %), and the viability displayed was above 70 %. A normal karyotype was verified in all cases. Both expansion methods generated cells that maintained their trilineage potential *in vitro* into the osteogenic, chondrogenic and adipogenic lineages assessed by specific stainings. Obtained cells also retained their capacity to inhibit the proliferation of stimulated lymphocytes with a percentage of inhibition higher that 30 % in all cases (Figure 1).

**Conclusions**

Conventional production methods allowed for the derivation and large-scale expansion of WJ-MSC for clinical use, however, they are suitable for small laboratories with limited production. Microcarrier expansion using bioreactors has demonstrated the feasibility of scaling up the production of larger cell doses for banking that will be used in new treatment options with allogeneic, off-the-shelf products.

**Acknowledgements**

Spanish Cell Therapy Network (RD16/0011/0028) and Generalitat de Catalunya (2017SGR719).

**References**

1. Oliver-Vila I, Coca MI, Grau-Vorster M, Pujals-Fonts N, Caminal M, Casamayor-Genescà A, et al. Evaluation of a cell-banking strategy for the production of clinical grade mesenchymal stromal cells from Wharton’s jelly. Cytotherapy. 2016;18:25–35. doi:10.1016/j.jcyt.2015.10.001.

2. Chen AKL, Reuveny S, Oh SKW. Application of human mesenchymal and pluripotent stem cell microcarrier cultures in cellular therapy: Achievements and future direction. Biotechnology Advances. 2013;31:1032–46. doi:10.1016/j.biotechadv.2013.03.006.

3. Oliver-Vila I, Coca MI, Grau-Vorster M, Pujals-Fonts N, Caminal M, Pla A, et al. Off-the-shelf mesenchymal stromal cells derived from umbilical cord tissue. BMC Proc. 2015;9 Suppl 9:P65. doi:10.1186/1753-6561-9-s9-p65.

4. Mirabel C, Puente-Massaguer E, Del Mazo-Barbara A, Reyes B, Morton P, Gòdia F, et al. Stability enhancement of clinical grade multipotent mesenchymal stromal cell-based products. J Transl Med. 2018;16:291. doi:10.1186/s12967-018-1659-4.

5. Codinach M, Blanco M, Ortega I, Lloret M, Reales L, Coca MI, et al. Design and validation of a consistent and reproducible manufacture process for the production of clinical-grade bone marrow–derived multipotent mesenchymal stromal cells. Cytotherapy. 2016;18:1197–208. doi:10.1016/j.jcyt.2016.05.012.

6. Oliver-Vila I, Ramírez-Moncayo C, Grau-Vorster M, Marín-Gallén S, Caminal M, Vives J. Optimisation of a potency assay for the assessment of immunomodulative potential of clinical grade multipotent mesenchymal stromal cells. Cytotechnology. 2018;70:31–44. doi:10.1007/s10616-017-0186-0.

7. Dominici M, Le Blanc K, Mueller I, Slaper-Cortenbach I, Marini FC, Krause DS, et al. Minimal criteria for defining multipotent mesenchymal stromal cells. The International Society for Cellular Therapy position statement. Cytotherapy. 2006;8:315–7. doi:10.1080/14653240600855905.

**Fig. 1 (abstract P-328).**
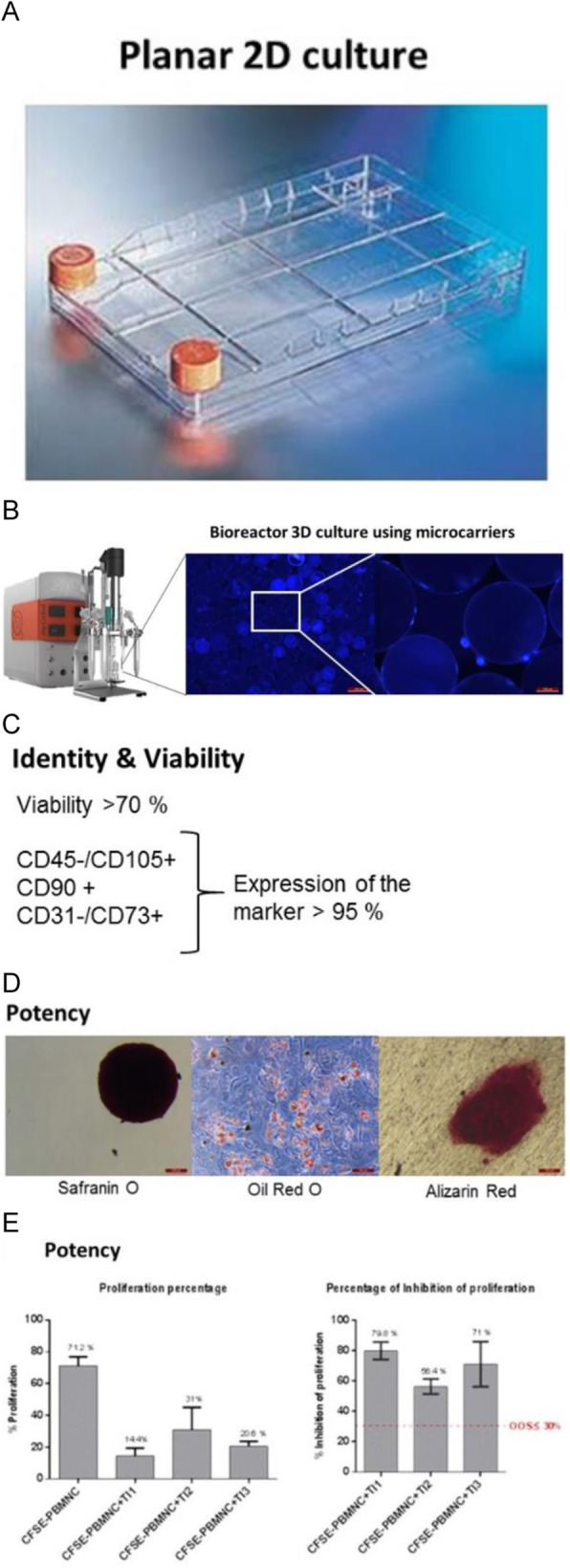
WJ-MSC characterisation from planar culture or microcarrier-based culture strategies. A) Cell culture expansion using 5-tiered CellSTACK. B) Cell culture expansion microcarriers in a bioreactor (Hoechst staining). C) Percentage of viability and percentage of surface marker for the identity of the obtained WJ-MSC. D) Representative stainings for cartilage (Safranin O), adipogenic (Oil Red O) and bone (Alizarin Red) differentiation. E) Proliferation and inhibition of proliferation percentages from immunopotency assay. TI1: test item 1; TI2: test item 2; TI3: test item 3

### P-329 Enhancing scalability and osteogenic potential of Wharton’s Jelly MSC

#### Raquel Cabrera-Pérez^1,2^, Coral García^3^, Clémentine Mirabel^1^, Marta Monguió-Tortajada^4^, Santiago Roura^5^, Francesc E. Borràs^4^, Antoni Bayes-Genis^6^, Laura Batlle-Morera^7^, Martí Lecina^3^, Joaquim Vives^1, 2^

##### ^1^Cell Therapy Service, Blood and Tissue Bank, Barcelona, 08005, Spain; ^2^Muskuloeskeletal Tissue Engineering Group, Vall d’Hebron Research Institute, Barcelona, 08035, Spain; ^3^Bioengineering Department, IQS-Ramon Llull University, Barcelona, 08017, Spain; ^4^REMAR-IVECAT Group Health Science Research Institute Germans Trias i Pujol, Badalona, 08916, Spain; ^5^ICREC Research Program, Health Science Research Institute Germans Trias i Pujol, Badalona, 08916, Spain; ^6^Cardiology Service, Germans Trias i Pujol University Hospital, Badalona, 08916, Spain; ^7^Gene Regulation, Stem Cells and Cancer, Program, Centre for Genomic Regulation (CRG), Barcelona, 08003, Spain

###### **Correspondence:** Raquel Cabrera-Pérez (jvives@bst.cat)

**Background**

The use of multipotent Mesenchymal Stromal Cells (MSC) has been reported safe and efficient for bone regeneration in increasingly prevalent bone-related conditions [1,2]. Consequently, the development of procedures based on the use of suitable allogeneic MSC that could be used off-the-shelf is absolutely necessary. Among the tissue sources for MSC isolation, Wharton’s jelly (WJ) constitutes an attractive option due to their primitive feature and their ease of isolation, which is not painful, not invasive and does not raise major ethical concerns [3]. Based on these properties, we previously reported a cell-banking strategy for the production of clinical grade WJ-MSC [4]. Nevertheless, scale-up methods are required to achieve clinically relevant cell numbers as well as determining the competence of WJ-MSC for osteogenic differentiation competences. Accordingly, we investigated the possibility of using dextran positive-charged micro carriers (Cytodex® 1) to increase WJ-MSC production, and the *in vitro* osteogenic differentiation process of WJ-MSC.

**Materials and methods**

Good Manufacturing Practice (GMP)-compliant WJ-MSC were isolated and expanded as previously reported [4,5] and MSC identity was verified to comply with the ISCT criteria [6]. To scale-up WJ-MSC production, dextran positive-charged micro carriers (Cytodex® 1, Solo Hill) were used as an alternative to traditional 2D culture systems to increase culture surface. Crystal violet staining and ATP determination were used to asses cell attachment, cell viability and growth rate. To compare WJ-MSC osteogenic commitment with that of their bone marrow (BM) MSC counterparts, cultures of both cell types were induced to differentiate *in vitro* (Stem Pro Osteogenic differentiation kit, Gibco) and alizarin red staining was performed at different time points. Gene expression profiles of osteogenic markers were assessed by quantitative real-time PCR. Finally, to evaluate the effect of BM-MSC secretome on WJ-MSC osteogenic differentiation, BM-MSC and WJ-MSC were co-cultured using 0.4 μm cell culture inserts (Falcon). To avoid contaminations between cell cultures of different origins, cells were seeded in the appropriate support (well or insert) and cultured overnight in different plates to allow cell attachment. Then, inserts were placed into the cultured wells and osteogenic differentiation was induced.

**Results**

Data from crystal violet staining and ATP content evidenced that the use of Cytodex® 1 has no impact neither on cell growth nor viability of WJ-MSC. Furthermore, although similar cell densities were obtained in 2D and Cytodex® 1 cultures, cell concentration and hence, total cell number, was 6-8 fold higher when Cytodex® 1 was used (Figure 1A). Regarding osteogenesis, alizarin red staining results and gene expression analysis revealed that WJ-MSC are less prone to differentiate into the osteogenic lineage than BM-MSC, which have been established as the first option for bone regeneration in autologous MSC-based therapies (Figure 1B). However, we found that co-culture of both cell types in transwells, which allow intercellular communication independent of cell-to-cell contact, promotes WJ-MSC osteogenic differentiation (Table 1). This suggests that secretome of BM-MSC stimulates WJ-MSC osteogenesis and that *in vivo* injection of WJ-MSC in bone microenvironment could induce WJ-MSC osteogenic differentiation.

**Conclusions**

Although further results are required before clinical translation, the use of allogeneic WJ-MSC offers many advantages in the orthopaedic field compared to other cell types.

**Acknowledgements**

Spanish Cell Therapy Network (RD16/0011/0028) and Generalitat de Catalunya (2017-SGR-719).

**References**

1. Naji A, Eitoku M, Favier B, et al. Biological functions of mesenchymal stem cells and clinical implications. Cell. Mol. Life Sci. 2019;doi: 10.1007/s00018-019-03125-1.

2. Gómez-Barrena E, Rosset P, Gebhard F, et al. Feasibility and safety of treating non-unions in tibia, femur and humerus with autologous, expanded, bone marrow-derived mesenchymal stromal cells associated with biphasic calcium phosphate biomaterials in a multicentric, non-comparative trial. Biomaterials. 2019; 196:100-108.

3. Davies JE, Walker JT, Keating A. Concise review: Wharton’s Jelly: the rich but enigmatic source of Mesenchymal Stromal Cells. Stem Cells Transl Med. 2017;6(7):1620-1630.

4. Oliver-Vila I, Coca MI, Grau-Vorster M, et al. Evaluation of a cell-banking strategy for the production of clinical grade mesenchymal stromal cells from Wharton's jelly. Cytotherapy. 2016;18(1):25-35.

5. Oliver-Vila I, Coca MI, Grau-Vorster M, et al. Off-the-shelf mesenchymal stromal cells derived from umbilical cord tissue. BMC Proc. 2015;9 Suppl 9:P65.

6. Dominici M, Le Blanc K, Mueller I, et al. Minimal criteria for defining multipotent mesenchymal stromal cells. The International Society for Cellular Therapy position statement. Cytotherapy. 2006;8:315–7.

**Fig. 1 (abstract P-329).**
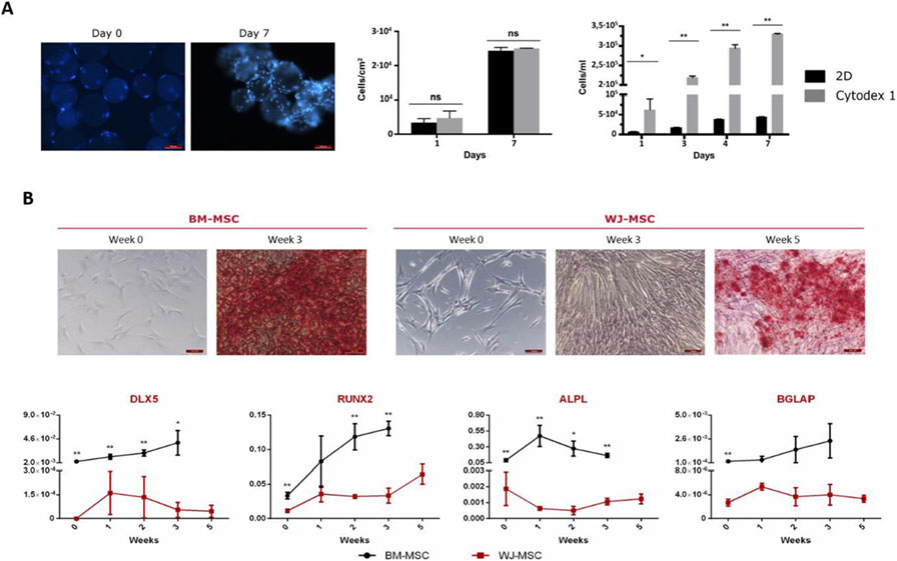
Scale-up and osteogenic differentiation of WJ-MSC. (A) Crystal violet staining and ATP determination of WJ-MSC cultured in Cytodex® 1 and 2D plates. Bars represent mean ± SD. * p<0.05, ** p<0.01 (T-test). (B) Alizarin red staining of BM-MSC and WJ-MSC cultures and expression profiles of osteogenic markers relative to GAPDH expression in WJ-MSC along osteogenic differentiation. Bars represent mean expression of three different cell lines ± SD. * p<0.05, ** p<0.01 (Multiple t-test)

**Table 1 (abstract P-329).** Results of alizarin red staining showing the effect of BM-MSC secretome on WJ-MSC osteogenesis after 3 weeks of culture. +/- indicates positive/negative staining, respectively

Insert	BM +	BM +	WJ +	WJ -
Well	BM +	WJ +	BM +	WJ -

### P-331 A Novel Single-Use Bioreactor System for Expansion of Human Mesenchymal Stem/Stromal Cells

#### David Splan^1^, Grishma Patel^1^, Janani Ravindhar^1^, Kaitlynn Bayne^1^, Alexandria Spruiel^1^, Joe Capone^2^, Tariq Haq^2^, Mark Szczypka^1^

##### ^1^Pall Biotech, 4370 Varsity Drive, Suite B. Ann Arbor, Michigan 48108, USA; ^2^Pall Biotech, 20 Walkup Drive, Westborough, Massachusetts 01581, USA

###### **Correspondence:** David Splan

**Background**

There is a significant need for efficient systems that can be used to generate primary cells and stem cells that can be readily implemented in research laboratories to expedite process development studies and pre-clinical and clinical testing. Although various platforms for expansion of sufficient cell numbers exist, these systems are rarely comprehensive enough to allow for rapid implementation by researchers. These systems are also neither easy to use nor are they robust enough to reproducibly supply high quality cells. A complete system for the generation of sufficient cell numbers for testing that utilizes a bioreactor platform which is robust and easy-to-use is therefore highly desirable.

We have previously demonstrated efficient expansion of human mesenchymal stem/stromal cells (hMSC) in the PadReactor® system single-use bioreactor to the 40 L scale. This system utilized commercially-available, well-characterized cells, specially-formulated growth media and supplements, microcarriers and a single-use bioreactor platform that could be used to generate tens of billions of cells in 7 days of culture. Here we extend these finding by employing these components in a novel bioreactor type which contains a bottom-mounted impellor. The Allegro™ STR bioreactor is a new stirred tank, single-use bioreactor platform which is scalable, compact, ergonomic and designed to maximize usability and process assurance.

**Materials and Methods**

hMSC were thawed from liquid nitrogen storage and expanded initially on cell factories for a period of 3 days. The cells were then harvested and seeded onto SoloHill microcarriers. The microcarriers were transferred into the Allegro STR50 bioreactor via the Allegro Microcarrier Delivery System (AMDS) which provides an efficient method for rapid delivery of microcarriers into bioreactors in a sterile fashion. Micorcarriers were acclimated in the bioreactor for one hour and then cells were seeded onto microcarriers at a density of 3000 cells/cm^2^ in 30 L of media. Cells were then cultured for 6 days following a fed-batch process. Samples were collected throughout the culture period to evaluate cell growth, viability, nutrient consumption and metabolite production.

At the end of the culture, cells were harvested from microcarriers almost entirely by a single operator and it was determined if critical quality attributes were retained using standard characterization assays. Bioreactor performance was compared to results achieved in the PadReactor bioreactor.

**Results**

This novel, fed-batch, microcarrier-based bioreactor process yielded excellent results. Cells were successfully propagated on microcarriers in the Allegro STR single-use bioreactor over the course of a six day culture period. Cells harvested from microcarriers at the end of the culture reached a calculated cell concentration of 0.79 B cells/L and were 97% viable. A total of 22 B cells were harvested from the culture in two hours by a single operator with minimal assistance from a second operator. Importantly, the cells propagated in this bioreactor on microcarriers retained critical quality attributes after harvest when examined in standard cell characterization assays. Results achieved in this study were also comparable to those obtained previously in the PadReactor platform.

**Conclusions**

In this study we demonstrated utilization of a single-use platform for efficient generation of high quality cells for process development studies. This system provides a practical and efficient manufacturing platform for the expansion of adherent cells for a variety of purposes.

**Acknowledgements**

We appreciate the support provided by Pall’s bioreactor applications scientists and a special thank you to Todd Lundeen for his considerable dedication.

**Fig. 1 (abstract P-331).**
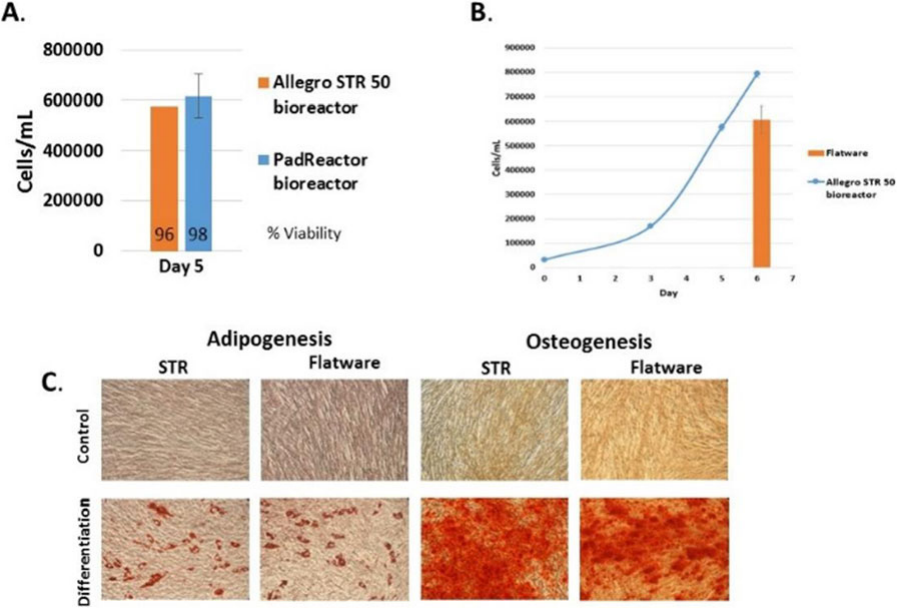
(A) hMSC yield from the Allegro STR 50 bioractor at day 5 is comparable to the PadReactor bioreactor results. Viability of harvested cells from both bioreactors was excellent and comparable. (B) The cell expansion process yielded 0.79 B cells/L in 6 days, compared to 0.61 B cells from flatware. A total of 22 B cells were obtained from the STR 50 after harvest. (C) Cell characteristics are retained when hMSCs are expanded on SoloHill microcarriers in the Allegro STR 50 bioreactor. The cells maintained differentiative potential, demonstrating adipogenic and osteogenic differentiation capacity

### P-341 Development of an anti-HER2 phage to monitor untargeted cell pannings

#### Maria R Moita^1,2^, Daniel Simão^1^, Gabriela Silva^1^, Hugo R Soares^1^, Catarina Brito^1,2^, Rene Hoet^3,4^, Ana Barbas^1,5^

##### ^1^iBET - Instituto de Biologia Experimental e Tecnológica, 2780-901 Oeiras, Portugal; ^2^Instituto de Tecnologia Química e Biológica António Xavier, Universidade Nova de Lisboa, 2780-157, Oeiras, Portugal; ^3^Department of Pathology Maastricht University, 6229 ER, Maastricht, Netherlands; ^4^Imcheck Therapeutics, Marseille, France; ^5^Bayer Portugal, 2790-012, Carnaxide, Portugal

###### **Correspondence:** Maria R Moita (ab@ibet.pt)

**Background**

Phage display technology has been successfully used in antibody discovery[1]. The methodology allows identification of specific antibody fragments to virtually any target, by an affinity selection process called panning[2]. Targeted strategies with well-established protocols have been dominating the field[3]. However, for untargeted approaches, aiming at the identification of potentially new antibodies from cells or tissues, few successful cases have been reported, highlighting the need to develop new methods and tools for this purpose[4]. Here, we describe the development and validation of a phage particle specific to the human epidermal growth factor receptor 2 (HER2) that allows monitoring the efficiency of cell panning processes.

**Materials and methods**

**Anti-HER2 Phagemid construction**: Trastuzumab single-chain variable fragment (scFv) sequence was cloned in the pIT2 parental vector from Tomlinson I+J scFv library (MRC, HGMP Resource Centre) via *NcoI* and *NotI* restriction sites (Figure 1A). The final phagemid was electroporated into TG1 cells and packaged into phage particles by infection with M13K07 helper phage. Phage specificity was evaluated by flow cytometry analysis on HER2-positive (BT474, HCC1954) and HER2-negative (HCC1806, MCF-7) cell lines using an anti-M13-PE secondary antibody (RL-ph1 clone, SCBT sc-53004).

**Cell panning:** The Tomlinson J library (1x10^12^ phages) was spiked-in with 1x10^8^ anti-HER2 phage particles (spiking 1:10.000) and blocked with FBS and Milk. Selections were performed on previously blocked 1x10^6^-1 x10^7^ cells for 1 hour, followed by washing, elution with triethylamine (TEA) and neutralization (Tris-HCl). Selection efficiency was assessed by monoclonal phage-ELISA, using a human recombinant HER2 protein (abcam, ab168896) for coating and an anti-M13-HRP antibody for detection (Sino Biological, 11973-MM05T-H). In addition, qPCR, using phage DNA extracted directly from phage output and primers specific for trastuzumab scFv sequence (Table 1) was performed.

**Results**

To generate a tool for optimization of whole cell pannings an anti-HER2 phagemid, containing the scFv sequence of Trastuzumab, a validated antibody in clinics, was constructed. Specificity was confirmed by flowytometry analysis on HER2-positive and HER2-negative breast cancer cell lines (Figure 1B). Indeed, the number of anti-HER2 phage bound-positive cells reflected the HER2 phenotype described for these cell lines.

To assess the feasibility of using this phage for optimization of cell panning protocols, positive and negative selections were performed in parallel (on BT474 and HCC1806, respectively); using the phage library spiked-in with the Anti-HER2 phage. To determine the number of anti-HER2 phage particles along the process, a qPCR method directed against the cloned scFv sequence was set up. Throughout 4 rounds of panning on HCC1806 (HER2-negative), the copy number of the phagemid decreased from 1x10^8^ to 7x10^6^ copies, while for BT474 (HER2-positive) the copy number increased from 1x10^8^ to 1x10^12^ (Figure 1C). Results from the human HER2 Phage-ELISA reassured the increasing number of anti-HER2 specific phages throughout the rounds for positive selection and no increase across the negative selection (Figure 1D).The same tendency was maintained using both methods, the differences could be related to the fact that qPCR relies on copy number and phage ELISA is influenced by phage expression levels. Overall, the results show a specific enrichment of the anti-HER2 phage through panning rounds only in HER2-expressing cells, demonstrating the potential of exploiting this phage as a tool for monitoring the performance of whole cell panning, through spike-in experiments.

**Conclusions**

We developed a system combining an anti-HER2 specific phage and analytic methods (qPCR and ELISA) that has the potential to support the optimization of whole cell untargeted selections from diverse cell sources, including spheroids and tumor tissues.

**Acknowledgements**

MRM was supported by the FCT fellowship PD/BD/128215/2016. iNOVA4Health – UID/Multi/04462/2019, a program financially supported by FCT-MES, through national funds and co-funded by FEDER under the PT2020 Partnership Agreement.

**References**

1. A. R. M. Bradbury, S. Sidhu, S. Dübel, and J. McCafferty, “Beyond natural antibodies: The power of in vitro display technologies,” Nat. Biotechnol., vol. 29, no. 3, pp. 245–254, 2011.

2. M. Sioud, “Phage Display Libraries: From Binders to Targeted Drug Delivery and Human Therapeutics,” Mol. Biotechnol., vol. 61, no. 4, pp. 286–303, 2019.

3. R. R. Minter, A. M. Sandercock, and S. J. Rust, “Phenotypic screening—the fast track to novel antibody discovery,” Drug Discov. Today Technol., vol. 23, pp. 83–90, 2017.

4. D. Sánchez-Martín et al., “Selection strategies for anticancer antibody discovery: Searching off the beaten path,” Trends Biotechnol., vol. 33, no. 5, pp. 292–301, 2015.

5. S. Goletz et al., “Selection of large diversities of antiidiotypic antibody fragments by phage display,” J. Mol. Biol., vol. 315, no. 5, pp. 1087–1097, 2002.

**Table 1 (abstract P-341).** Primer sequences for trastuzumab scFv

	Sequence
Forward	5’- TCTATCCGACCAACGGCTAC -3’
Reverse	5’- GGTACCTTGGCCCCAATAAT -3’

**Fig. 1 (abstract P-341).**
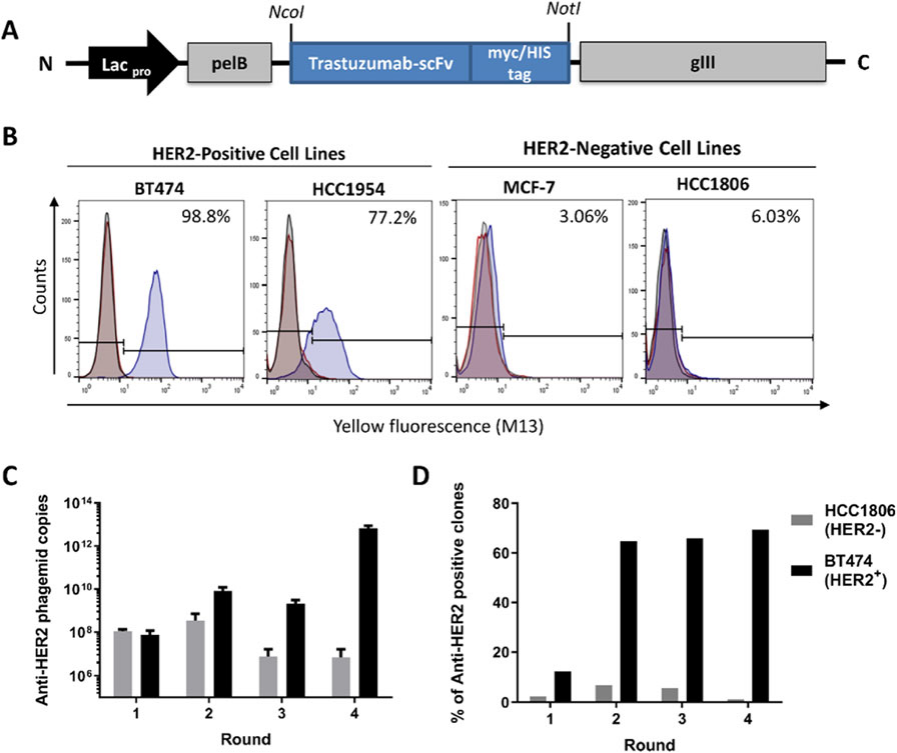
(A) Schematic diagram of the pIT2-derived Anti-HER2 phagemid expression cassette. Trastuzumab scFv is cloned under the transcriptional control of lactose promoter (Lacpro), the sequence is in-frame with the gene encoding pIII phage coat protein (gIII); the vector contains a leader sequence (pelB) and a myc/His6tag. The cloning sites used are indicated in italics. (B) Flow Cytometry analysis of phage binding to HER2-positive cells (BT474, HCC1954) and HER2-negative cells (MCF-7, HCC1806). Cells were incubated with 1x1012 phage particles for 1 hour with Anti-HER2 phages (blue), non-relevant phages (red). Phage binding was detected by a mouse Anti-M13 antibody. Control staining shown in grey. Increase in fluorescence indicates binding of Anti-HER2 phage to cells. (C) Phagemid quantification by qPCR in each round of panning using primers specific to the trastuzumab scFv sequence. Phage DNA was isolated directly from 1/5 of the output phages and the number of phagemid copies was measured with the SYBR Green Master Mix I. Number of copies was estimated using a calibration with 108 – 102 Anti-HER2 phage particles. (D) Monoclonal phage-ELISA of 4 rounds of panning using the human HER2 protein. The graph shows an increase in percentage of clones with affinity to the human HER2 protein after each round of selection on BT474 (black bars), while for HCC1806 (grey bars) the percentage is always lower than 10%

### P-408 Predicting Industrial Cell Culture Seed Trains - A Bayesian Approach

#### Tanja Hernández Rodríguez^1^, Christoph Posch^2^, Julia Schmutzhard^2^, Josef Stettner^2^, Claus Weihs^3^, Ralf Pörtner^4^, Björn Frahm^1^

##### ^1^Biotechnology & Bioprocess Engineering, Ostwestfalen-Lippe University of Applied Sciences and Arts, Lemgo, Germany; ^2^Novartis Technical Research & Development, Sandoz GmbH, Langkampfen, Austria; ^3^Faculty of Statistics, TU Dortmund University, Dortmund; ^4^Institute of Bioprocess and Biosystems Engineering, Hamburg University of Technology, Hamburg, Germany

###### **Correspondence:** Tanja Hernández Rodríguez (bjoern.frahm@th-owl.de)

**Background**

For the production of biopharmaceuticals in suspension cell culture, seed trains are required to increase the cell number from cell thawing up to production scale. Since they are time- and cost-intensive and have a significant impact on cell performance in production scale, development of optimization strategies as well as advanced seed train design & monitoring are important. Model-assisted prediction methods can be powerful tools as long as a sufficient prediction accuracy can be achieved. This can be challenging, especially when only few high-quality data are available.

This contribution illustrates the application of a Bayesian approach for seed train prediction to an industrial cell culture process, enabling inclusion and quantification of prior process knowledge and description of inference uncertainty, providing, in addition to a ‘best fit’-prediction, information about the probable deviation in form of prediction intervals.

In order to integrate information from new data for updating the prediction of the current seed train cultivation, the Bayesian updating method was applied and the impact on prediction accuracy was investigated.

**Methods**

The proposed framework, illustrated in figure 1 A, contains the following components: **a)** Prior knowledge (e.g. from literature or first training data) including uncertainty in model parameters and uncertainty of initial concentrations is described by probability distributions. **b)** Cultivation data from a production process (e.g. from small shake flask scales) and **c)** a mechanistic model have to be provided (here a model similar to Frahm et al. [1] was used).

A Bayesian approach, having the Bayes Theorem as a key element, is applied in order to propagate uncertainties, leading to posterior (after having seen the data) probability distributions of model parameters. Implementation was performed using an adaptive single component Markov Chain Monte Carlo (MCMC) algorithm [2].

The obtained posterior parameter distributions (posterior knowledge) including the maximum a posteriori (MAP) estimate (= vector of most probable model parameter values) were used for predictions including predictive uncertainty of the interesting quantities. In order to integrate information from additional data **d)** (e.g. from bioreactor scales) for updating the prediction of the current seed train cultivation, the Bayesian updating method was applied for re-estimation of model parameters, leading to updated posterior knowledge. This step can be performed several times, whenever additional data are collected.

**Results**

Figure 1 B shows the prediction of the bioreactor part (R1, R2 & R3) of an exemplary seed train for viable cell density (X_v_). Prediction based on the MAP estimate is illustrated in red, the corresponding prediction band (90%) in blue and the measurements (which were not provided during prediction) in black.

Prediction performance based on information from shake flask experiments: Prediction only based on small flask scale data (Figure 1 B i)) lead to a high prediction accuracy (99% of measurements within 90%-prediction band; relative error between predicted values and measurements = 12%). The predictive uncertainty, represented by the relative half bandwidth of the prediction band, is 77%.

Prediction performance after integration of seed train data at bioreactor scales: After performing Bayesian updating, taking further seed train data from bioreactor scales into account (Figure 1 B ii)) and updating after each reactor scale (Figure 1 B iii) and iv)), a reduction of predictive uncertainty to 18% relative half bandwidth has been achieved without loss of prediction accuracy (100% of all measurements fall within the 90%-prediction band; relative error < 6%).

**Conclusions**

Bayesian parameter estimation and Bayesian updating via the MCMC method in combination with a mechanistic model, turned out to be a suitable statistical method for seed train prediction, providing the capability of quantifying prediction uncertainty. It includes available prior knowledge and takes new data into account.

**References**

1. Frahm B. Seed train optimization for cell culture. Chapter, *Animal Cell Biotechnology-Methods and Protocols*, 3^rd^ edition, edited by Pörtner R., Springer/Humana Press, ISBN 978-1-62703-733-4, 2014.

2. Gilks W. R., Richardson S., Spiegelhalter D. J. Markov Chain Monte Carlo in practice. Boca Raton, FL: Chapman & Hall/CRC, ISBN 978-0412055515, 1998.

**Fig. 1 (abstract P-408).**
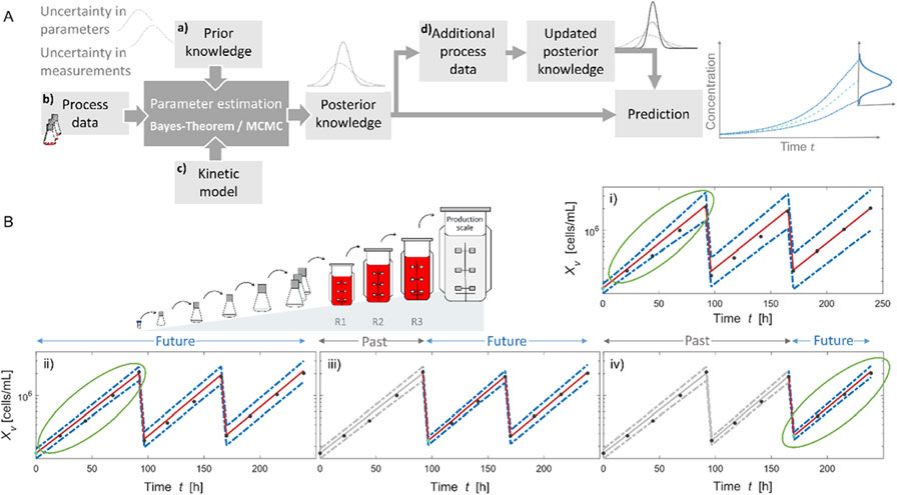
A Scheme of the Bayesian approach for parameter estimation and prediction; B Prediction of an exemplary seed train (R1, R2 & R3) for the viable cell density (Xv) at different points in time during cultivation and based on updated posterior parameter distributions. i) at t = 0 hours, based on shake flask data; ii) at t = 0 hours, based on further bioreactor data; iii) at t = 96 hours, based on further bioreactor data and the previous reactor scale and iv) at t = 170 hours, based on further bioreactor data and the previous reactor scale

### P-410 Chemometrics to monitor CHO cell cultures quality by *in situ* NIRS

#### Daniel Arturo Zavala-Ortiz^1,2^, Bruno Ebel^1^, Meng-Yao Li^1^, Maria Guadalupe Aguilar-Uscanga^2^, Javier Gomez-Rodriguez^2^, Dulce Maria Barradas-Dermitz^2^, Patricia Margaret Hayward-Jones^2^, Annie Marc^1^, Emmanuel Guedon^1^

##### ^1^Laboratoire Réactions et Génie des Procédés, CNRS-Université de Lorraine, Vandœuvre-lès-Nancy, France ; ^2^Laboratorio de Bioingeniería, Instituto Tecnológico de Veracruz, México

###### **Correspondence:** Daniel Arturo Zavala-Ortiz (bruno.ebel@univ-lorraine.fr)

**Background**

Biologicals, as monoclonal antibodies (mAb), are quite sensible to manufacturing process changes. Thus, strict quality systems, such as Quality by Design (QbD), are required to limit biological heterogeneities and then ensure patient safety. Glycosylation is the biggest source of mAb heterogeneity and its real-time control is desirable within cell cultures. However, until now it has mainly been studied through off-line approaches [1]. In this work, we compare the performance of *in situ* Near Infra-Red Spectroscopy (NIRS) coupled to different chemometric models to real-time and accurately monitor mAb quality in terms of glycosylation and saccharide patterns.

**Materials and methods**

The genetically modified DG44-M250-9 CHO cell line producing a human anti-Rhesus D mAb, was cultivated in a protein-free medium mixture consisting of PF-CHO (HyClone) and CD-CHO (Thermo Fisher Scientific) in a 1:1 volume ratio, supplemented with 4 mM glutamine. Six cell cultures were performed in 2 L benchtop bioreactors (3 batch, 2 feed-harvest, 1 batch with glucose spiking) to obtain off-line measurements. Cultures were controlled at 37°C, 50% dissolved oxygen, pH 7.2 and 90 rpm. Off-line measurement of mAb glycoforms was carried out as reported elsewhere [2]. *In situ* spectral scanning of culture media was carried out using a NIR probe with 1 mm path length, connected to the Antaris II spectrometer (Thermo Fisher Scientific).

For the generation of calibration models, spectra and off-line concentrations of total mAb, non-glycosylated mAb (NG-mAb), glycoforms containing any galactose, fucose and sialic acid as well as high mannose, were related with spectra by Partial Least Squares (PLSR), Locally Weighted Regression (LWR) and Supported Vector Regression (SVR).

**Results**

The calibration methods were firstly evaluated to monitor the presence of glycosylation in the mAb. Non-linear relationships between spectra and mAb concentrations limited the widely used PLSR, particularly for NG-mAb. Then, we addressed them by the novel use of LWR and SVR. Analysis of models showed that non-linearity was due to scattering and inherent non-linear relationship of mAb concentration with spectra. In this context, LWR was only useful to handle non-linearity by scattering, while SVR also properly managed inherent nonlinear relationship between spectra and NG-mAb concentration (Figure 1-a). Then SVR was successfully used to real time monitor concentration of mAb glycoforms containing sugar moieties conferring clinical properties (Figure 1-b).

**Conclusions**

Results clearly demonstrated that *in situ* NIR prediction models used to estimate the relationships between spectra and mAb quality criteria can be greatly improved by using the SVR regression, a chemometric method newly used in animal cell culture processes. It allows monitoring more accurately not only mAb concentration but also mAb glycosylation. These results are the starting point to go further in PAT retro-control systems so that the impact of cell culture parameters on mAb glycosylation during CHO cell culture processes may be better understood and controlled.

**Acknowledgements**

The authors acknowledge CONACyT and Campus France which granted Daniel. A. Zavala-Ortiz, and the French National Agency for Research (ANR) for supporting the ProCell-In-Line project.

**References**

1. Li B, Raya BH, Leister KJ. Ryder A.G. Performance monitoring of a mammalian cell based bioprocess using Raman spectroscopy. Anal Chim Acta. 2013; 796:84-91.

2. Li M, Ebel B, Paris C, Chauchard F, Guedon E, Marc A. Real-time monitoring of antibody glycosylation site occupancy by *in situ* Raman spectroscopy during bioreactor CHO cell cultures. Biotechnol Prog. 2018; 34(2):486–493.

**Fig. 1 (abstract P-410).**
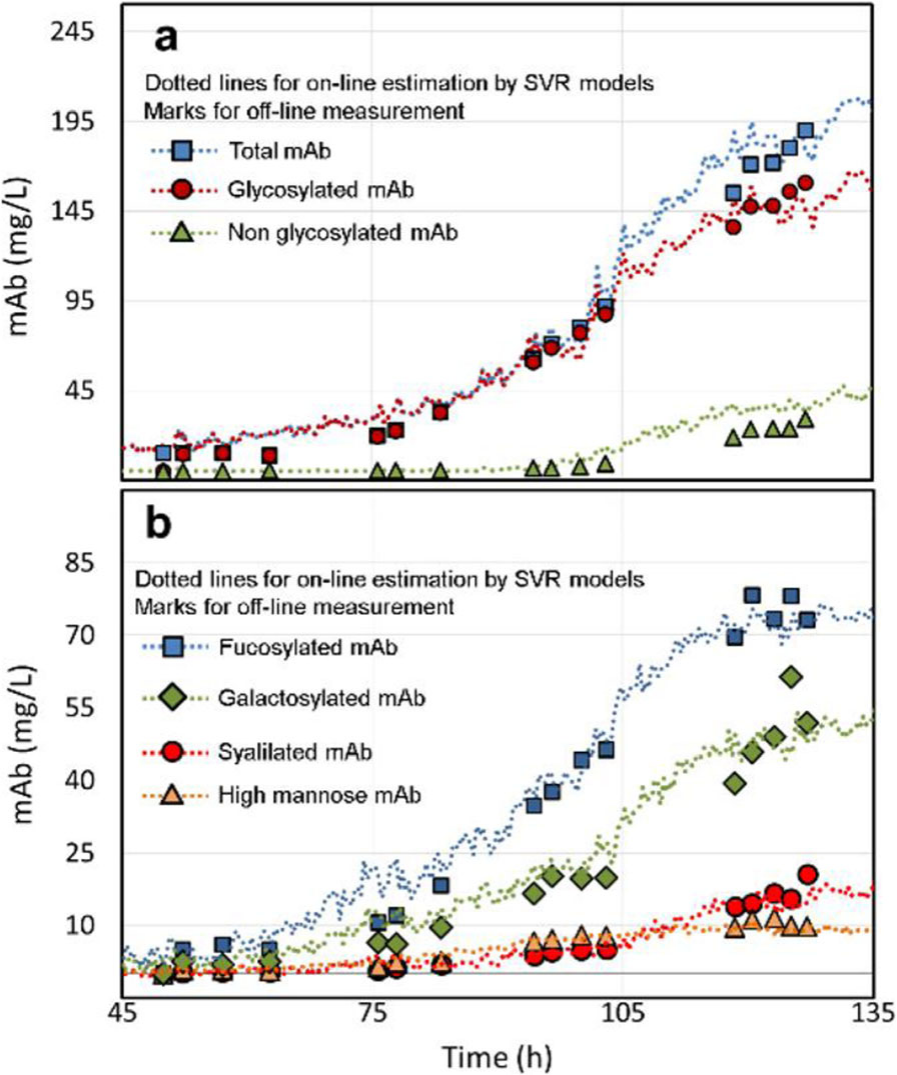
Performance of NIR SVR models to monitor mAb glycosylation

### P-423 Evaluation of Process Related Impurities impact on the determination of harvest process yield

#### Jonathan Stern, Katja Rüger, Laetitia Malphettes

##### UCB Pharma S.A., Upstream Process Sciences, Braine l’Alleud, Belgium

###### **Correspondence:** Jonathan Stern

**Background**

High performing cell culture processes enable to reach high cell culture densities and also high final product titer. The increased process performance also leads to increased levels of impurities during bioreactor production. These impurities can then impact primary recovery, downstream process and also the performance of in-process analytical methods.

For the successful development of cell culture harvest process, it is essential to monitor the concentration of the product of interest to calculate process yield. It is typically measured by HPLC using affinity resin coupled to an absorbance detector. It is well known that process related impurities such as DNA, or HCP may interact with monoclonal antibody and therefore may interfere in the quantification assay.

Since the population of impurities is changing from one clarification step to another, their impact on the analytical quantification method needs to be assessed to calculate accurate clarification yields during primary recovery.

**Materials and methods**

To explore the impact of impurities on Protein A HPLC method accuracy, we spiked in-process samples with purified product at different stages of the clarification process and injected variable amount of sample in the analytical column. We also assessed the impact of a pre-treatment agent known to remove negatively charged impurities at neutral pH.

In a second stage of the study, we collected the material eluted from the analytical column and performed absorbance spectrum acquisition (Waters ACQUITY PDA) and SDS-PAGE analysis in reduced and non-reduced conditions.

**Results**

Reduction in the titer measured by 30% after the application of the pretreatment during the clarification process (Table 1). No effect of pre-treatment was seen on Drug Substance (DS) suggesting that the decrease in titer, measured after pre-treatment, would not be caused by a loss of the desired product as it is in the DS.

The analytical quantification method was demonstrated to be linear and accurate for in-process samples with and without pre-treatment.

No major differences in Protein A HPLC elution peak absorbance spectrum was found. Also, the ratio A260/A280 was consistent between DS, pre-harvest and post pre-treatment in-process samples, which is not in favor of DNA interference in the quantification of non-treated samples.

No visible difference in Protein A HPLC elution peak gel electrophoresis profiles between Pre-Harvest and Post-pre-treatment samples (Figure1). Although, a clear diminution of the number of bands following pre-treatment of the samples neat could be seen, no evidence of any of those bands (potential HCPs) contaminating the Protein A HPLC elution peak was observed.

**Conclusions**

In this study, we investigated the decrease of measured titer after pre-treatment. We showed that this observation was not directly caused by the precipitation of the product. Also, the robustness and accuracy of the analytical quantification method was found very similar for sample before and after pre-treatment. From the data collected, no clear evidence of DNA or HCPs role in the decrease of measured titer could be demonstrated. Nonetheless, it is possible that strong interactions between process related impurities and the product might have been undetected with the methodology applied in this study. To explore further this hypothesis, impact of DNAse treatment prior quantification will be assessed. Also, to investigate further the purity of the Protein A HPLC elution peak, 2D HPLC methods and LC-MS/MS peptide mapping analysis will be performed to highlight potential co-elution of product with HCPs.

**Table 1 (abstract P-423).** Evolution of Protein A HPLC titer during clarification process

Step	N=	Normalized average titer	%RSD	%Loss Titer^**a**^
Pre-Harvest	3	0.91	9.6	
Post-pre-treatment	3	0.64	12.8	30
Post-Centrifugation	3	0.61	7.3	33
Post-Depth Filtration	3	0.64	4.6	30
Post-Sterile Filtration	3	0.60	2.9	33

^a^Relative differences between product titer measured after each harvest step, compared to Pre-Harvest titer

**Fig. 1 (abstract P-423).**
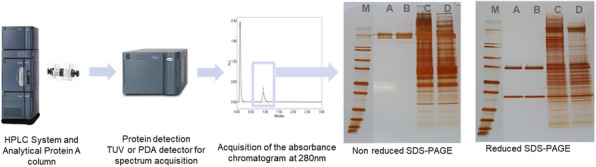
Non-reduced and reduced SDS-PAGE of neat. M, Molecular Weight Marker; A, Protein A HPLC Pre-Harvest elution peak; B, Protein A HPLC Post pre-treatment elution peak; C, Pre-Harvest sample neat; D, Post pre-treatment sample neat

### P-429 Automation of high-throughput titer assays for cell line development

#### Christian Meissner, Sebastian Giehring

##### PAIA Biotech GmbH, Gottfried-Hagen-Straße 60-62, 51105 Cologne/Germany

###### **Correspondence:** Christian Meissner (sebastian.giehring@paiabio.com)

**Abstract**

High-throughput methods for screening and automation in bioprocess development have been introduced into most industrial labs over the past years and paved the way for screening larger sample numbers, resulting in higher product yields as well as shorter development time. We present a comparison of automated PAIA titer assay with current technologies (biolayer interferometry) and additionally show that rather new and relatively inexpensive liquid handling systems are capable of handling the sample preparation of PAIA assays with good speed and accuracy.

**Background**

Automation in bioprocess development is usually associated with rather high investments into hardware like liquid handlers and software. Depending on the specific needs, automation may integrate several parts of the workflow, e.g. clone imaging, hit picking, titering and different liquid handling operations associated with these tasks. The combination of the Cellavista imager (SynenTec GmbH) the PAIA titer assay PA-104 and low-cost liquid handlers represent an affordable solution for single cell cloning, cell counting and titer measurements in high throughput.

**Materials and methods**

The PA-104 Fc low titer assay (PAIA Biotech, Cologne, Germany) was used for all experiments. It contains 384-well plates loaded with capture beads and an assay reagent containing fluorescence labelled Protein A. The dynamic range of the assay ranges from 5-200 μg/mL. The workflow of the assays is as follows:

1. Addition of 35μL PAIA mix reagent per well.

2. Addition of 5μL cell culture supernatant or standard.

3. Shaking on orbital shaker for 15 mins.

4. Bead settling for 5 mins or centrifugation at 500 g for 1 min.

5. Read out on Cellavista with red fluorescence bottom reading.

Fluorescence plate readers can also used to read the assay plates.

**Results**

Firstly, we conducted a benchmarking study and compared the PA-104 with an Octet Red 384 (ForteBio, Menlo Park, USA) equipped with ProteinA biosensors. Samples from 96-DWP (deep well plate) cultures were diluted in PBS by a factor of 50, split and subsequently analysed on both systems in parallel. The whole sample preparation was carried out on a Microlab Star (Hamilton,Bonaduz, Switzerland), and the read-out was performed with Cellavista imager (SynenTec GmbH, Elmshorn, Germany).

Secondly, we evaluated low-cost liquid handling solutions and examined whether they are sufficiently precise for the sample preparation of PA-104 assays (Table1).

**Conclusions**

The use of low-cost liquid handlers such as the OT-2 and the Viaflo Assist Plus provide cost efficient automation for the sample preparation for PAIA assays

The OT-2 offers sufficient deck space for a typical workflow where e.g. samples from 96 well formats have to be transferred into one 384 well PAIA plate.

The OT-2 is not much slower than the Hamilton Star, unless the Hamilton is equipped with a 96-channel head, which provides additional time savings.

**Fig. 1 (abstract P-429).**
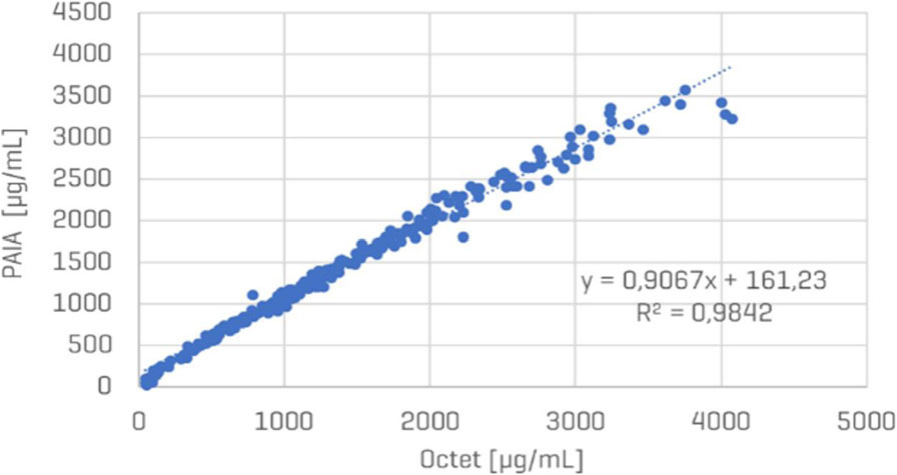
Clone Screening from 96-DWPs with the PA-104 and correlation with the Octet Red 384 system

**Table 1 (abstract P-429).** Evaluation of liquid handling solutions

Model	Viaflow Assist Plus	OT-2
Manufacturer	Integra Biosciences	Opentrons
Deck positions	4, plus tip rack and waste	11, plus waste
Pipet head options	Different pipets from the Viaflow series (flexible tip spacing available)	Different pipets (fixed tip spacing)
Programming	Separate software	Python
Pipetting accuracy 50μL PAIA reagent	CV = 2%	CV = 2%
Pipetting accuracy 5μL Sample	CV = 2%	CV = 2%
Workflow time for preparation of 4 96-well plates	45 minutes using one 8 and one 16 channel pipet	30 minutes using two 8 channel pipets
No. of manual interventions	4, for exchange of plates and pipets	none

### P-430 IgG Glycan Screening Assays In Crude Cell Culture Supernatants

#### Sebastian Giehring^1^, Christine Wosnitza^1^, Jürgen Fieder^2^, Martin Gamer^2^, Anna Johann^1^

##### ^1^PAIA Biotech GmbH, Gottfried-Hagen-Straße 60-62, 51105 Köln, Germany; ^2^Boehringer Ingelheim Pharma GmbH & Co.KG, Cell Line Development, Bioprocess Development Biologicals, Biberach, Germany

###### **Correspondence:** Sebastian Giehring (anna.johann@paiabio.com)

**Abstract**

Glycosylation of antibodies is a critical quality attribute (CQA) which affects stability, aggregation, serum half-life and immunogenicity of the drug substance. We present a novel technology that overcomes current analytical bottlenecks for antibody glycosylation and allows for fast and reliable screening of non-purified cell culture samples using PAIA assays.

**Background**

The control of glycosylation during cell line development receives increasing attention because different studies have shown that small scale fed-batch cultures (e.g. in deep well plates) are not only predictive for product titers but also for glycosylation at larger scales [1]. Since modifications of the cell culture process, e.g. by media supplements, can only change product glycosylation within the capacity of a given cell line [2], it is critical to include glycosylation screening when selecting cell lines, especially if a defined glycosylation profile needs to be achieved (e.g. in biosimilars).

**Materials and methods**

We used the PAIA glycan screening assay kit PA-201 (PAIA Biotech, Cologne, Germany) which contains capture beads for IgG and fluorescence labeled lectins that detect different types of glycosylation, e.g. fucosylation, galactosylation, mannosylation and sialylation. The assay can detect glycans on glycoproteins in a one-hour, high throughput and completely microplate-based assay. Here, we describe a novel protocol for the PA-201 which eliminates the need to purify the sample. Interfering components from the supernatant are removed with an integrated purification step carried out in the 384-well PAIAplates using an automated liquid handling system (Viaflo assist Plus, Integra Bioscience). At first, the IgG in the sample is bound to the capture beads. After the beads have settled, the supernatant is carefully replaced with buffer three times. Afterwards, the glycan markers are added and the plate is shaken on an orbital shaker for 45 min. After bead settling (15min) or centrifugation (1min), the plate can be measured on a plate reader. The whole process can be easily automated.

**Results**

24 samples from an ambr15 cell culture run were diluted to a concentration of 100 μg/mL and 100 μL of the dilution was mixed with PAIA sample treatment solution (1:1). The resulting mixture was heated (80° C, 5 minutes) on an Eppendorf Thermomixer. After cooling down, 10 μL of the treated sample were combined with 50 μl of lectin reagent per well; different lectins were probed in separate wells. Thereby, glyco-profiles for fucosylation, galactosylation and mannosylation were generated for all samples. Figure 1 shows the results for the high Mannose binding lectin in correlation with the MS analysis obtained by Boehringer.

**Conclusions**

The PAIA glycosylation assay PA-201 provides a fast method to screen cell culture supernatants and determine relevant differences in product quality as early as in 96 deep well plates. The new protocol presented here can identify cell lines with a tendency to produce highly mannosylated antibodies so that these can be discarded at an early stage.

**References**

1. Rouiller et al. (2016) Screening and Assessment of Performance and Molecule Quality Attributes of Industrial Cell Lines across Different Fed-Batch Systems. Biotechnol Prog 32(1):160-170.

2. Loebrich et al. (2019) Comprehensive manipulation of glycosylation profiles across development scales. MAbs 11(2):335-349.

**Fig. 1 (abstract P-430).**
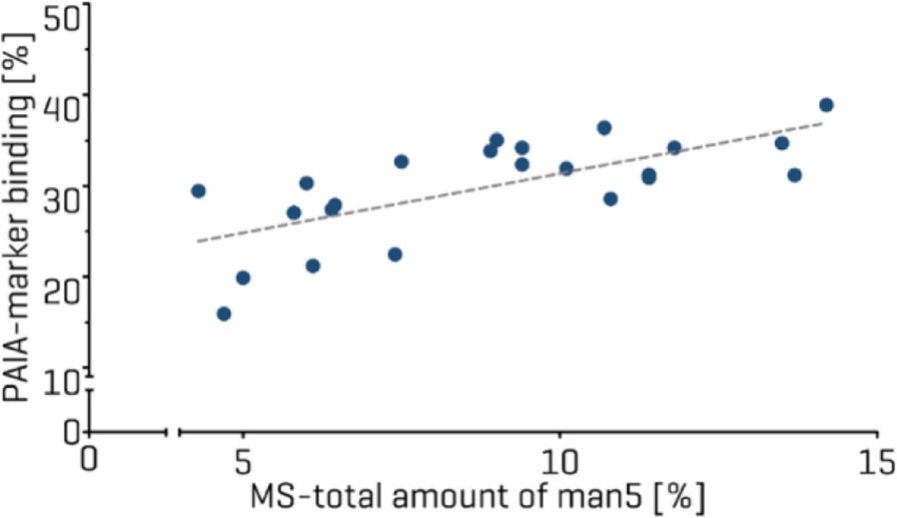
Clone screening for High Mannose with the PA-201 glycan assay and correlation of lectin binding rate of the Man-5 binding lectin with the results from MS peptide mapping results from Boehringer

### P-432 Define the undefined - Towards real chemically defined media in cell culture

#### Anja Wüst, Anica Schmidt, Sandra Klausing, Christoph Heinrich, Stefan Northoff

##### Xell AG, Bielefeld, Germany

###### **Correspondence:** Anja Wüst (anica.schmidt@xell.ag)

**Background**

Nowadays stable and reproducible bioprocesses are needed to guarantee biopharma productions with low lot-to-lot variations. The medium formulation as an important influence factor has to be controlled to achieve high medium consistencies. The impact of impurities in raw materials is foremost process and cell line dependent, therefore the specific response has to be checked individually. The usage of controlled high-quality products in medium production can exclude cost intensive screenings for the impact of single components on product quality and process stability. Trace elements are the most popular impurities of components used in cell cultivation media with a known impact on process performance [1].

**Materials and methods**

Amino acids are major components in media formulations used in cell cultivation, therefore an analytical screening of different proteinogenic amino acids was performed to evaluate contaminations for 12 different suppliers (amino acid-dependent) and quality grades. The quality grades varied between pur. (purum; 97%) up to p.a (pro analysis) and amino acids signed as cell culture reagent. The chemicals were solubilized at different concentrations (solubility-dependent) and analysed via ICP/MS.

**Results**

The results illustrate amino acid-dependent impurity profiles. The impact of impure raw materials on process performance is cell line- as well as product- dependent and has to be evaluated separately. Known contaminations that can have an impact on cell cultivation processes at low concentration levels are trace elements like iron, zinc, copper and manganese. Less investigated are nickel, chrome and molybdenum, which are also detectable as impurities. Most of the tested amino acids in this study were without traceable impurities for tested elements. For glutamic acid, weak concentrations of chrome (0.47 μg/g for 99% quality grade) and copper (11 μg/g for pure quality grade) were measured (data not shown). In addition, a high iron concentration in one asparagine batch up to 26.5 μg/g (98% quality grade) could be detected (data not shown). The trace element impurity amount was highest for the proteinogenic amino acids tyrosine and cystine (Figure 1). Detailed analytics showed highest concentration values for iron (up to 1070 μg/g) and molybdenum (up to 85 μg/g) impurities in cystine of different suppliers. High contamination values of tyrosine and tyrosine sodium salt batches were detected for iron (309 μg/g), molybdenum (74.7 μg/g) and chrome (19 μg/g).

**Conclusion**

We observed concentration variations of trace elements in proteinogenic amino acids from different suppliers and quality grades. Substantial impurity profiles were measured for cystine and tyrosine, whereas all other components show significantly lower values of trace elements, independent of the tested quality grade. The data indicated no concrete trend in terms of higher trace element contaminations for lower quality grades, therefore the degree of impurity has to be checked individually. As the effect of trace elements is highly component-, cell line- and product-specific, lower concentration ranges (data not shown) have to be taken into account as well for a precise chemical definition of a medium. In addition, all other components of cell culture medium and feed formulations are under investigation, concerning the impact of trace elements and other possible impurities.

**References**

1. Mohammad A., Agarabi C., Rogstad A., DiCioccio E., Brorson M.A., Faustino P.J., Madhavarao C.N. An ICP-MS platform for metal content assessment of cell culture media and evaluation of spikes in metal concentration on the quality of an IgG3:κ monoclonal antibody during production. J Pharm Biomed Anal. 2019; 162:91-100.

**Fig. 1 (abstract P-432).**
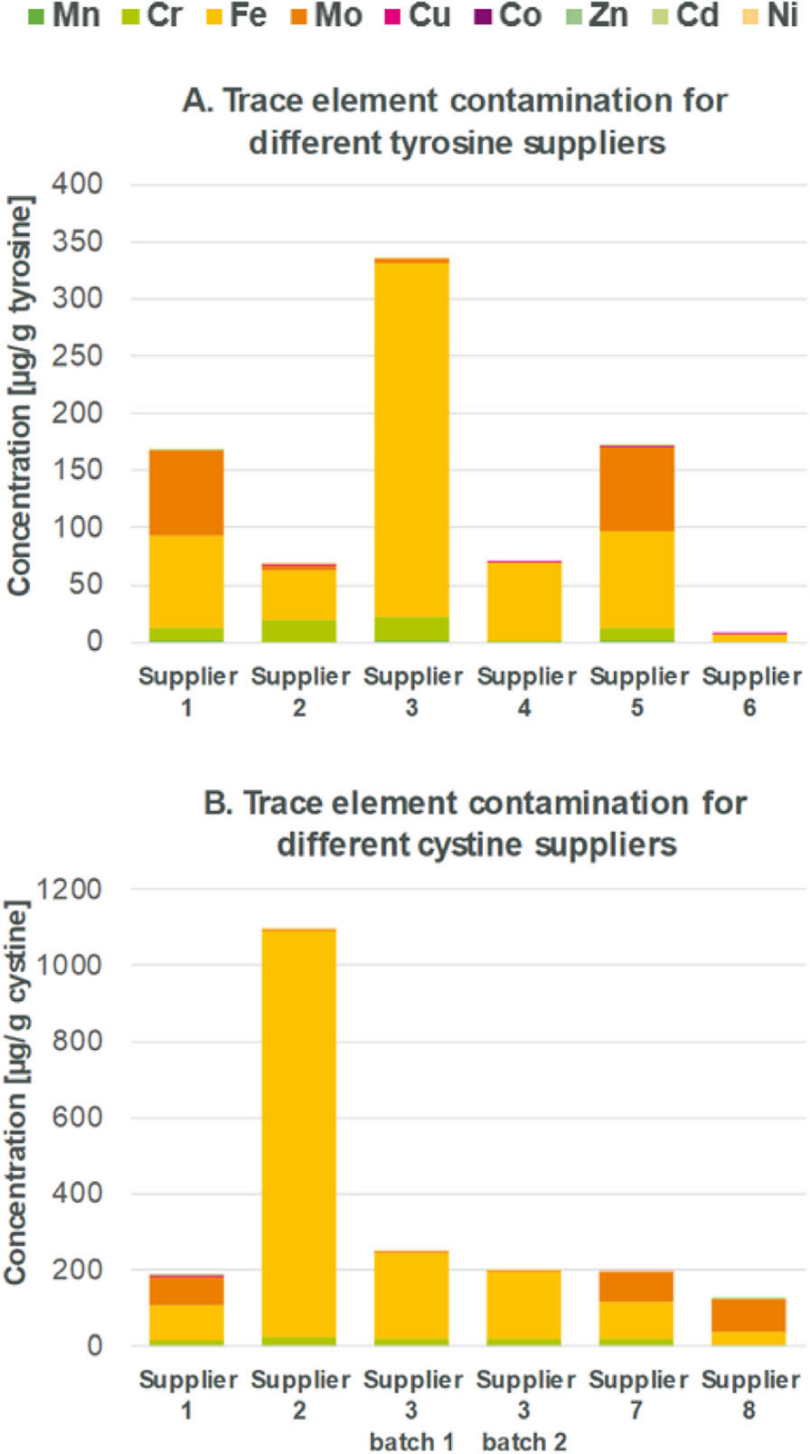
The impurity profiles of trace elements for different suppliers of tyrosine (Fig. A) and cystine, whereas two different purity grades where measured for supplier 3 (Fig. B)

### P-440 Enhanced method to monitor cell cultures by dielectric spectroscopy

#### Daniel Arturo Zavala-Ortiz^1,2^, Bruno Ebel^1^, Meng-Yao Li^1^, Maria Guadalupe Aguilar-Uscanga^2^, Javier Gomez-Rodriguez^2^, Dulce Maria Barradas-Dermitz^2^, Patricia Margaret Hayward-Jones^2^, Annie Marc^1^, Emmanuel Guedon^1^

##### ^1^Laboratoire Réactions et Génie des Procédés, CNRS-Université de Lorraine, Vandœuvre-lès-Nancy, France; ^2^Laboratorio de Bioingeniería, Instituto Tecnológico de Veracruz, México

###### **Correspondence:** Daniel Arturo Zavala-Ortiz (bruno.ebel@univ-lorraine.fr)

**Background**

Physiological state of cells has a strong impact on post-translational modifications of biopharmaceuticals. Therefore its accurate monitoring and control is mandatory to guarantee medicines properties and safety of patients. The specific cell growth rate (μ) may globally be used to depict the cells state during cultures. Using *in-situ* dielectric spectroscopy, μ can be used to control the feeding strategy of cell cultures so that glycosylation quality of monoclonal antibody (mAb) remained under proper levels [1]. However, the widely used simple linear regression (SLR) of measured permittivity to real-time estimate the viable cell density (VCD) and then calculate μ, can led to a lack of accuracy and precision. To avoid limitations, this study aimed to evaluate the novel implementation of Supported Vector Regression (SVR) on dielectrics.

**Materials and methods**

Capacitance spectra were collected every 12 min by using Biomass Evo 200 during several mAb producing CHO cells cultures in bioreactors (2L). Off-line measurements of VCD were performed using the Trypan Blue exclusion method (ViCell, Beckman Coulter). Then VCD values were related to measured permittivities by both regression methods (SLR and SVR) to build prediction models. The SLR model was generated using the linear equation:

$$ \mathrm{VCD}=\kern0.5em \mathrm{a}\ \mathrm{x}\ \left({\mathrm{permittivity}}_{1000\ \mathrm{kHz}}\right)+\mathrm{b} $$

*where a and b are fitting coefficients*

The SVR model was generated using an ε-support vector regression with a Gaussian radial basis function kernel (PLS_Toolbox® 8.2.1, Eigenvector Research). Then, the *in-line* estimated VCD was used to calculate μ values in real-time, and compared them to off-line values for evaluation:

$$ \upmu =\frac{\Delta \ Ln\ (VCD)}{\Delta  t} $$

**Results**

The SLR uses a variable (permittivity) space to perform regression. A consideration for such model is that cell properties, as well as relation of VCD to permittivity spectra, remain constant. This is not the case during the various phases of culture process. Consequently, the SLR can not well relate cell subpopulations displaying different dielectric properties, mainly during late-stationary and dead phases. On the contrary, the SVR method, based on a sample space, demonstrated a remarkable robustness for tracking cells having different dielectric properties (Figure 1-a). SVR creates a sample distribution based on dielectric properties before generating the regression equation, allowing considering variability of cell dielectric properties within samples.

Then, both SLR and SVR models were used to in-line calculate μ. The SVR method leads to more accurate and stable calculations of μ during cultures (Figure 1-b).

**Conclusions**

On the basis of results, the SVR method must be favoured in order to improve the monitoring of animal cell culture processes by dielectric spectroscopy, especially when the composition of the culture medium changes significantly or when cells are subject to strong physiological changes. SVR also appears promising to develop new approaches taking into account the different dielectric subpopulations of cells rather than assuming a homogeneous dielectric population within cell cultures in bioreactor [2].

**Acknowledgements**

The authors acknowledge CONACyT and Campus France which granted Daniel. A. Zavala-Ortiz, and the French National Agency for Research (ANR) for supporting the ProCell-In-Line project.

**References**

1. Li M-Y, Ebel B, Blanchard F, Paris C, Guedon E, Marc A. Control of IgG glycosylation by *in situ* and real-time estimation of specific growth rate of CHO cells cultured in bioreactor. Biotechnol Bioeng. 2019; 116(5):945-1252.

2. Braasch K, Nikolic-Jaric M, Cabel T, Salimi E, Bridges GE, Thomson DJ, Butler M. The changing dielectric properties of CHO cells can be used to determine early apoptotic events in a bioprocess. Biotechnol Bioeng. 2013; 110(11): 2902–2914.

**Fig. 1 (abstract P-440).**
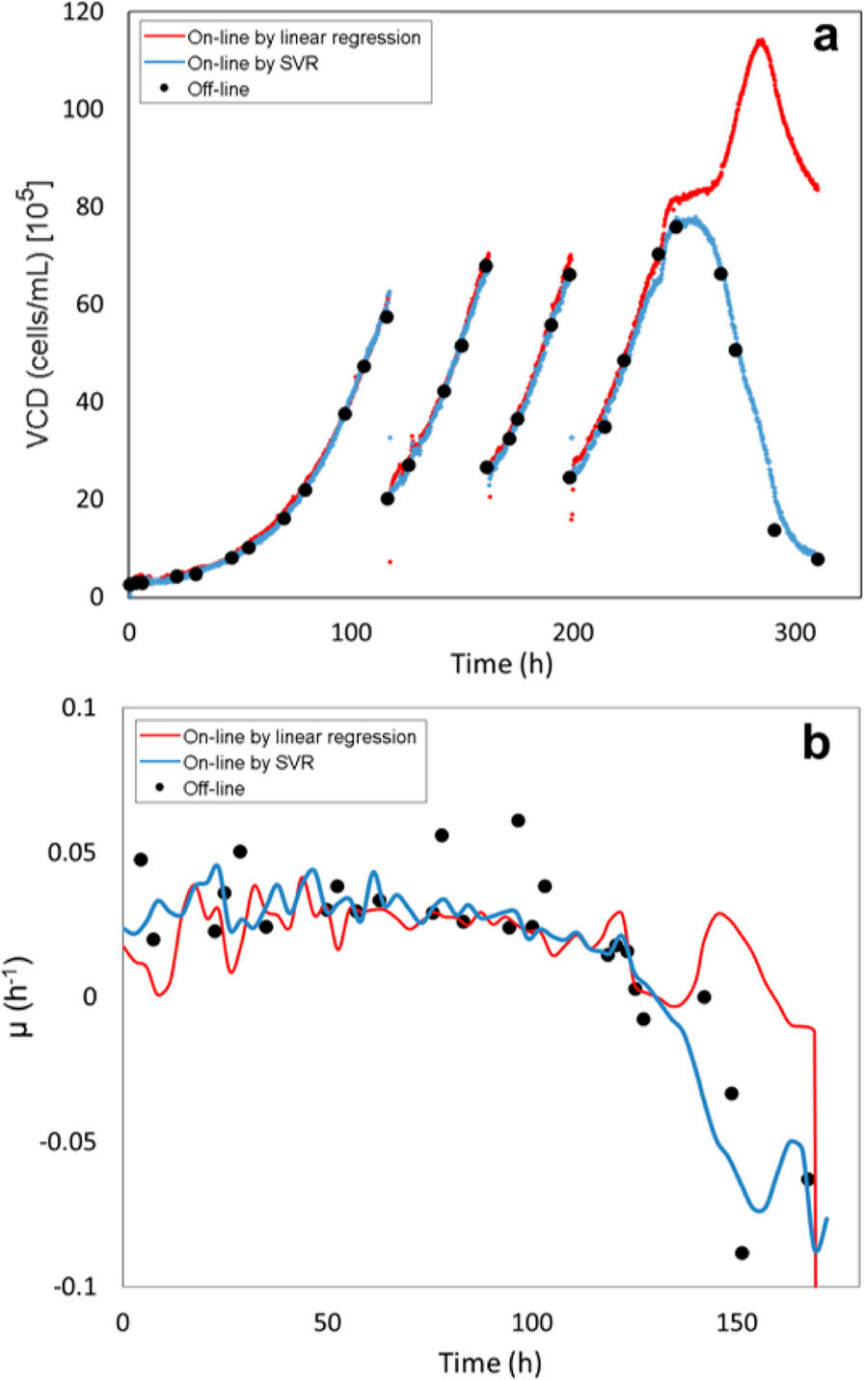
Use of SVR and SLR models to monitor (a) VCD in fed-harvest culture and (b) μ in batch culture

### P-441 Suffer your media from burn-out? – Analyzing stressed media

#### Tim Steffens, Anica Schmidt, Carolin Fritzenkötter, Christoph Heinrich, Stefan Northoff

##### Xell AG, Bielefeld, Germany

###### **Correspondence:** Tim Steffens (tim.steffens@xell.ag)

**Background**

Did you leave your medium on the bench or have you incubated it for sterility testing before use? Process preparation and surrounding conditions might play a crucial role related to the performance of a medium. Here we present data generated after exposure of media to different conditions other than the cellular influence. The aim was to determine how the induced conditions applied to cell culture media result in molecular changes which in turn might affect the media performance. We incubated medium at 33°C and 37°C in a bioreactor system without cells and exposed it to different light conditions. We observed that the tested conditions can alter culture performances significantly.

**Materials and methods**

**Vitamins** were measured with Xell’s proprietary method via LC-MS/MS on a Varian 2000 QQQ coupled to a ProStar HPLC or an Agilent 6470 QQQ coupled to an Agilent 1290 UPLC.

**Amino acid** measurements were carried out with Xell’s proprietary method on an Agilent 1290 UPLC. Derivatized amino acids were detected with a DAD.

Duplicate **batch cultivations** of a CHO GS cell line were carried out in stressed vs. reference media in 125 ml shake-flasks. 37°C, 5% CO_2_, 80% humidity and 185 rpm were applied. Cell density and viability were measured on a Cedex cell counter.

**Molecule identification** was done by LC-MS full scans on an Agilent 1290 UPLC coupled to an Agilent 6470 QQQ. Accumulating molecules were identified via product scans at different CEs and database comparison of fragments.

**Results**

Cell culture media were exposed to sun and artificial light for 14 days. During that time several samples were taken. 27 amino acids plus 12 vitamins were profiled and compared to media that was stored in the dark. Next to slight alterations to e.g. folic acid, riboflavin and cyanocobalamin show severe decays after light treatment. Thiamine and ascorbic acid were unstable at all conditions. Cysteine is rarely detected in cell culture media, due to the conditions in the media matrix. Even in water cysteine shows a fast conversion towards cystine, when no other binding partners are present (Table 1). It can be expected to react even faster in the presence of different molecules in cell culture media.

Treated media were used for cultivations. Storage at 21°C and sunlight induced vast performance limitations starting at day 3. Artificial light at 4°C induced notable performance limitations after 7 days. Storage in the dark did not result in growth performance deficiencies. (Figure 1).

Bioreactor runs at 33°C and 37°C without cells were carried out to test the influence of cultivation conditions on media. No vitamin or amino acid decomposed completely. However, MS full scans revealed the generation of molecules over time. The approach to identify unknown molecules is shown with one example. Detected masses of unknown molecules were used as targets for product ion scans. The fragments could then be used for a database search [1]. In this case the fragments and their distribution could be mapped to oxoproline, which originates from glutamine after deamination. To further validate the result, we measured the glutamine decay and increase of ammonium (Table 2).

**Conclusion**

Exposure to light alters cell culture media significantly, cultivations resulted in reduced growth, aggravating with exposure time. Further, notable differences between sun- and artificial light could be detected.

Cysteine is rarely detected in cell culture media and even displays its highly reactive nature in water.

Accumulation of molecules, such as oxoproline, was observed during cell-free bioreactor runs, which might reduce the growth performance.

**Reference**

1. Domingo-Almenara X, Montenegro-Burke JR, Ivanisevic J, Thomas A, Sidibé J, Teav T, Guijas C, Aisporna AE, Rinehart D, Hoang L, Nordström A, Gómez-Romero M, Whiley L, Lewis MR, Nicholson JK, Benton HP, Siuzdak G. XCMS-MRM and METLIN-MRM: a cloud library and public resource for targeted analysis of small molecules. Nature Methods. 2018; 15:681-684.

**Table 1 (abstract P-441).** Time-dependent distribution of cysteine and cystine

	Duration until sampling	Cysteine (rel. Amt.)	Cystine (rel. Amt.)
**1 mM Cysteine**	0 h	0.89	0.11
	2 h	0.91	0.09
	5.5 h	0.90	0.10
	24 h	0.65	0.35
	26 h	0.52	0.48
	6 days	0.02	0.98

**Fig. 1 (abstract P-441).**
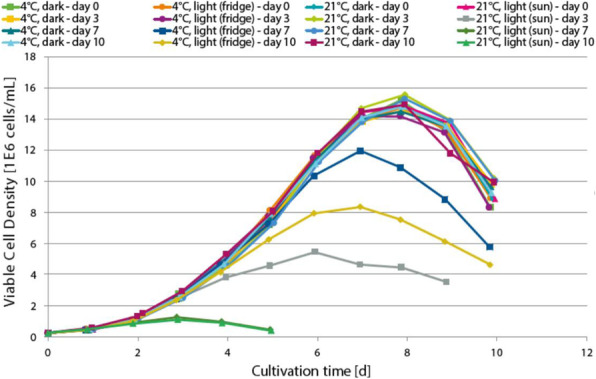
Growth performance of a CHO cell line cultivated in media stored at different light conditions

**Table 2 (abstract P-441).** A. Mass analyses that led to the identification of oxoproline. B. Glutamine and ammonium measurements of medium from bioreactors (10 days 33°C and 37°C) against a control medium (10 days 4°C)

**A.**	**Mass (m/z)**		
Parent Ion	130.3		
Fragments	41.3	56.2	84.1
**B.**		**Difference Glutamine**	**Difference Ammonium**
10 days incubation, 4°C vs 33°C		-2.57 mM	+2.54 mM
10 days incubation, 4°C vs 37°C		-3.46 mM	+3.48 mM

### P-447 Case study: Unexpected impact of shear stress on intensified fed-batch process

#### Nandita Vishwanathan^1^, Carole Chantelauze^1^, Sandrine Richard^1^, Damien Voisard1, Vincent Monchois^1^, Matthieu Stettler^1^, Miroslav Soos^2^, Massimo Morbidelli^3^, Hervé Broly^1^

##### ^1^Bioprocess Sciences, Merck, Corsier-sur-Vevey, Switzerland; ^2^VŠCHT Praha - UCT Prague, Prague, Czech Republic; ^3^SciMod s.r.l, Milano, Italy

###### **Correspondence:** Nandita Vishwanathan (nandita.vishwanathan@merckgroup.com)

**Background**

Process intensification by high cell density perfusion seed culture has been rapidly embraced in recent years by biopharma to boost volumetric productivity [1]. At Merck Serono, a Chinese hamster ovary (CHO) cell line-based platform process was intensified using an (N-1) perfusion seed culture. The tangential flow filtration (TFF)-based system enabled seeding the production bioreactor at high cell density, and thereafter achieved high yields with comparable product quality. This case study presents a challenge faced during the scale-up of this process to the manufacturing scale.

During scale-up, in the (N-1) seed bioreactor, a lower growth rate and loss of viability (Figure 1B&C) were observed along with a reduction in cell diameter linked to the start of recirculation (Figure 1C). These effects were not observed before in small-scale. A potential impact of a high shear stress environment in the (N-1) seed bioreactor’s external perfusion loop (TFF unit) at manufacturing scale represented the main cause to investigate.

**Materials and methods**

A computational fluid dynamics (CFD) simulation of the (N-1) skid elements was performed individually at the process conditions. The results from all these individual elements were combined to estimate the equivalent shear stress in the recirculation loop (Figure 1A). A small-scale model (similar to published work [3]) using a nozzle constriction in the recirculation loop was developed to test higher shear stress levels at small scale. A series of CFD simulations of the small-scale model was performed with different nozzle diameters to determine the appropriate range of diameters (Figure 1B) to mimic the maximum shear stress levels observed at manufacturing scale. Several nozzles in this range were fabricated in stainless steel and placed at the outlet of pump in the recirculation loop of the small-scale model. The (N-1) bioreactors were seeded and the cells’ physiological character-istics was compared between the two scales.

**Results**

The small-scale shear stress model was able to mimic the behavior of the manufacturing run. With decreasing nozzle diameters from 2.5 mm to 1.5 mm (increasing shear stress) and increasing pressure drop across the nozzle from 0.2 bar to 0.4 bar (increasing shear stress), effects seen at manufacturing scale such as reduced cell growth (Figure 1G), reduced viability (Figure 1H), and reduced cell diameter (Figure 1I) could be reproduced. For example, the cell diameter at the end of the run decreased to 13 μm from 14 μm at the two extremes (Figure 1I). In comparison, the cell diameter at manufacturing scale was 13.5 μm (Figure 1D) on the same day of the (N-1) indicating that level of shear stress at manufacturing scale was lower than the maximum level of shear stress experimented at small scale. Similar effects of high shear on mammalian cells have been reported in literature [2].

Thus, using the small-scale shear stress model, it was confirmed that the behavior observed at manufacturing scale was indeed due to shear stress. Thereafter, the CFD model of the manufacturing elements was able to identify high shear elements, which were then modified to remediate the shear stress impact.

Further adjustments of the process parameters (specifically, recirculation flowrate) were made to fine-tune the shear stress levels at manufacturing scale to bring it down to acceptable levels for the process and helped achieved desired cellular performance.

Surprisingly, for the cells impacted by shear stress in the (N-1) stage, when they were passaged into the production bioreactor, the cell growth was not negatively impacted in the subsequent step. The cell viability recovered within 3 days (Figure 1E&J) in both manufacturing scale and at small-scale. Even at the highest level shear stress application, the impact on the cells appears to be reversible.

**Conclusions**

A well-designed small-scale shear stress model is informative to mimic and predict behaviors in large scale bioreactors. CHO cells are rather robust and recover quickly (within three days) from exposure to high shear stress for up to five days.

**References**

1. Jordan M, Mac Kinnon N, Monchois V, Sttettler M, Broly H. Intensification of large-scale cell culture processes. Curr Opin Chem Eng. 2018; 22:253-257.

2. Godoy‐Silva R, Chalmers JJ, Casnocha SA, Bass LA, Ma N. Physiological responses of CHO cells to repetitive hydrodynamic stress. Biotechnol. Bioeng. 2009;103: 1103-1117.

3. Neunstoecklin B, Stettler M, Solacroup T, Broly H, Morbidelli M, Soos M. Determination of the maximum operating range of hydrodynamic stress in mammalian cell culture. J Biotechnol. 2015;194:100-109.

**Fig. 1 (abstract P-447).**
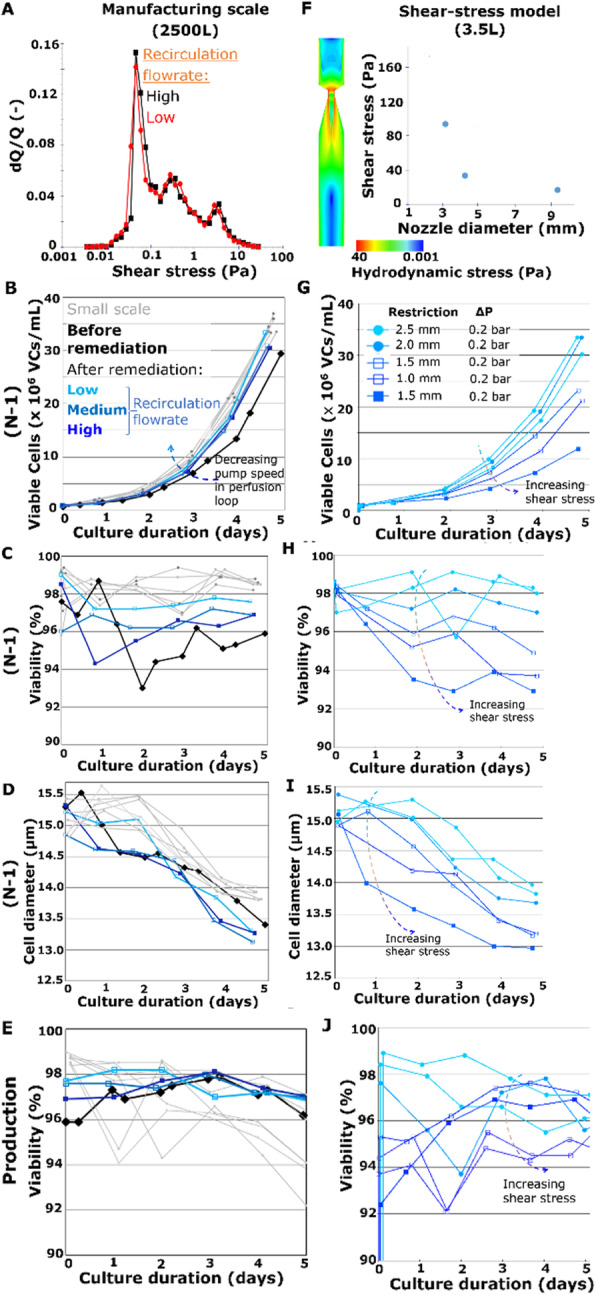
Simulation of shear distribution at manufacturing scale (A), shear level at small-scale model for different nozzle diameters (F). Impact of high shear simulation at manufacturing scale (B to D) small-scale (G to I) and on cell density, viability and cell diameter. Recovery of cell viability in production bioreactor at small scale (E) and manufacturing scale (J). In all the graphs, darker shades indicate highest shear stress, and among manufacturing runs, black run indicates the run before remediation of the manufacturing skid, colored runs are post-remediation, grey are small-scale reference for comparison

### P-448 High Throughput Glycosylation Assays for Glycoproteins Using Lectins

#### Christine Wosnitza, Anna Johann, Aris Perrou, Laura Limbach, Christian Meissner, Sebastian Giehring

##### PAIA Biotech GmbH, Gottfried-Hagen-Straße 60-62, 51105 Cologne/Germany

###### **Correspondence:** Christine Wosnitza (christine.wosnitza@paiabio.com)

**Abstract**

Glycosylation of therapeutic glycoproteins is a critical quality attribute (CQA) which affects different properties of the drugs such as stability, aggregation and serum half-life. It is critical to control glycosylation during cell line and bioprocess development because culture conditions have a great impact on product glycosylation and allow optimization of glycosylation properties, e.g. sialylation. We present data from a novel assay (PA-202) for different glycoproteins, discuss the influence of cell culture media and supernatants on the assay, and the applicability of the assays in supernatant screenings.

**Background**

Non-Mab glycoproteins can have very complex glycosylation patterns, making the analysis of many samples in parallel a difficult and time-consuming task. Moreover, purification of glycoproteins is necessary for analytical methods using released glycans. We present a bead-based assay that does not require the release of glycans and uses lectins to detect differences in glycosylation.

**Materials and methods**

The PA-202 assay (PAIA Biotech GmbH, Cologne, Germany) contains capture beads carrying a lectin- that specifically CHO-type 2,3-linked sialic acid which is present on a fluorescent labeled marker. The addition of glycoproteins in the sample releases the marker from the beads depending on its degree of glycosylation and its concentration. The release of fluorescent marker is measured directly in a no-wash assay in 384-well plates.

The workflow of the PAIA sialylation assay is as follows: marker and analyte are added to the wells of the PAIA plate. The plate is shaken for 45min on an orbital shaker. After settling of the capture beads, the released fluorescent marker is measured directly in the special 384-well PAIA plates.

**Results**

Different glycoproteins were probed with the PA-202 assay: approved drugs such as Epoetin alpha, Aflibercept, Etanercept and Prolastin, as well as experimental anti-alpha-trypsin (AAT) which was a kind gift of CEVEC Pharmaceuticals.

We produced calibration curves for these molecules in PBS and afterwards spiked the proteins into different cell culture media and supernatants to assess whether and to which extent they impact the assay. All cell culture supernatants show an increased baseline, whereas fresh cell culture media seem to have little impact on the assay, hinting at host cell proteins that may have a similar effect as the target proteins. It is therefore hypothesized that the assay enables the identification of highly sialylated products as long as the concentration of the product largely exceeds the HCP concentration.

The signal of each protein depends on the amount and availability of the terminal sialic acid (A,B). CAP-Go cell lines are glyco-engineered cell lines displaying different types of sialylation. As expected, AAT derived from CAP-Go.3 and human plasma show lower levels of 2,3-SA (B) compared to AAT from CAP-Go.1, but more 2,6 linked sialic acid (data not shown).

The impact of cell culture media and supernatants on the assay using AAT are in shown in C and D. All presented data were obtained in two independent experiments with technical triplicates.

**Conclusions**

The PA-202 sialic acid assay allows for the rapid analysis of glycoproteins. The assay format only needs little amount of sample, typically 5 or 10 μl per well at a concentration of approximately 200μg/mL. The results suggest that, despite some influence from host cell proteins, the assay can be performed with non-purified samples to identify differently sialylated proteins.

**Acknowledgements**

AAT CAP-Go.1, AAT CAP-Go.3, Prolastin®, cell culture media and supernatants were kindly provided by CEVEC Pharmaceuticals, Cologne, Germany.

**Fig. 1 (abstract P-448).**
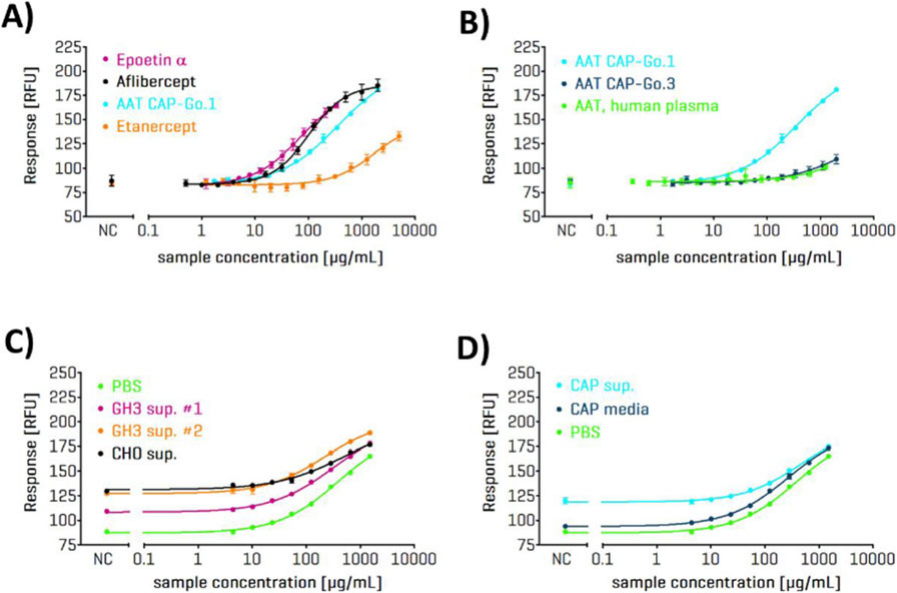
Quantification of 2,3-linked sialic acid on glycoproteins and Fc-fusion proteins

### P-453 Off-gas metabolite analysis for quality control and process control of mammalian cell culture

#### Daniel Becker^1^, Lena Schober^1^, Philipp Lebhardt^2^, Jessica Horbelt^1^, Andreas Traube^1^, Jens Langejürgen^2^

##### ^1^Fraunhofer Institute for Manufacturing Engineering and Automation (IPA), Department Laboratory Automation and Biomanufacturing Engineering, Nobelstraße 12, 70569 Stuttgart, Germany; ^2^Fraunhofer Project Group for Automation in Medicine and Biotechnology PAMB, Theodor-Kutzer-Ufer 1-3, 68167 Mannheim, Germany

###### **Correspondence:** Daniel Becker (daniel.becker@ipa.fraunhofer.de)

**Background**

The use of sophisticated production methods, such as cell-based bio production, is a growing trend that leads to high demand for novel quality control methods. To improve quality and productivity, these control methods should allow appropriate online monitoring and instant process control. To date, there is a lack of well-established process control strategies, especially for personalized products. Innovative analysis methods aim at non-invasive procedures that are able to rapidly extract large data volumes out of one single measuring point.

The goal of this study was to investigate the feasibility of a non-invasive and potential online measurement for monitoring cell production procedures based on a comprehensive off-gas analysis.

**Materials and methods**

Pilot experiments were performed using suspension-adapted CHO-S (Thermo Fisher Scientific) cells grown at 37 °C in 30 mL FreeStyle™ CHO Expression Medium (+ 8 mM L-glutamine) in 125 mL PC-Erlenmeyer flasks (Corning), applying commonly used cell expansion protocols with cell densities up to 7.0 × 10^6^ cells per mL. In addition, samples were taken every 24 h and centrifuged (15000 x g, 5min, 22 °C) to remove cells and cell debris. In the supernatant, glucose, L-glutamine, lactate and ammonium were analyzed, using a Cedex Bio Analyzer (Roche Diagnostics).

The off-gas phase of the culture was sampled (1 mL) through a sterile filter and analyzed using a gas chromatograph - ion mobility spectrometer (GC-IMS) two times per day over the whole cultivation process. Ion mobility spectrometry forms positively charged ions, of volatile organic compounds (VOCs). The gas sample becomes separated in two dimensions: first, the different VOCs are separated according to their polarity and boiling point in the GC column. Subsequently, these compounds are separated in a second dimension according their ion mobility. GC-IMS datasets were automatically processed and relevant features were extracted using an in-house developed algorithm (Python). This means, significant increases in detected ion currents at specific GC-retention and IMS-drift times are automatically identified as features. The prominence of each feature is then compared between each GC-IMS measurement [1,2].

**Results**

The analytics of the metabolites during the cultivation process of 48 h showed a decrease of glucose and L-glutamine (Figure 1 A). In contrast, the metabolites lactate and ammonium increased over time in accordance to the increasing viable cell density.

Cell metabolite analysis was subsequently compared to culture off gas analysis (Figure 1 B). Over 50 features that were not present in the background air or in the blank measurements could be identified in a single gas sample. The measurement points over time disclose variations in peak intensities and in the total amount of detected compounds for CHO cells. A comparison of feature prominence during a cultivation process shows that some features follow a continuous trend that can be either increasing or decreasing while others have a pronounced minimum or maximum.

**Conclusion**

Metabolic analysis using enzymatic kits and cell density control confirm the expected growth characteristics of the investigated cell culture process. Corresponding to this observation, the off-gas sampling showed no influence on cell growth.

The presented new sampling and measurement set-up illustrates a possible strategy to determine specific peaks and characteristics in the off-gas phase of a cell culture system. Furthermore, the determined signals correlate with the cell growth behavior and do not occur in the blank gas. Therefore, the experimental setup gives outlook to a new type of non-invasive soft sensor for cell culture applications.

**Acknowledgements**

This work has been supported in parts by the Ministerium für Wirtschaft, Arbeit und Wohnungsbau Baden-Württemberg.

**References**

1. Langejuergen J et al. Non-invasive monitoring of bacterial growth and auto-induced protein production in a bioreactor with a closed-loop GC-IMS. Int J for Ion Mobility Spectrometry. 2015; 18: 9-15.

2. Langejuergen J. Entwicklung und Realisierung kompakter Messsysteme zur quantitativen Detektion von organischen Spurengasen in Luft. In: Zimmermann S, editor. Berichte aus der Sensorik und Messtechnik Volume 1. Aachen: Shaker; 2015. p.1-173.

**Fig. 1 (abstract P-453).**
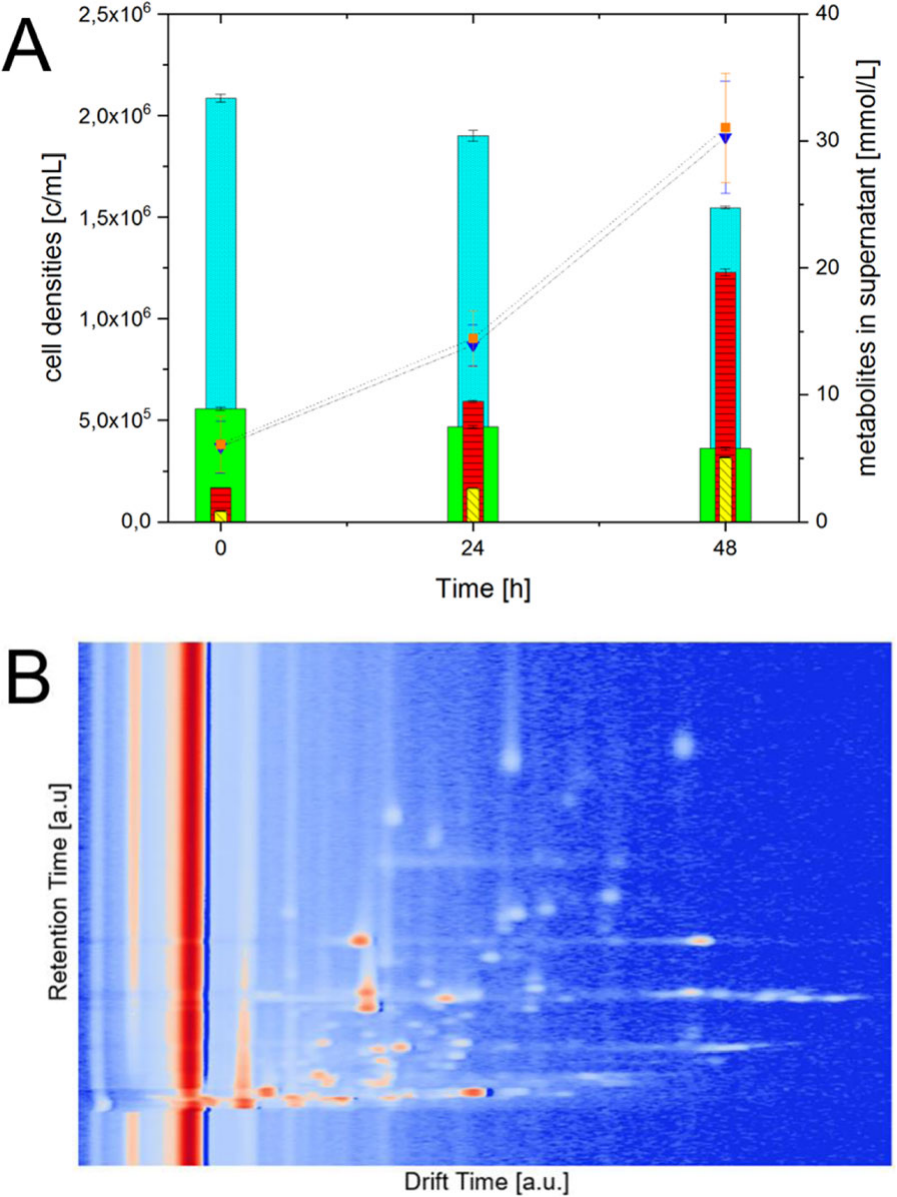
(A) CHO cell growth over a cultivation time of 48 h. Triangles = viable cell density; squares = cell density; substrate/metabolite concentrations [mmol/L]: lime-green bars = glucose; green bars = L-glutamine; red bars = lactate; yellow bars = ammonium. (B) Two-dimensional representation of the VOCs in the analyzed off-gas phase. The x-axis indicates the ion mobility of the compounds and the y-axis the retention time. Areas with high intensity (red), low intensity (blue). The sample was taken from a CHO cell culture after 43 h of cultivation

### P-508 High cell density clarification using single-use technologies

#### Martin Saballus, Lucy Nisser, Markus Kampmann, Gerhard Greller

##### Sartorius Stedim Biotech GmbH, Göttingen, Germany, 37079

###### **Correspondence:** Martin Saballus (martin.saballus@sartorius-stedim.com)

**Background**

Process intensification in biomanufacturing of therapeutic proteins enables increased productivities with simultaneous reduction of production volumes [1]. Promising upstream scenarios are high cell density (HCD) cultivations where mammalian cell concentrations of more than 100 million cells/mL (> 30 % wet cell weight, WCW) have already been reached [2, 3]. This development increases the pressure on downstream processing to develop scalable, robust and cost-effective single-use (SU) based clarification solutions [4]. In this study, different SU technologies, including depth filters (DF), dynamic body feed (DBF) filtration as well as a SU centrifuge system, were investigated regarding their ability to harvest such processes.

**Materials and methods**

HCD clarification experiments of CHO suspensions for the monoclonal antibody production were performed on a 1-10 liter scale. A depth filtration setup was tested comprising the double layer screening devices Sartoclear® Caps DL90 and DL20 with a filtration area ratio of 2:1. Furthermore, two different DBF filtration setups were tested using the Sartoclear Dynamics® Scale 20 including diatomaceous earth (DE) filter aid. The first DBF setup comprised a subsequent DL20 depth filter and a DE/WCW-ratio of 0.4. The second DBF setup included a pH lowering in the cell suspension to pH 5 by acetic acid and therefore a reduced DE/WCW-ratio of 0.25. In order to investigate the application of continuous SU centrifugation the kSep® 400 system was tested in a first feasibility study. This SU centrifuge is comprised of a disposable chamber and a tubing set, where cells and cell debris are retained in a fluidized bed by centrifugal forces of 1,000 x g.

The approaches were characterized regarding their process performance based on processability, biomass reduction, turbidity and filter capacity.

**Results**

In order to examine the applicability of filtration technologies various approaches were tested and compared to SU centrifugation (Figure 1). Due to its limited biomass loading capacity the tested DF approach was not suitable for HCD clarification. At DBF filtration high amounts of DE had to be added to achieve a sufficient permeability, even after a pH-shift. A way to handle the challenge of a poor processability at DBF filtration could be the dilution of cell broth.

As an alternative to overcome these limitations continuous SU centrifugation was tested (Figure 2). An almost cell free harvest pool with a significant reduced turbidity of 125 NTU was achieved. The separated cells in the waste pool showed a high viability what is considered as an indicator for a low release of host cell components. A product yield of 70 % was achieved. By an improved cyclic washing of cell pellets (performed in subsequent trials) yields were increased to more than 80 %. After post-centrifugal filtration a biomass free and sterile solution was obtained for further purification.

**Conclusions**

The tested filtration technologies showed limited applicability for HCD clarification due to their relatively low biomass loading capacities. However, SU centrifugation facilitates an almost complete removal of the biomass. An enhanced product yield was achieved by an integrated cyclic washing of the fluidized bed. This concept provides a promising approach for the development of a scalable and robust HCD clarification process solution. The next steps are focused on process parameter optimization due to its high potential to decrease buffer consumption and to increase yields.

**Acknowledgements**

The authors thank the complete BioProcessing team of Sartorius Stedim Biotech, Göttingen for all the efforts they put into generating these data.

**References**

1. Shukla AA, Thoemmes J. Recent advances in large‐scale production of monoclonal antibodies and related proteins. Trends in Biotechnology 2010, 28: 253-261.

2. Clincke MF, Mölleryd C, Zhang Y, Lindskog E. Very high density of CHO cells in perfusion by ATF or TFF in WAVE bioreactor™. Part I. Effect of the cell density on the process. Biotechnology progress 2013, 29: 754-767.

3. Matuszczyk JC, Schulze M, Janoschek S, Zijlstra G, Greller G. High cell density cell cultures (>100E6 c.mL^-1^) in 2D bags with integrated filter for seed train process intensification. Poster Bioprocessing Summit Europe 2018, Lisbon, Portugal.

4. Singh N, Arunkumar A, Chollangi S, Tan Z, Borys M, Li ZJ. Clarification technologies for monoclonal antibody manufacturing processes. Biotechnology and bioengineering 2016, 133: 698-716.

**Fig. 1 (abstract P-508).**
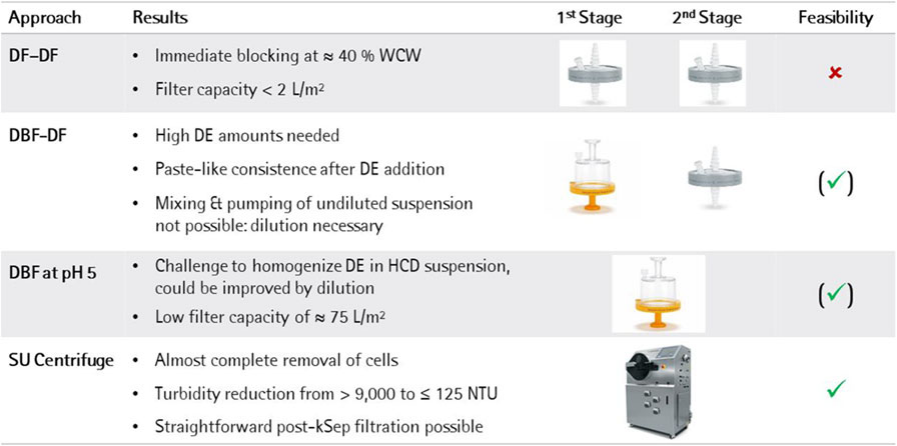
Overview of feasibility results of different HCD clarification approaches for the removal of 100 million cells/mL

**Fig. 2 (abstract P-508).**
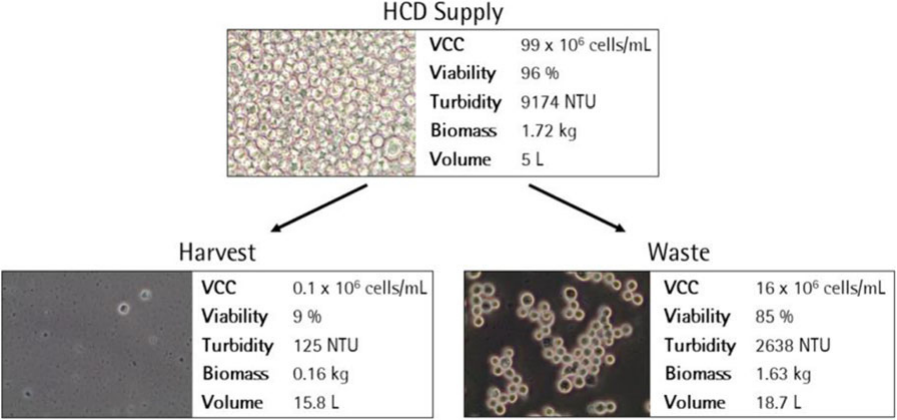
First results of a continuous clarification of 5 L HCD cultivation broth by SU centrifugation without subsequent filtration

### P-512 Media Additives Affect Antibody Quality Profiles In Perfusion Culture

#### Anelis Quintana^1^, Joaquin Antonio Solozabal^2^, Leina Moro^1^, Alexi Bueno^1^, Jose Arquimides Castro^1^, Tamy Boggiano^1^

##### ^1^BioProcess Development, Center of Molecular Immunology, Havana 11400, Cuba; ^2^I+D Quality Control, Center of Molecular Immunology, Havana 11400, Cuba

###### **Correspondence:** Anelis Quintana (anelis@cim.sld.cu)

**Background**

G1/G0-phase cell cycle arrest in recombinant cell cultures is often associated with increase in protein specific productivity, but the effects on product should be assessed because culture conditions could influence quality profile. This work evaluated not only cell growth and productivity, but also quality attributes during initial stages of process development, to map molecule quality as early as possible. This report also indicates that ligand–binding surrogate assays could serve as screening step to narrow media choices as critical variable from the very early preclinical development.

**Materials and methods**

A two factor complete factorial design in parallel cultures at 125 mL scale assessed the effect of G1/G0-phase cell cycle inhibitors (sodium acetate (NaAc) and a commercial feed (CF)) on productivity of an IgG4 recombinant CHO cell line. Protein A purified antibodies were characterized by gel filtration (GF) HPLC, SDS-PAGE and Western Blot. Antibody (mAb) functionality was evaluated by kinetic interaction of samples for PD1 antigen using a ligand capture technique on Biacore system (SPR).

**Results**

Results indicated that the increase of both substances caused a decrease on viable cells density, cell growth rate, cell cycle arrest with a diminishing in S phase, but production rate was not enhanced in any case. Media additives induced aggregation on the monoclonal antibody detected both in SDS-PAGE and gel filtration HPLC. Monomer content diminishing to values between 66 and 83,07% was deeply increased in connection with the presence of CF on culture media (Table 1), and this quality parameter had a significantly inverse correlation with antibody binding affinity to the ligand into a narrow nanomolar range (91,02 to 166,25 nM). Differences in similarity scores (SS) were mainly related with changes in association rate constant and hence in the equilibrium dissociation constant [1].

A higher molecular weight band was present in all lanes which intensity grew from control to those samples treated with sodium acetate and CF (Figure 1). These results indicated that the addition of those components provokes a decrease in the monoclonal antibody purity [2]. Samples also showed lower molecular weight bands that could be artifacts formed during sample preparation.

**Conclusions**

Cell arrest in G1/G0-phase of the cell cycle resulted in diminishing of S phase cell fraction and to a decrease in productivity. mAb aggregation level was increased in connection with the presence of cell booster in the media, and this quality parameter had a significantly inverse correlation with the antigen-antibody affinity.

**References**

1. Hayes JM, Frostell A, Karlsson R, Müller S, Millan-Martin S, Pauers M, ... & Rudd PM.. Identification of Fc gamma receptor glycoforms that produce differential binding kinetics for rituximab. Mol Cell Proteomics 2017: mcp-M117.

2. Handlogten MW, Lee‐O'Brien A, Roy G, Levitskaya SV, Venkat R, Singh S & Ahuja S. Intracellular response to process optimization and impact on productivity and product aggregates for a high‐titer CHO cell process. Biotechnol Bioeng 2018, 115(1): 126-138.

**Table 1 (abstract P-512).** Results of cell cycle arrest, purity by GF-HPLC, and similarity score by sensorgram comparison

Treatment	S Phase (%)	GF- HPLC (%)	SS in SPR (%)
Control	17,6±0,3	100,0±0,0	99,2±1,0
6 mM NaAc	13,6±1,8	99,2±1,0	99,9±0,0
12 mM NaAc	11,3±0,7	90,6±10,7	73,3±8,5
Control + CF	10,8±0,2	76,7±3,8	73,7±15,0
6 mM NaAc + CF	11,4±0,9	66,9±0,3	69,5±3,6
12 mM NaAc + CF	10,9±1,7	72,6±7,2	48,6±23,4

**Fig. 1 (abstract P-512).**
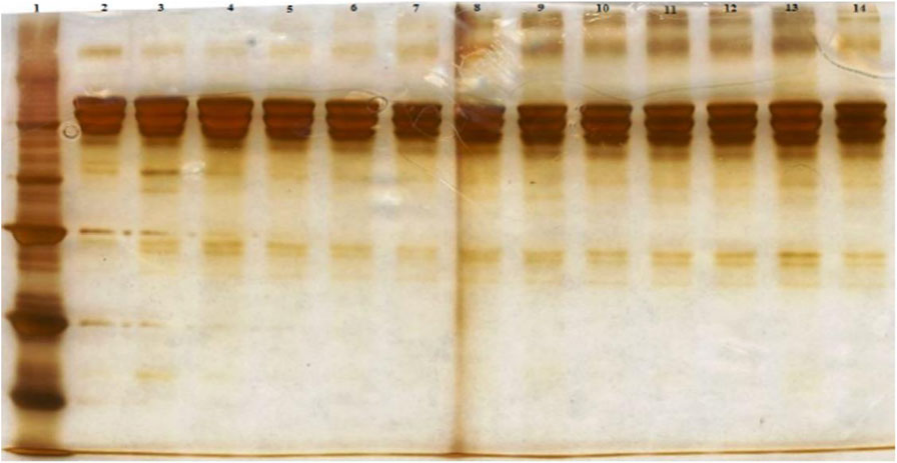
Non-reduced SDS-PAGE of purified mAb. Lane 1: Weight marker, Lane 2: Originator, Lanes 3/4: Control, Lanes 5/6: 6 mM NaAc, Lanes 7/8: 12 mM NaAc, Lanes 9/10: Control + CF, Lanes 11/12: 6 mM NaAc/CF, Lanes 13/14: 12 mM NaAc/CF

### P-513 Expression of full-length shark-derived antibody by CHO cells

#### Hajime Enatsu^1^, Motoki Arinaga^1^, Nako Okamoto^1^, Noriko Yamano-Adachi^1^, Yuichi Koga^1^, Takeshi Omasa^1, 2^

##### ^1^Graduate school of Engineering, Osaka University, Suita, Osaka, 560-0871, Japan; ^2^Manufacturing Technology Association of Biologics, Kobe, 650-0047, Japan

###### **Correspondence:** Hajime Enatsu (omasa@bio.eng.osaka-u.ac.jp)

**Background**

Mammalian-derived immunoglobulins are used widely for scientific research and clinical therapy. However, IgG often denatures in non-physiological environments [1], which limits the use of IgG for diagnostic tests under conditions of high temperature and extreme pH. The shark-derived immunoglobulin new antigen receptor (IgNAR) possesses bioactivity in urea-containing shark blood, and has therefore received increasing attention because of its stability. IgNAR consists of one variable domain and five constant domains (C1–C5) [3]. Stability related motifs of IgNAR were identified previously [4] and proved to be key factors for improving the thermostability of the IgG. Nevertheless, the structure and the physical characteristics of the full-length IgNAR are unknown. The biggest issue is that the C5 domain, which is predicted to contain complex post-translational modification sites such as intermolecular disulfide bonds and N-linked glycosylation sites, cannot fold correctly and dimerize when recombinantly expressed by *E. coli* and insect cells. In this report, we stably produced full-length IgNAR using Chinese hamster ovary (CHO) cells, which are used widely for the expression of IgG antibodies. Moreover, the expression construct of His-tagged IgNAR simplified the purification process, which should facilitate　structural analysis of IgNAR.

**Materials and methods**

The expression vector was designed to express C-terminal His-tagged full-length IgNAR (IgNAR-His) and the N-terminal His-tagged C5 (C5-His) single domain. CHO-K1 cells were stably transfected with the vector and selected with puromycin. Generated stable cells, adapted for growth in suspension, were cultivated in BalanCD CHO Growth A medium. The supernatant of the IgNAR-His and C5-His stable cell culture was purified by nickel affinity chromatography. Purified IgNAR-His was analyzed by SDS-PAGE (12% gel). Purified C5-His was deglycosylated with glycopeptidase F and analyzed by Tricine SDS-PAGE (12% gel).

**Results**

SDS-PAGE analysis showed that elution of the loaded IgNAR-His stable cell culture supernatant with 200 mM imidazole gave three bands with molecular weights of approximately 95, 55, and 36 kDa (Figure 1A). In contrast, no band was observed in the corresponding lane loaded with the non-transfected CHO-K1 cell culture control.

A band representing the C5 domain was observed in the Tricine SDS-PAGE (Figure 1B). In addition, the band representing the C5 domain shifted following deglycosylation. Moreover, the deglycosylated C5 domain had a two-fold higher molecular weight under non-reducing conditions when compared with that under reducing conditions.

**Conclusions**

In this report, we generated a full-length IgNAR producing CHO cell line. Moreover, use of nickel affinity chromatography successfully isolated IgNAR from the cell culture, while the elution fraction still contained fragments of IgNAR-His. In addition, although the IgNAR C5 domain misfolds and undimerizes in other expression hosts, our results indicated that CHO cells expressed the recombinant C5 domain as a dimer formed by intermolecular disulfide bonds and as an N-glycosylated form. However, further purification steps are necessary before actual analysis of the folded state of the C5 domain can be conducted, as well as structural and the physiological analysis of the full-length IgNAR. The results of this study indicate that CHO cells produced full-length IgNAR in its native fold.

**Acknowledgments**

This research was partially supported by the developing key technologies for discovering and manufacturing pharmaceuticals used for next-generation treatments (Japan Agency for Medical Research and Development (AMED) under Grant Numbers JP17ae0101003, JP18ae0101056, 57, 58 and 66) and MEXT/JSPS KAKENHI Grant Number JP17H06157..

**References**

1. Matz H, Dooley H. Shark IgNAR-derived binding domains as potential diagnostic and therapeutic agents. Dev Comp Immunol. 2019; 90:100-107.

2. Rou KH, Greenberg AS, Greene L, Strelets L, Avila D, Mckinney EC, Flajnik MF. Structural analysis of the nurse shark (new) antigen receptor (NAR): Molecular convergence of NAR and unusual mammalian immunoglobulins. Proc Natl Acad Sci USA. 1998; 95:11804-11809.

3. Feige MJ, Gräwert MA, Marcinowski M, Hennig J, Behnke J, Ausländer D, Herold EM, Peschek J, Castro CD, Flajnk M, Hendershot LM, Sattler M, Groll M, Buchner J.The structural analysis of shark IgNAR antibodies reveals evolutionary principles of immunoglobulins. Proc Natl Aad Sci USA. 2014; 111: 8155-8160.

**Fig. 1 (abstract P-513).**
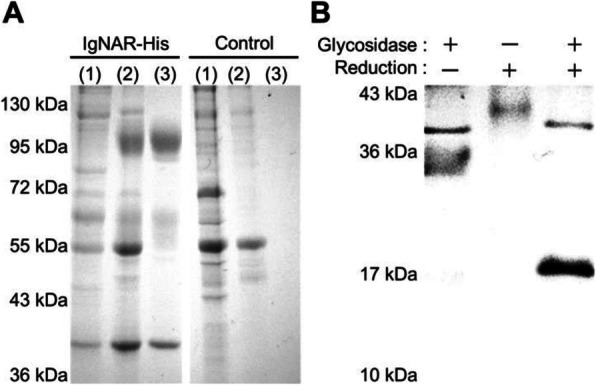
**A** Analysis of IgNAR-His expression and purification by SDS-PAGE with a purified non-transfected CHO cell culture control. Imidazole concentrations used for elution: (1)50 mM, (2)100 mM and (3)200 mM. **B** Analysis of C5-His dimerization and N-glycosylation by Tricine SDS-PAGE

### P-517 Novel approach to account for enzyme regulation in kinetic models of protein glycosylation

#### Pavlos Kotidis^1^, Ioscani Jimenez del Val^2^, Frederick J. Krambeck^3,4^, Michael J. Betenbaugh^4^, Cleo Kontoravdi^1^

##### ^1^Centre for Process Systems Engineering, Department of Chemical Engineering, Imperial College London, London SW7 2AZ, UK; ^2^School of Chemical & Bioprocess Engineering, University College Dublin, Ireland; ^3^ReacTech Inc., Alexandria, VA, USA; ^4^Department of Chemical and Biomolecular Engineering, John Hopkins University, Baltimore, MD, USA

###### **Correspondence:** Pavlos Kotidis (cleo.kontoravdi@imperial.ac.uk)

**Background**

The quality of pharmaceutical glycoproteins, including monoclonal antibodies (mAbs), is greatly influenced by N-linked glycosylation [1]. While several experimental strategies have been implemented to control protein glycosylation, mathematical models have also been used to describe and control the produced glycoform *in silico* [2-4]. Among mathematical models, kinetic models, although often detailed and mechanistic, are usually limited to specific operating conditions because of the inability to predict *a priori* regulation over enzyme expression in response to changes in the cellular environment [5, 6]. In the present work, a modelling framework describing the N-linked glycosylation of a mAb product and host cell proteins (HCPs) is proposed, in order to account for nucleotide sugar donor (NSD) consumption for HCP glycosylation and enzyme regulation events and, ultimately, improve model predictions compared to previously developed models [2].

**Materials and methods**

Model construction, simulation, parameter estimation and optimization were conducted in gPROMS v.5.1.1 (Process System Enterprise Ltd, London, U.K., www.psenterprise.com/gproms). The GLYMMER^TM^ software (ReacTech Inc., USA, https://www.reactech.net/) was utilized for network construction, extraction of stoichiometric tables for model automation and estimation of enzymes concentration.

A broad network of ~15,000 oligosaccharides and ~50,000 reactions was generated in GLYMMER [7] by assigning the substrates upon which the 13 glycosylation enzymes act. The network was subsequently reduced to include only the essential reactions for the formation of the previously reported HCP glycoprofile with a distribution of >1% [8]. The modelling framework was trained to simultaneously fit the HCP & mAb glycoform distribution. Model training to experimental data of fed-batch CHO cell cultures with the addition of galactose and uridine [2] included the estimation of glycosylation enzymes concentration and selected dissociation constants.

**Results**

Addition of galactose and uridine in CHO cell cultures has been shown to lead to β-1,4-galactosyltransferase (GalT) upregulation (1.25x-2x) and increased galactosylation of mAbs and HCPs [5, 6]. A previously presented kinetic cell culture model was used to estimate intracellular NSD concentrations for a fed-batch experiment in which galactose and uridine were also added [2]. These concentrations, along with the mAb glycoform distribution for the same experiment and a previously reported distribution for CHO HCPs were fed to GLYMMER^TM^, which predicted the GalT upregulation within each interval, as presented in Figure 1. GalT upregulation, ranging from 1.27x to 1.58x, was subsequently introduced to the model and resulted in accurate prediction of mAb glycoform distribution in a galactose and uridine feeding experiment within a 3% error range.

**Conclusions**

The kinetic cell culture model was successfully coupled with the GLYMMER^TM^ software to account for GalT upregulation and HCP glycosylation, resulting in a <3% error - in the prediction of mAb glycosylation for different time points of a fed-batch CHO cell culture that included the addition of galactose and uridine, improving that way the predictive capabilities of a previous developed holistic modelling framework. The workflow and model can be used to account for the regulation of other glycosylation enzymes (α-1,6-fucosyltransferase, α-1,6-sialyltransferase etc.) that have a great impact on protein glycoprofile, therefore increasing model applicability to ranges of experimental conditions for which it was not previously calibrated.

**Acknowledgements**

P.K. gratefully acknowledges his funding from the PhD scholarship scheme of the Department of Chemical Engineering, Imperial College London.

**References**

1. Solá, R.J. and K. Griebenow, Effects of glycosylation on the stability of protein pharmaceuticals. Journal of pharmaceutical sciences, 2009. 98(4): p. 1223-1245.

2. Kotidis, P., et al., Model-based optimization of antibody galactosylation in CHO cell culture. Biotechnology and Bioengineering, 2019. 116(7): p. 1612-1626.

3. Krambeck, F.J. and M.J. Betenbaugh, A mathematical model of N-linked glycosylation. Biotechnology and Bioengineering, 2005. 92(6): p. 711-728.

4. Jimenez del Val, I., J.M. Nagy, and C. Kontoravdi, A dynamic mathematical model for monoclonal antibody N-linked glycosylation and nucleotide sugar donor transport within a maturing Golgi apparatus. Biotechnology Progress, 2011. 27(6): p. 1730-1743.

5. Grainger, R.K. and D.C. James, CHO cell line specific prediction and control of recombinant monoclonal antibody N-glycosylation. Biotechnology and Bioengineering, 2013. 110(11): p. 2970-2983.

6. Wong, N.S.C., et al., An investigation of intracellular glycosylation activities in CHO cells: Effects of nucleotide sugar precursor feeding. Biotechnology and Bioengineering, 2010. 107(2): p. 321-336.

7. Krambeck, F.J., et al., Model-based analysis of N-glycosylation in Chinese hamster ovary cells. PLOS ONE, 2017. 12(5): p. e0175376.

8. North, S.J., et al., Glycomics profiling of Chinese hamster ovary cell glycosylation mutants reveals N-glycans of a novel size and complexity. The Journal of biological chemistry, 2010. 285(8): p. 5759-5775.

**Fig. 1 (abstract P-517).**
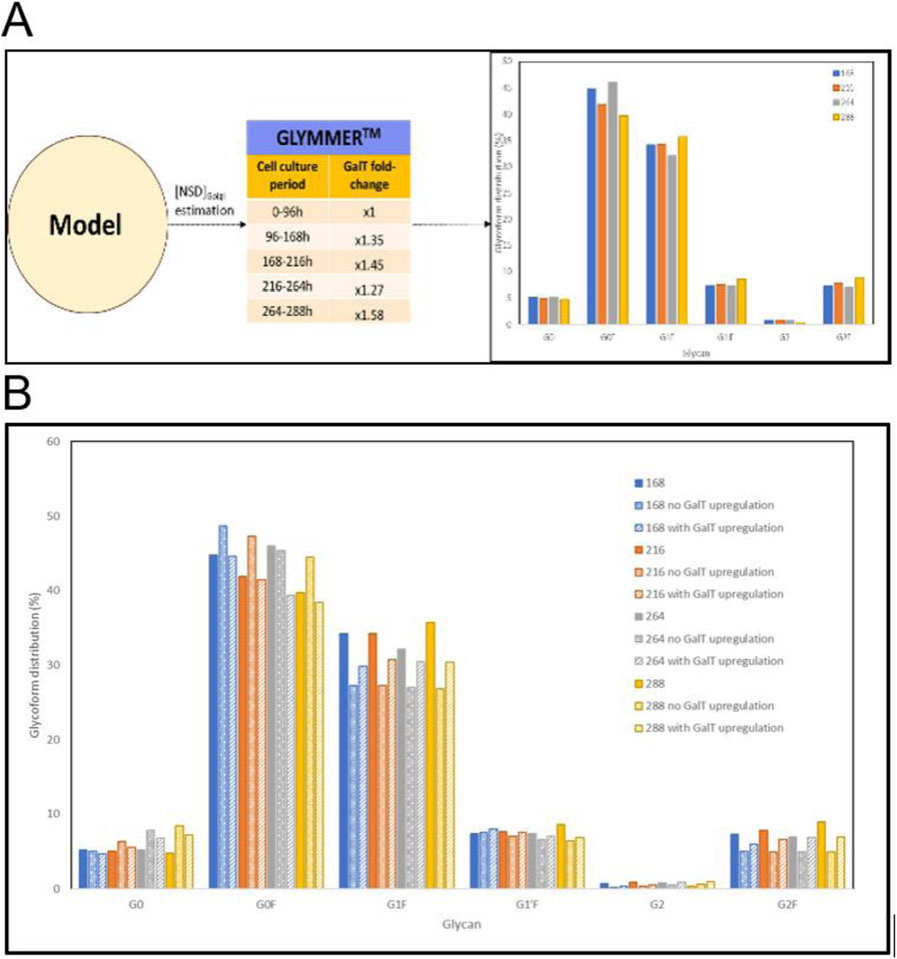
(A) Workflow: estimated concentration of NSDs is fed to GLYMMER for the estimation of GalT upregulation that is fed back to the model for the estimation of the glycoprofile. (B) Simulation fitting with (following the described workflow) and without considering the GalT upregulation

### P-530 Evaluation of the cell culture process robustness and cell line stability

#### Belén Bosco, Ignacio Amadeo, Laura Mauro, Romina Zuqueli, Guillermina Forno

##### Zelltek S.A. Santa Fe, Argentina. RN 168 – Paraje El Pozo Parque Tecnológico del Litoral Centro (3000)

###### **Correspondence:** Belén Bosco (mbbosco@zelltek.com.ar)

**Background**

Robustness is a measure of the reproducibility of a process under the variation in conditions normally expected from laboratory to laboratory and from analyst to analyst [1,2].

Cell lines used for the production of biopharmaceuticals must be able to maintain the productivity and quality of the protein through numerous processes of cell duplication, within certain parameters, at productive scales [3,4].

**Materials and methods**

Controlled cultures were established in 4.5 L bioreactors in perfusion mode. Obtained culture supernatants were purified and the obtained material quality was analyzed. Samples were taken daily and cell density, viability and productivity were measured.

In order to analyze the robustness of the production process, a 2‑Level Factorial design with two numeric factors, three levels, with two center points was employed.

Statistical analysis: model fitting and comparison with Design-Expert software.

The stability of the cell line was analyzed by subculturing the WCB for 80 generations (G80). Two fermentations were carried out from the aged WCB and compared to six conventional runs (G0). Statistical analysis, quality range criteria: if all G80 values fell within the MEDIA_G0_ ± 3 SD_G0_ then it was considered that the WCB was stable for at least 80 generations.

**Results**

**Cell culture process robustness**

As it can be seen in Figure 1A, the bioreactor fed with 1.6 g/l Glc and 0.5 RV/day showed slower growth than the central point one. On the other hand, fermentations fed at 1.5 RV/day displayed higher maximum cell densities (Table 1).

Regarding qProd, the fermenter fed with 1.6 g/l Glc and 0.5 RV/day showed a higher qProd than the central point one. On the other hand, the bioreactor fed with Glc 0.6 g/l and 0.5 RV/day presented lower qProd than the conventional fermentation. Viability values were similar for all runs.

Specific growth rate (*μ*), average Specific production rate (qProd), Productivity and Product mass were compared statistically using the software Design-Expert. Results are shown in Table 2 and Figure 1B. Chromatographic yields obtained from the downstream processing were compared and no statistical significant differences were detected (ANOVA, p > 0.05). The purified material quality was analyzed (Protein glycosylation, *in vitro* potency, HCP and oligomers content) and no statistical significant differences were detected.

**Cell line stability**

Statistical analysis showed no statistically significant differences between cell densities, viabilities and μ values for G0 and G80 (Figure 1C). Also, concentration of the product in the culture supernatant and qProd were similar for G0 and G80. There were no statistically significant differences between concentration of the protein in the culture supernatant, qProd and productivity after the four-step purification, at API level. The purified material quality was analyzed (N-glycans and sialic acid contents, isoforms profile, *in vitro* potency and HCP contents) and no statistical significant differences were detected.

**Conclusions**

Perfusion rate and glucose in cell culture medium did not affect product quality in all the design of space, since protein glycosylation, potency, host cell proteins and oligomers content complied with the specifications. However, maximum cell density, growth rate and purified Product mass after the fermentation run were significantly affected by changes in those operative parameters.

These studies suggest that the design of experiments applied to cell culture processes has the potential to demonstrate robustness and significantly increase productivity through process optimization.

Cell growth, productivity and quality of API batches obtained from WCB G0 and G80 were measured and compared. Cell density, viability, Product concentration and qProd were similar for all 8 fermentation runs.

Also, protein glycosylation, *in vitro* potency and HCP contents were compared and no statistical significant differences were detected. Finally, it is concluded that the WCB can be considered stable during at least 80 generations.

**References**

1. Rathore, Sofer. Process Validation in Manufacturing of Biopharmaceuticals. In: Taylor & Francis Group, editors. Guidelines, Current Practices, and Industrial Case Studies. USA; 2005. p. 47-66.

2. Lynn. Pharmaceutical and Medical Device Validation by Experimental Design. In: Torbech, editors. Informa Healthcare. USA; 2007. p.119-140.

3. Li, Vijayasankaran, Shen, Kiss, Amanullah. Cell culture processes for monoclonal antibody production. mAbs. 2010; 2(5):466-477.

4. Kim, O’Callaghan, Droms, James. A Mechanistic Understanding of Production Instability in CHO Cell Lines Expressing Recombinant Monoclonal Antibodies. Biotechnology and Bioengineering. 2011; 108(10):2434-2446.

**Table 1 (abstract P-530).** Results obtained from the robustness study fermentations

Glc (g/l)	Perfusion (RV/day)	μ (day^**-1**^)	Average qProd (pg/cell/day)
1.1	1.0	0.4408	7.18
1.1	1.0	0.4135	9.21
1.6	0.5	0.2915	10.34
0.6	1.5	0.3445	8.43
0.6	0.5	0.3915	4.88
1.6	1.5	0.4085	6.15

**Table 2 (abstract P-530).** Design-Expert statistical comparison results

Parameter	Fitted model	Factors that show significant effects
μ	Quadratic	Interaction Perfusion-Glc
qProd	2FI	Interaction Perfusion-Glc
Productivity^1^	2FI	None
Product mass^2^	Linear	Perfusion

^1^Purified Product mass per liter of processed culture supernatant.

^2^Purified product mass obtained at the end of the fermentation.

**Fig. 1 (abstract P-530).**
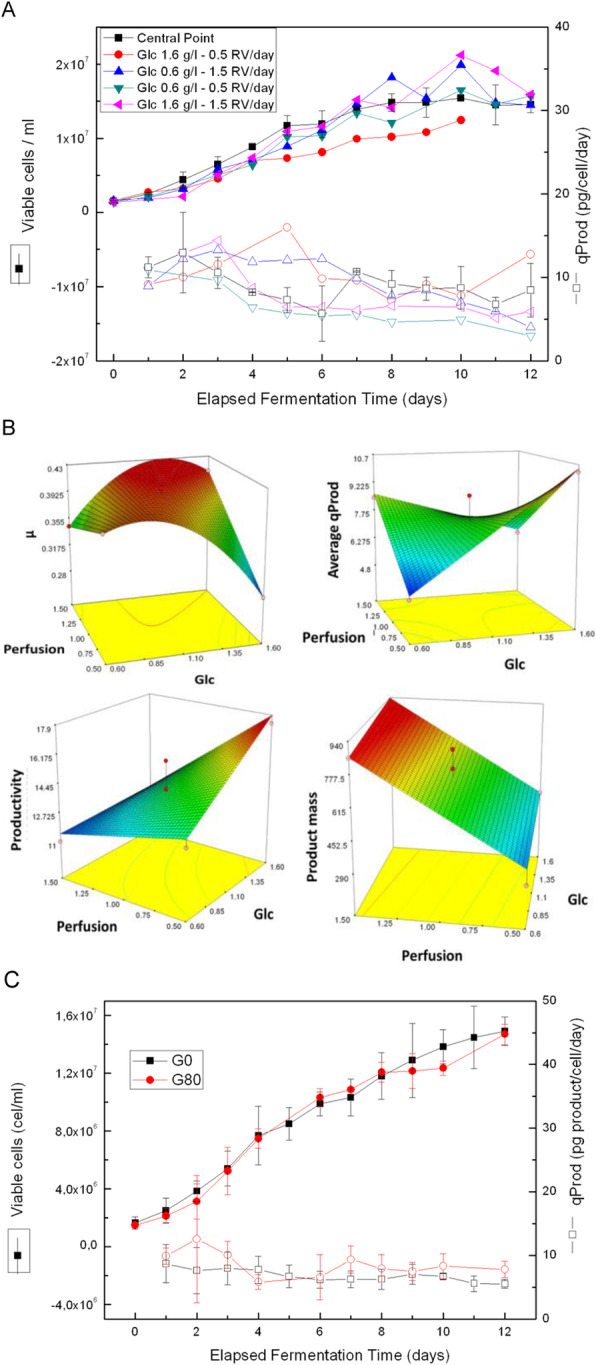
A) Viable cells concentration and qProd results obtained from the robustness study fermentations. B) Statistical analysis of μ, qProd, Productivity and Product mass results regarding Glc concentration and Perfusion rate. C) Viable cells concentration and qProd results obtained from G0 and G80 fermentations

### P-534 Study of a new cell culture medium component to reduce product microheterogeneity

#### Valentine Chevallier^1,2^, Mikael Rørdam Andersen^2^, Laetitia Malphettes^1^

##### ^1^UCB Pharma S.A., Upstream Process Sciences, Braine l’Alleud, Belgium; ^2^Technical University of Denmark, Department of Biotechnology and Biomedicine, Kgs. Lyngby, Denmark

###### **Correspondence:** Valentine Chevallier

**Background**

To provide high-quality product to patients, product variants have to be controlled during biopharmaceutical manufacturing. Controlling microheterogeneity may limit process yields. During the development of an IgG production process, it has been demonstrated that a reduction of cysteine was helpful to reduce charge variants. However this reduction alone also led to a pre-harvest titer reduction. Analogs of cysteine have already been used in cell culture processes to improve its stability - S-Sulfocysteine for example or to increase productivity thanks to their antioxidant properties such as N-Acetyl-Cysteine [1,2]. However cystine analogs have not yet been used in the context of recombinant proteins production for biopharmaceutical [3,4]. In this presentation, cysteine and cystine analogs are evaluated as a potential replacement of a varying percentage of the cysteine/cystine added in the production process and for their impact on the microheterogeneity of a model monoclonal antibody and its pre-harvest titer.

**Materials and methods**

CHO-DG44 cells producing a monoclonal antibody (Mab) were inoculated in 12mL of basal media at a seeding density of 0.35x106 cells /mL in Ambr15 bioreactors (Sartorius). The cells were cultivated during 14 days in fed-batch mode. Cysteine in the feed was replaced by different cysteine and cystine analogs at different percentages (molar equivalence). In previous experiment, S-sulfocysteine has been shown to be toxic for the cell line used (Data not shown). In this context, three cystine / cysteine analogs has been tested : N,N′-Diacetyl-L-Cystine Dimethylester (DACDM), N,N’-Diacetyl-L-Cystine (DiNac), N-Acetyl-L-Cysteine (Nac). At the end of the 14 days of culture, the cell culture fluid was harvested by centrifugation. The supernatant was then purified using a protein-A affinity chromatography (GE). The protein-A eluates were used for the charge variants determination using isocapillary focusing (ProteinSimple). In a second time, cells have been cultivated in shake flask with 50%cysteine 50% DACDM feed to generate samples for intracellular glutathione analysis by mass spectrometry (Waters).

**Results**

*Replacement of 50% cystine by DACDM reduces product microheterogeneity without affecting titer*

Figure 1 represents the response to different cysteine/cystine replacement level of Nac, DiNac or DACDM, in main charge species level and titer. Only 5% increase of main charge species was observed with 70% of N-acetyl-cystine without detrimental effect on titer. For N,N’-diacetyl-L-cystine, the main charge species level increased with the increase of the percentage of replacement to reach a 12.5% positive difference with respect to the control. However, this increase was associated to a titer decrease. The optimum main charge species level with N,N’-diacetyl-L-cystine dimethylester was obtained between 50% and 70% of cysteine/cystine replacement. Moreover, 50% of replacement did not affect product titer.

*DACDM increases intracellular glutathione content*

Total intracellular glutathione results from the shake flask cultivation are shown in Table 1. Cells fed with the feed containing DACDM had an higher glutathione content.

**Conclusion**

Thanks to a new cell culture medium component, the DACDM, we have reduced the recombinant protein heterogeneity without altering the process performance. In addition to charge variants, we have also demonstrated in another study, that this molecules can decrease Mab coloration. The mechanism of action of DACDM is not fully understood but it seems to be related to the glutathione metabolism.

**Acknowledgements**

This worked was supported by UCB Pharma and in part by the Innovation fund of Denmark (Grant case n°5189-00037B). We would like to thank Marvin Zoller for his help in the lab for glutathione measurement**.**

**References**

1. Hecklau, C, Pering S, Seibel R, Schnellbaecher A, Wehsling M, Eichhorn T, Hagen J and Zimmer A. S-Sulfocysteine simplifies fed-batch processes and increases the CHO specific productivity via anti-oxidant activity. J Biotechnol. 2016; 218: 53-63.

2. Oh HK, So MK, Yang J, Yoon HC, Ahn JS, Lee JM, Kim JT, Yoo JU and Byun TH. Effect f N-Acetylcysteine on butyrate-treated Chinese hamster ovary cells to improve the production of recombinant human interferon-beta-1a. Biotechnol Prog. 2005; 21(4): 1154-1164.

3 .Kitazawa M, NakanoT, Chuujou H, Shiojiri E, Iwasaki K and Sakamoto K. Intracellular redox regulation by a cystine derivative suppresses UV-induced NF-κB activation. FEBS Letters. 2002; 526(1–3): 106-110.

4. Pettersson, KS, Eliasson UB, Abrahamsson T, Wagberg M, Carrier M and Kengatharan KM. N,N-diacetyl-L-cystine improves endothelial function in atherosclerotic Watanabe heritable hyperlipidaemic rabbits.Basic Clin Pharmacol Toxicol. 2007; 100(1): 36-42.

**Fig. 1 (abstract P-534).**
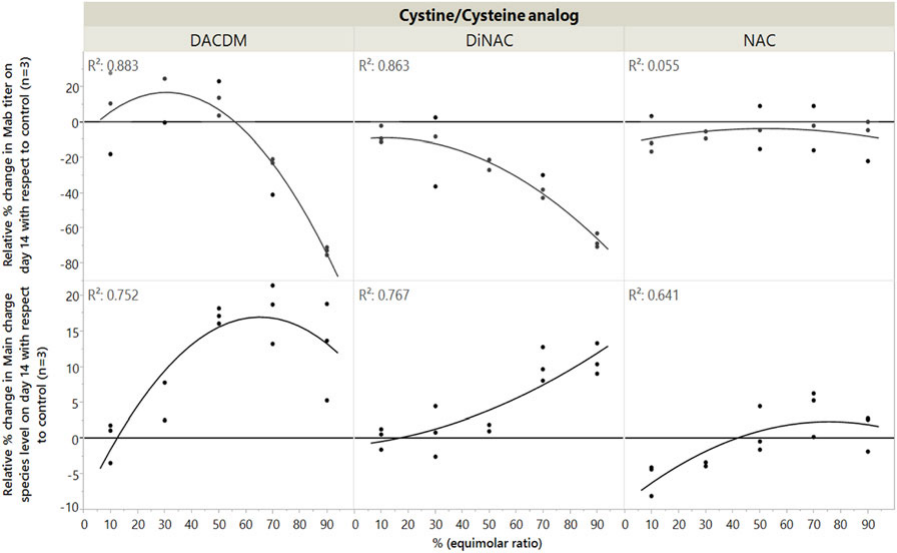
Relative percentage change in Mab titer and main charge species level on day 14 with respect to control at different cysteine replacement ratio in the feed. The cysteine and cystine analogs tested were : N,N′-Diacetyl-L-Cystine Dimethylester (DACDM), N,N’-Diacetyl-L-Cystine (DiNac) and N-Acetyl-L-Cysteine (Nac)

**Table 1 (abstract P-534).** Total intracellular glutathione concentration at day 14

Condition	Total intracellular glutathione (μmol/ 10^**7**^ cells)
Control (n=2)	0.46
50% Cysteine 50% DACDM feed (n=2)	0.60

### P-536 Compaction of raw materials and cell culture media

#### Corinna Merkel, Aline Zimmer, Anke Simon

##### Merck Millipore, Pharm-Chemical Solutions, Research & Development Upstream, Darmstadt, Germany

###### **Correspondence:** Corinna Merkel (corinna.merkel@milliporesigma.com)

**Background**

Bulk powders like cell culture media (CCM) or single chemicals show physical disadvantages. Very fine CCM powders lead to high dust formation and have a poor flowability. In addition, dissolution is time consuming due to floating of light particles on the water surface. For some single raw materials, appropriate handling is often impeded due to caking of the bulk material. Strong mechanical forces are needed to break up the material. In many cases, these limitations of powders can be overcome by granulated material.

**Materials and methods**

Bulk powders were granulated by a dry roller compactor RC-120 from Powtech. During dry roller compaction, no water or excipient is added to the process. The technology works with compression force only. Main process parameters influencing the final granulated material are bulk composition (fluffy, sticky, crystalline), compression force (stability), grid size at milling step (particle size distribution)

**Results**

Granules of CCM showed reduced dissolution times compared to powder. Fine CCM powder floated on the water surface prolonging dissolution time due to inefficient wetting of powder. Granules sedimented to the impeller instantaneously. In addition, dust formation during handling of CCM was significantly minimized, increasing safety for employees and decreasing contamination risk and cleaning costs. Better handling of granules (Carr Indices 10-16 = good flowability) was achieved due to improved flowability compared to powder (Carr Indices 25-35 = non-satisfactory flowability). Moreover, storage and transport costs can be reduced by granules due to bulk volume reduction up to 40%.

Homogeneity of CCM components within granules was confirmed for amino acids, vitamins and trace elements. Integrity of labile CCM components like vitamins and proteins (e.g. insulin) in granules was compared to the bulk powder and no differences were observed. This confirmed that the applied compression force did not damage CCM components.

Compaction of CCM powder did not impact performance of CHO cells. Viable cell density, viability and produced monoclonal antibody did not differ between granules and powder.

Granules of single chemicals resulted in improved flowability and reduced caking compared to the bulk material. Stability studies at ambient and accelerated (45 °C and 75% rel. humidity) conditions showed that powders formed solid monoblocs whereas granules kept free flowing without critical caking issues up to nine months. Dissolution times between granules and bulk material of single chemicals were similar.

**Conclusions**

Granulated CCM or single chemicals result in improved process efficiency (Figure 1).

**Fig. 1 (abstract P-536).**
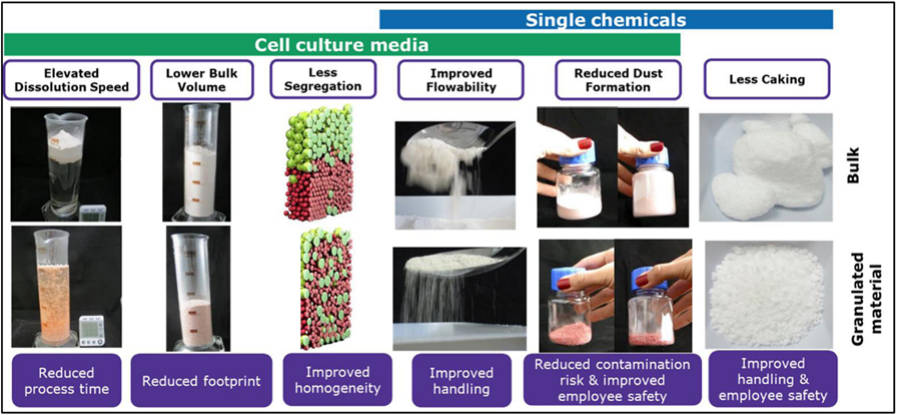
Advantages of granules

### P-538 Impact of acetylated and non-acetylated fucose analogues on IgG glycosylation

#### Martina Zimmermann^1,2^, Janike Ehret^1^ and Aline Zimmer^1^

##### ^1^Merck Life Sciences, Upstream R&D, Frankfurter Strasse 250, 64293 Darmstadt, Germany; ^2^Institute for Organic Chemistry and Biochemistry, Technische Universität Darmstadt, Alarich‑Weiss‑Strasse 4, 64287 Darmstadt, Germany

###### **Correspondence:** Martina Zimmermann (martina.zimmermann@external.merckgroup.com)

**Background**

The biological activity of therapeutic antibodies is highly influenced by their glycosylation profile [1]. A valuable method to increase the cytotoxic efficacy of antibodies, which are used, for example, in cancer treatment, is the reduction of core fucosylation, as this enhances the elimination of target cells through antibody-dependent cell-mediated cytotoxicity [2]. Development of fucose analogues is currently the most promising strategy to reduce core fucosylation without cell line engineering. Since peracetylated sugars display enhanced cell permeability over the highly polar free hydroxy sugars, this work sought to compare the efficacy of peracetylated sugars to their unprotected forms [3].

**Material and methods**

Fed-batch experiments were performed in spin tubes with a CHO K1 cell line expressing a recombinant IgG1. Compounds to modulate the fucosylation level were added into the cell culture feed. Viable cell density, viability and titer were assessed during a fed-batch experiment and the glycosylation profile was analyzed on day 7, 10 and 12 with ultra-performance liquid chromatography coupled to a mass spectrometer.

**Results**

Two potent fucose analogues, 2-deoxy-2-fluorofucose (2F-Fuc) and 5-alkynylfucose (5-AlkFuc), and their acetylated forms were compared for their effects on fucosylation. Incorporation of the fucose analogues was confirmed through mass spectrometry data. 5-alkynylfucose proved to be more potent than 2-deoxy-2-fluorofucose at reducing core fucosylation but was associated with a significant higher incorporation of the alkynylated fucose analogue. Acetylation of the sugar yielded only slightly lower fucosylation levels. Increasing inhibitor concentrations reduced further the impact of acetylation suggesting that acetylation has a minor impact on cellular entry.

**Conclusion**

The general low impact of acetylation detected in this study may be explained by differences in the mechanism of cellular transport when comparing fucose and other carbohydrates. In the literature, acetylation of ManNAc and a glucosamine derivative was reported to enhance the passive diffusion [4, 5]. For ManNAc, no transporter has been described so far and such a transporter might not be essential for cells since ManNAc is endogenously produced using glucose as precursor [4]. In contrast, fucose as well as the analogues may be transported in cells via a transporter. The sodium/myo-inositol transporter slc5a3 is expressed in CHO cells and was reported to transport L-Fucose due to the high structural similarity to myo-inositol [6, 7]. The hypothesis that fucose derivatives can be recognized by the transporter is further supported by the fact that fucose analogues are also recognized by other key enzymes in the fucosylation pathway.

Overall, chemical modification of the fucose structure is a powerful method to reduce core fucosylation without cell line engineering, but acetylation might not be necessary to yield high afucosylation level.pt?>

**References**

1. Abès R and Teillaud J-L, Impact of Glycosylation on Effector Functions of Therapeutic IgG. *Pharmaceuticals* 2010, 3, 146-57.
2. Gerdes CA, Nicolini VG, Herter S, van Puijenbroek E, Lang S, Roemmele M, Moessner E, Freytag O, Friess T, Ries CH, Bossenmaier B, Mueller HJ, and Umana P, GA201 (RG7160): a novel, humanized, glycoengineered anti-EGFR antibody with enhanced ADCC and superior in vivo efficacy compared with cetuximab. *Clinical Cancer Research* 2013, 19, 1126-38.
3. Ramsden MJ, Blake FSR, and Fey NJ, The effect of acetylation on the mechanical properties, hydrophobicity, and dimensional stability of Pinus sylvestris. *Wood Sci. Technol.* 1997, 31, 97-104.
4. Gilormini PA, Lion C, Vicogne D, Levade T, Potelle S, Mariller C, Guerardel Y, Biot C, and Foulquier F, A sequential bioorthogonal dual strategy: ManNAl and SiaNAl as distinct tools to unravel sialic acid metabolic pathways. *Chem Commun (Camb)* 2016, 52, 2318-21.
5. Zaro BW, Chuh KN, and Pratt MR, Chemical reporter for visualizing metabolic cross-talk between carbohydrate metabolism and protein modification. *ACS chemical biology* 2014, 9, 1991-1996.
6. Schneider S, Inositol transport proteins. *FEBS Lett* 2015, 589, 1049-58.
7. Chinese hamster genome database: http://www.chogenome.org.

**Fig. 1 (abstract P-538).**
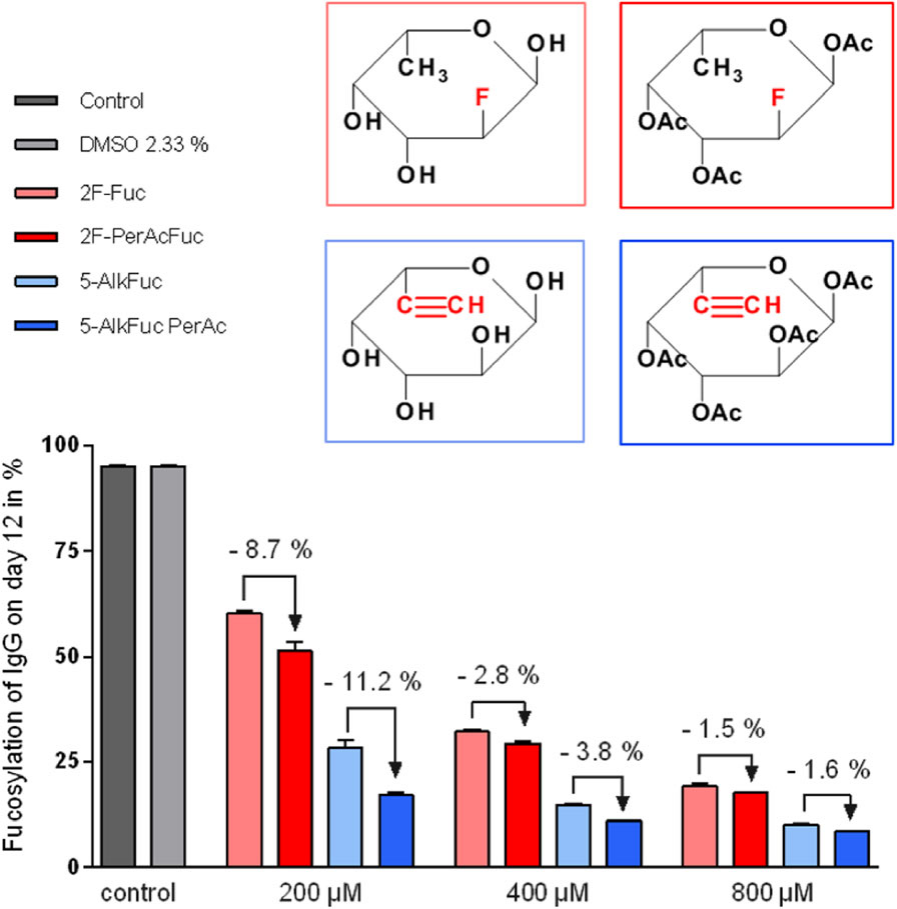
IgG fucosylation on day 12 using two fucose analogues and the respective acetylated compounds (n=2)

### P-544 ATPS phase separation for integrated clarification and purification

#### Thomas Kruse^1,2^, Axel Schmidt², Markus Kampmann^1^, Jochen Strube²

##### ^1^Sartorius Stedim Biotech GmbH, Göttingen, 37079, Germany; ^2^Institute for Separation and Process Technology, Clausthal University of Technology, Clausthal-Zellerfeld, 38678, Germany

###### **Correspondence:** Thomas Kruse (thomas.kruse@sartorius.com)

**Background**

Intensified upstream processes for the production of monoclonal antibodies (mAb) have resulted in high challenges for the subsequent clarification and capture operations in the downstream process [1]. A promising approach to meet these challenges might be a selective mAb extraction via an aqueous two-phase system (ATPS) directly from the cultivation broth. Biomolecules show a different distribution between the two phases of an ATPS. The distribution is dependent on the ATPS (e.g. PEG concentration and molecular weight, pH value, salt concentration) as well as properties of the molecule itself (e.g. charge, hydrophobicity, size, conformation and affinities to the phases) [2].

During the downstream process the mAb has to be separated from process related impurities like host cell proteins (HCP), deoxyribonucleic acid (DNA) and cells. The objective of this study is an integration of a first capture and purification step by an ATPS together with a clarification and sterile filtration by a selective membrane based phase separation. The main challenge for a selective phase separation was the high physico-chemical similarity of both ATPS phases, which was addressed by a membrane modification.

**Materials and methods**

Four different ATPS, which have been reported for mAb purification, with different phase forming components and compositions as well as an optimized ATPS (based on a DoE approach) were examined with cell containing cultivation broth as ATPS feed component (Table 1).

For modification a hydrophobic polypropylene membrane (approximately 0.4 μm mean pore diameter) was incubated overnight in the polymer rich light phase (LP) of the respective ATPS supplemented with a surfactant (Tween20). The used surfactant concentration (1 w%) was a trade-off between surfactant solubility in the LP and membrane wettability by the solution.

Both ATPS phases overflow the modified membrane. Only the LP, containing the mAb flow through the membrane while the salt-rich heavy phase (HP), containing the majority of the impurities, is retained. Clarification is ensured by the narrow mean membrane pore diameter (0.4 μm) of the used membrane.

**Results**

In order to select a suitable ATPS the mAb yield and purity was determined by equilibrium experiments for the different ATPS (Table 2).

The yield of mAb was highest for ATPS1 and ATPS Opt, while DNA as well as HCP removal was significantly increased for ATPS Opt. Due to these results focus was placed on ATPS Opt.

A new developed phase separator, with 800 cm² membrane area, was used for the membrane based phase separation (Figure 1). Throughout the whole experiment a breakthrough of the HP did not occur, resulting in a permeate phase purity of 100 %. A flush with 30 % LP (water as feed component) was executed to increase mAb recovery.

**Conclusions**

Flow through of the target phase (LP) was realized by membrane modification to achieve a selective membrane based ATPS phase separation. The ATPS optimized by a DoE approach showed the highest values for mAb yield and purity in equilibrium experiments compared to different model ATPS. An integration of clarification by membrane based phase separation with a first capture and purification step by ATPS was accomplished with an optimized ATPS, offering great potential for further process intensification.

**Acknowledgements**

Thanks goes to the complete BioProcessing and Separation Technology team of Sartorius Stedim Biotech, Göttingen and the Institute for Separation and Process Technology, Clausthal University of Technology. This research was partially supported by a Federal Ministry of Economics-funded project (TPTP-Traceless Plant Traceless Production).

**References**

1. Singh N, Arunkumar A, Chollangi S, Tan ZG, Borys M, Li ZJ. Clarification technologies for monoclonal antibody manufacturing processes. Biotechnol. Bioeng. 2016; 133: 698-716.

2. Iqbal M, Tao Y, Xie S, Zhu Y, Chen D, Wang X, Huang L, Peng D, Sattar A, Shabbir MAB, Hussain HI, Ahmed S, Yuan Z. Aqueous two-phase system (ATPS): An overview and advances in its applications. Biol. Proced. Online. 2016;

18: 18.

3. Schmidt A, Richter M, Rudolph F, Strube J. Integration of Aqueous Two-Phase Extraction as Cell Harvest and Capture Operation in the Manufacturing Process of Monoclonal Antibodies. Antibodies. 2017; 6: 21.

4. Oelmeier SA, Ladd-Effio C, Hubbuch J. Alternative separation steps for monoclonal antibody purification: Combination of centrifugal partitioning chromatography and precipitation. J. Chromatogr. A. 2013; 1319:

118–126.

5. Oelmeier SA, Ladd-Effio C, Hubbuch J. High throughput screening based selection of phases for aqueous two-phase system-centrifugal partitioning chromatography of monoclonal antibodies. J. Chromatogr. A. 2012; 1252: 104–114.

6. Gronemeyer P, Ditz R, Strube J. DoE based integration approach of upstream and downstream processing regarding HCP and ATPE as harvest operation. Biochem. Eng. J. 2016; 113: 158–166.

**Table 1 (abstract P-544).** ATPS composition

ATPS	Feed [w%]	Polymer [w%]		Salt [w%]		pH [-]	Displacement agent [w%]
		PEG 400	PEG1450	Phos-phate	Citrate		NaCl
1 [3]	44.5	15.5	-	16	-	6	-
2 [4]	26.4	19.6	-	-	18.9	6	-
3 [5]	40.5	-	6	15	-	6	10
4 [6]	27.2	6.8	-	26.12	-	7.3	0.7
Opt	36	19	-	16.4	-	8	4

**Table 2 (abstract P-544).** mAb yield and impurity removal in the LP of the different ATPS

ATPS	mAb yield [%]	DNA removal [%]	HCP removal [%]
1	93 ± 1	17 ± 3	-3.9 ± 1
2	87 ± 3	85 ± 1	21 ± 1
3	32 ± 4	97 ± 1	23 ± 2
4	33 ± 3	66 ± 1	-64 ± 4
Opt	92 ± 3	85 ± 2	52 ± 5

**Fig. 1 (abstract P-544).**
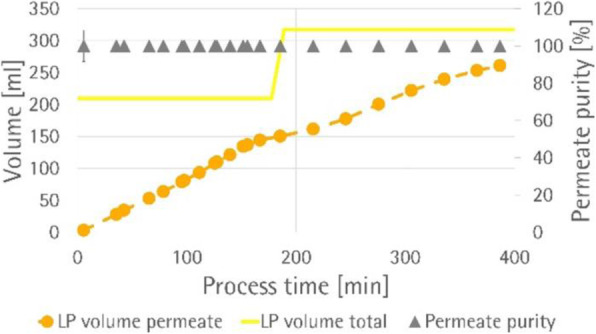
Volume of permeated LP and the permeate purity for the phase separation of the optimized ATPS. 83 ± 3 % LP recovery, Completely cell free permeate, 78 ± 3 % total mAb yield, 92 ± 3 % DNA removal, 43 ± 7 % HCP removal

### P-545 Impact of cell culture media additives on IgG glycosylation produced in CHO cells

#### Janike Ehret^1^, Martina Zimmermann^1,2^, Aline Zimmer^1^

##### ^1^Merck Life Sciences, Upstream R&D, Frankfurter Strasse 250, 64293 Darmstadt, Germany; ^2^Institute for Organic Chemistry and Biochemistry, Technische Universität Darmstadt, Alarich‑Weiss‑Strasse 4, 64287 Darmstadt, Germany

###### **Correspondence:** Janike Ehret (janike.ehret@merckgroup.com)

**Background**

Therapeutic glycoproteins are commonly produced in Chinese hamster ovary (CHO) cells and glycosylation represents one of the major critical quality attributes due to its influence on the safety, efficacy and half-life of the protein [1,2]. The five main glycosylation species can be classified into high mannose species, species with terminal N-acetylglucosamine, fucosylated species, galactosylated species and sialylated species. A variety of compounds were applied in CHO cell culture experiments to study their impact on the glycosylation profile of recombinant IgG.

**Materials and methods**

Fed-batch experiments were performed in spin tubes or the ambr®15 controlled bioreactor system with a CHO K1 and a CHO DG44 cell line expressing a recombinant IgG1 (mAb1 and mAb2 respectively). Compounds to modulate the glycosylation profile were added into the cell culture feed. The glycosylation profile as well as important cell culture parameters such as viable cell density, viability and titer were assessed during the fed-batch experiments.

**Results**

Out of the multitude of compounds and/or concentrations and/or different compound mixtures which were tested to modulate mannosylation (18), fucosylation (10), galactosylation (7) and sialylation (22) [3], the three conditions which showed the highest effect on each of the glycan species are presented (Figure 1). High mannose species were most successfully increased through 15 μM kifunensine supplementation (+85.8 %). A reduction of 76.1% fucosylation was achieved through 800 μM 2-F-peracetyl fucose. Galactosylated species were increased 40.9% by addition of 120 mM galactose in combination with 48 μM manganese and 24 mM uridine (UMG). Sialylation was enhanced by 6.9% through addition of 30 μM dexamethasone.

**Conclusion**

These cell culture media supplements represent a precious tool for the modulation of protein glycosylation and therefore for the production of recombinant proteins.

**References**

1. Hossler P, Khatta SF, Li ZJ. Optimal and consistent protein glycosylation in mammalian cell culture. Glycobiology. 2009; 19(9):936-949.

2. Jefferis R. Glycosylation of recombinant antibody therapeutics. Biotechnol Prog. 2005; 21(1):11-16.

3. Ehret J, Zimmermann M, Eichhorn T, Zimmer A. Impact of cell culture media additives on IgG glycosylation produced in Chinese hamster ovary cells. Biotechnol Bioeng. 2019; 116:816-830.

**Fig. 1 (abstract P-545).**
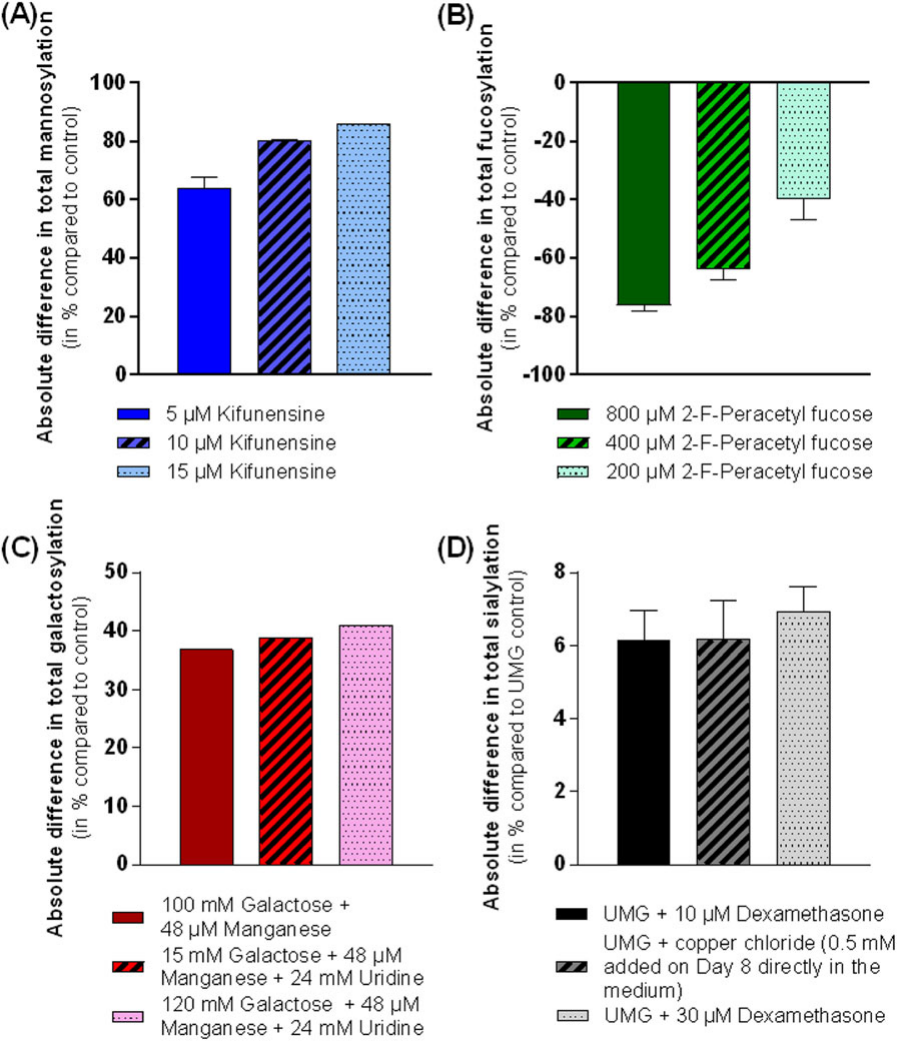
Absolute change in (A) mAb1 mannosylation, (B) mAb1 fucosylation, (C) mAb1 galactosylation and (D) mAb2 sialylation as compared to the respective control

### P-554 A versatile toolbox for rapid development of intensified CHO processes

#### Dirk Mueller^1^, Nico Erb^1^, Michael Grauf^1^, Lukas Klein^1^, Fabian Vogt^1^, Gernot Stipek^1,2^, Marisa Bertram^1^, and Gerben Zijlstra^3^

##### ^1^Sartorius Stedim Cellca GmbH, Laupheim, D‑88471, Germany; ^2^IMC University of Applied Sciences Krems, Krems, A-3500, Austria; ^3^Sartorius Stedim Biotech, Goettingen, D-37079, Germany

###### **Correspondence:** Dirk Mueller (dirk.mueller2@sartorius-stedim.com)

**Background**

Providing patients with affordable biopharmaceuticals requires next-generation processes with improved economics. Intensified processes enable plants with lower cost of goods, reduced footprints and increased flexibility, rendering compact bioreactors suitable for commercial production. CHO clones from traditional cell line development differ in responsiveness to process intensification, so efficient approaches for developing tailored processes are needed.

We have implemented a toolbox for rapid development of such USP processes. It comprises parallelized scale-down models for clone selection and media screening as well as different types of intensified formats. We demonstrate the application of this platform to different CHO clones.

**Materials and methods**

Preliminary clone assessment was performed in TubeSpin^®^ reactors for different CHO clones originally developed for fed batch. An ambr15®-based perfusion mimic served to optimize media compositions and to evaluate clones under controlled conditions. Briefly, cells were cultivated in the ambr 15 and bled as needed. Subsequently, reaction vessels were successively centrifuged in sets of 12 using specialized centrifuge inserts and then placed back into the ambr while cultivations in the other vessels continued undisturbed. Supernatant from the centrifuged vessels was syphoned off and replenished with fresh media using the liquid handler. Perfusion media consisted of an optimized blend of fed batch media and feeds [1].

Upscale was performed in rocking motion-based reactors for (N-1) perfusion and full perfusion. A high-inoculation fed batch process was developed in the ambr250 by evaluating varied inoculation densities and feeding profiles before transfer to the 5L scale.

**Results**

Most fed batch clones showed significant productivity improvements in intensified process formats. For some, limited improvements or even reduced productivity were seen, underscoring why scale-down models are key for identifying intensification-ready clones already in cell line development.

Cumulative titer increases >5-fold and 2.5‑fold relative to the standard fed batch were achieved for perfusion and high‑inoculation fed batch, respectively, without optimization (Figure 1a). This corresponded volumetric productivity increases of >4-fold and nearly 3‑fold, respectively.

Scale-up to larger volumes demonstrated that volumetric productivities determined in the ambr 15 perfusion mimic were predictive of the true perfusion setup (Figure 1b).

In a related application, a high-inoculation fed batch process was optimized in the ambr 250 before scaling up to 5L reactors. These demonstrated a 53% titer increase and time savings of four days compared to the conventional 14d fed batch.

We further evaluated the feasibility of using high cell density cell banks in cryobags as process intermediates. Starting from an N-1 perfusion culture with 100 mio cells/mL, cell banks were frozen and stored at -80°C for 2 months. Upon thawing, they served to directly inoculate a fed batch process and provided robust growth and productivity.

Finally, applying the ambr 15 perfusion mimic to optimizing product quality patterns for a biosimilar using a DoE-based approach for process parameters and media variants resulted in a significant improved match to the originator quality profile (Table 1).

**Conclusions**

The above examples illustrate how the toolbox supports cell line development for a range of intensified process formats in order to unleash unutilized performance reserves of CHO production clones. The presented approach is generic in nature and may also be extended to other cell lines.

**Acknowledgements**

Support by Sartorius Cell Culture groups at Göttingen and Bohemia for part of this work is gratefully acknowledged.

**Reference**

1. Janoschek S, Schulze M, Zijlstra G, Greller G, Matuszczyk J (2019) Biotechnol Prog 35(2):e2757.

**Fig. 1 (abstract P-554).**
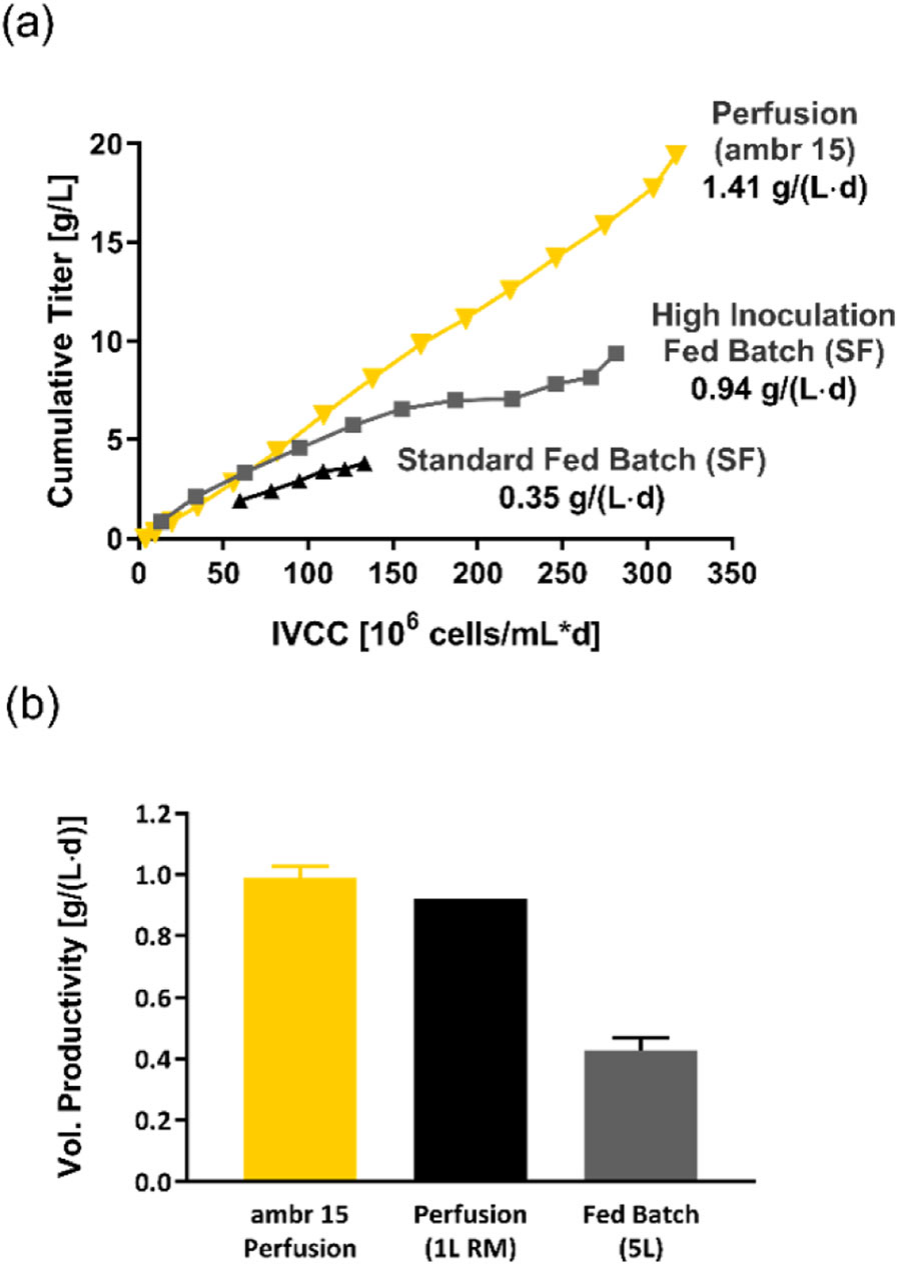
(a) Comparison of cumulative titers and volumetric productivity (Clone A), (b) Performance in the ambr 15 perfusion mimic correlates with the true perfusion process (Clone B)

**Table 1 (abstract P-554).** Improvement of product quality match to the innovator for a biosimilar achieved using the ambr 15 perfusion mimic

Performance Trait	Improvement
Charge Variants	- 44% Total Deviation
N-Glycans	- 17% total Deviation
Volumetric Productivity	+ 11 %

### P-561 Perfusion media development for scalable processes

#### Patrick Mayrhofer^1^, Andreas Castan^2^, Renate Kunert^1^

##### ^1^Department of Biotechnology, University of Natural Resources and Life Sciences (BOKU), Vienna, 1190, Austria; ^2^GE Healthcare Bio-Sciences AB, Uppsala, 751 84, Sweden

###### **Correspondence:** Patrick Mayrhofer (patrick.mayrhofer@boku.ac.at)

**Background**

Cell culture perfusion processes are considered optimal for a truly integrated continuous biomanufacturing pipeline [1]. The nutrient-rich but balanced media is designed to support very low cell-specific perfusion rates (CSPR) to minimize media consumption while maximizing viable cell days and productivities. Optimized processes at low CSPR drastically reduce equipment costs, lab space, and product dilution. Finally, operating at very low CSPR will allow for mammalian cell bioprocesses to run as true chemostat cultures in the future. In this study, we demonstrate a general workflow to develop high-performing perfusion media using small-scale models and transferring the process to a 50 L scale at CSPR of 20 pL/cell/day.

**Materials and methods**

Cell line, basal media and feed supplements:

A recombinant CHO-K1 cell line producing an IgG1 antibody adapted to ActiPro or CDM4NS0 basal media was used in this study. Eight feed supplements (‘Cell Boosts’) were used to develop novel perfusion media.

Semi-continuous small-scale models:

‘Pseudo-perfusion’ experiments were initiated by seeding 10 × 10^6^ c/mL in 10 mL of fresh media. Spent medium was replaced daily by centrifugation and a complete media exchange.

Bioreactor verification runs:

Bioreactor perfusion runs were initiated by seeding 1 × 10^6^ c/mL in fresh basal media and perfusion was initiated after a two- to four-days batch phase at pH 6.8, 37°C and 30% dissolved oxygen.

**Results**

A generally applicable perfusion medium development workflow was applied to two different basal media (Figure 1A). In a first screening round, beneficial effects of Cell Boost supplements 1, 3, 7a, and 7b were identified using a DoE approach in spiked batch cultures. The pre-selected supplements were subsequently applied to a second DoE (Figure 1B) using 10 mL shaking cultures in a semi-continuous perfusion mode by daily media exchange. This allowed to fine-tune the ratio of pre-selected feed supplements. High VCDs of more than 50 × 10^6^ cells/mL in a quasi steady-state were reached. Spiking the basal medium with feed supplements improved viabilities and daily titers up to 1 g/L. Two novel perfusion media, developed within this project, based on basal CDM4NS0 or ActiPro, supplemented with Cell Boost 1 and Cell Boost 3, were finally applied to different bioreactor perfusion verification runs. Using a continuous volumetric perfusion rate, the minimum CSPR of 10 pL/cell/day was determined to generate the highest VCD of more than 200 × 10^6^ cells/mL. A similar high VCD was reached using a constant CSPR of 15 to 30 pL/cell/day to reduce medium consumption (Figure 1C). The novel perfusion media were also applied to bioreactor production runs at a constant VCD of 50 × 10^6^ cells/mL at a 500 mL or 40 L scale. An increase of galactosylated glycan species was observed over process time, and a good correlation of various parameters compared to the small-scale model was identified. Major differences to the small-scale model were only found for the glutamate/glutamine/NH4+ behavior, which might be responsible for the discrepancy of terminal galactosylation profiles.

**Conclusions**

A DoE-based workflow was developed to leverage established feed supplements for definition of novel, high-performing perfusion media within two months. The key technique in this workflow was to use small-scale models in semi-continuous perfusion mode to screen many different conditions by a single operator. The generated data set was then used for multivariate (regression) analyses to define the optimal feed supplement and –concentrations to finally apply the novel media to 0.5L or 50L bioreactor verification runs at very low CSPR (10-20 pL/cell/day). Future projects will elucidate differences in growth-, productivity- and product quality between small-scale and bioreactor cultures

**Acknowledgements**

We gratefully thank Florian Wiederstein, Lukas Damjanovic and Willibald Steinfellner for technical assistance.

**Reference**

1. Bielser, J.-M., Wolf, M., Souquet, J., Broly, H., & Morbidelli, M. (2018). Perfusion mammalian cell culture for recombinant protein manufacturing – A critical review. Biotechnology Advances, 36(4), 1328–1340. https://doi.org/10.1016/j.biotechadv.2018.04.011.

**Fig. 1 (abstract P-561).**
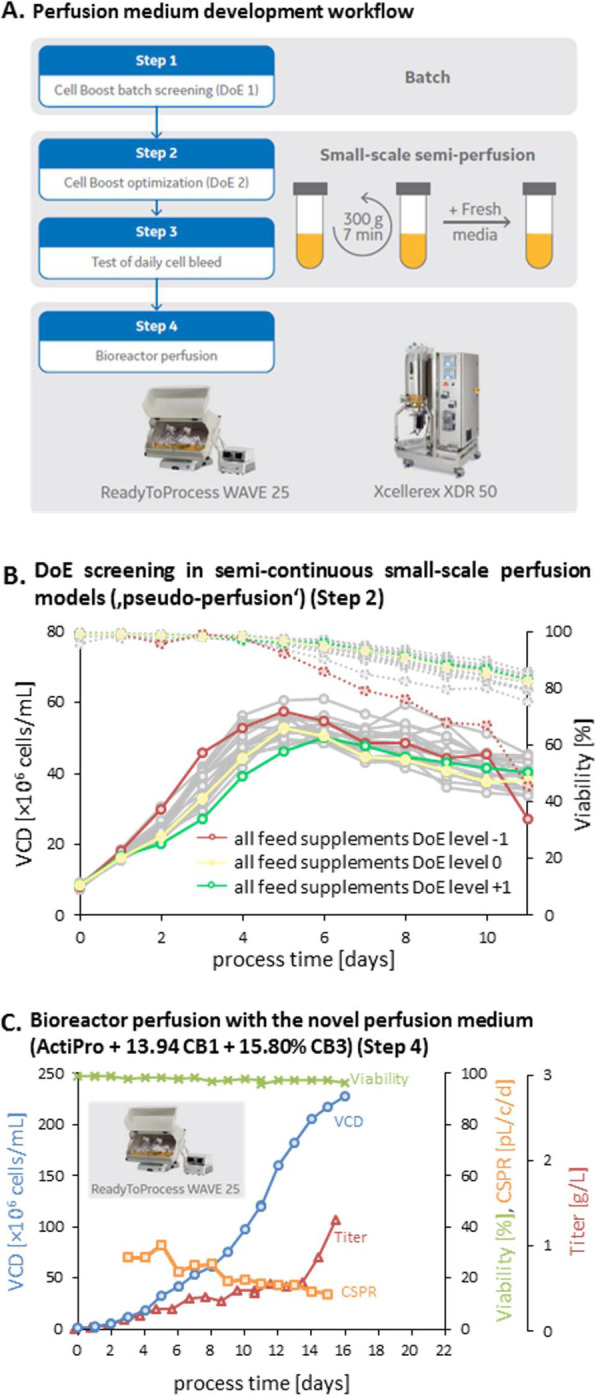
Fast workflow for perfusion media development. After initial definition of optimal basal media, eight different feed supplements were screened, optimized, and tested in four consecutive steps (A.). In step 2, a DoE with small-scale semi-continuous perfusion models was applied to optimize the pre-selected feed supplements and to establish valid regression models (B.). The novel media was applied to a perfusion bioreactor (C.)

### P-565 Media supplement induced signaling in CHO cells via triple SILAC-MS

#### Louise Schelletter, Thomas Noll, Raimund Hoffrogge

##### Cell Culture Technology, Bielefeld University, Bielefeld, 33615, Germany

###### **Correspondence:** Louise Schelletter (l.brachtvogel@uni-bielefeld.de)

**Background**

Process intensification of Chinese hamster ovary (CHO) cell culture often involves supplementation with (small) molecules to improve process performance. However, screening for beneficial agents remains time and cost intensive. Identification of underlying cellular mechanisms e.g. by signaling studies using quantitative phosphoproteomics [1,2,3] would allow targeted applications.

In a triple “stable isotope labeling by amino acids in cell culture- mass spectrometry” (SILAC-MS) approach of monoclonal antibody (mAb)-producing CHO cells, we aimed to identify cellular targets and to elucidate synergistic effects of cell growth enhancing agents on a molecular level, which is here shown for insulin-like growth factor (IGF) and glutamine.

**Materials and methods**

CHO cells were cultivated in chemically-defined media CDM (Xell AG). For SILAC-MS experiments media with either light, medium or heavy lysine and arginine were utilized, and 8 mM L-glutamine was supplemented if indicated.

IGF was added at day 3 and few minutes later cell aliquots were harvested and pooled for SILAC-MS. Proteins were purified, reduced, alkylated and digested with trypsin. An enrichment of phosphopeptides was performed via TiO_2_-beads. Bottom-up proteomic analysis was carried out by nLC-ESI Orbitrap MS (Q Exactive Plus, Thermo) with subsequent data analysis based on ProteomeDiscoverer (Thermo Fisher Scientific) and Fusion software [4].

**Results**

Synergistic effects of IGF- and L-glutamine-supplementation was evaluated via label-free cultivations of glutamine synthetase (GS)-system derived, mAb-producing CHO cells in CDM. IGF supplementation led to a sustained high viability and an increased maximum VCD (Fig. 1 A, left). Early sampling revealed rapid changes in signaling cascades directly linked to cellular metabolic processes via AKT and ERK1/2, whereas according protein level stayed constant (Fig. 1 B, left). Maximum phosphorylation of AKT and ERK1/2 was detected in Western blots 5 minutes past IGF induction (Fig. 1 C, left).

Glutamine supplementation of CDM for GS-system derived CHO cells resulted in an increased maximum VCD. Surprisingly, the combination of IGF and glutamine yielded in reduced maximum VCD (compared to glutamine or IGF supplemented) (Fig. 1 A, right). Again, early sampling proved rapid signaling events (Fig. 1 C, right) without changes in protein expression (Fig. 1 B, right). However, in cultures without glutamine the intensity of targeted phosphorylation-sites is higher (investigated by Western blot).

In an additional triple SILAC experiment effects of IGF +/- glutamine were relatively quantified on protein and phosphopeptide-level. Within short-gradient MS measurements for each of three biological replicates about 1800 proteins and 2000 phosphopeptides were quantified. With a focus on quantification of extracellular signal regulated kinase (ERK) 1 and 2, we detected a minor decline in ERK2 expression upon IGF treatment (Fig. 1 D, left).

The ratios for the unphosphorylated ERK2 peptide variant for +IGF/control and +IGF+Gln/control decrease over time, which correlates to an increase in ratios of the according phosphorylated peptides (Fig. 1 D, right).

Hence, Western blot and MS data correlate well. However, due to the low abundance of the phosphopeptide in control culture sometimes uncertainties in MS quantification remain (* in Fig. 1 D, right).

For visualization of complex data including dynamic protein phosphorylation patterns, we established and successfully applied a semi-automatic expression data mapping in Fusion software [1,3] (Fig. 1 E).

**Conclusions**

IGF supplementation in glutamine-containing media for cultivation of GS-based recombinant CHO cells led to decreased maximum VCD compared to control without IGF. In addition, IGF-induced upregulation of specific phosphopeptides is reduced by glutamine, which was validated by the orthogonal approaches, Western blots and SILAC-MS.

Thus, cultivation with glutamine inhibits the growth promoting effect of IGF. This seems to correlate to less intense signaling via AKT and ERK in cultivations without GS selection pressure.

Further characterization of producer CHO clones by quantitative MS will allow a rational application of growth enhancing agents.

**Acknowledgements**

We would like to thank the AIBN (Brisbane) for providing the CHO lines and Larissa Leßmann for excellent support with Western blotting.

**References**

1. Schelletter L, Albaum SP, Walter S, Noll T, Hoffrogge R. Clonal variations in CHO IGF signaling investigated by SILAC-based phosphoproteomics and LFQ-MS. Appl Microbiol Biotechnol. 2019.

2. Müller B, Heinrich C, Jabs W, Kaspar-Schönefeld S, Schmidt A, Rodrigues de Carvalho N, Albaum SP, Baessmann C, Noll T, Hoffrogge R. Label-free protein quantification of sodium butyrate treated CHO cells by ESI-UHR-TOF-MS. J Biotechnol. 2017; 257:87-98.

3. Brachtvogel L, Walter S, Noll T, Hoffrogge R. BMC Proc. MS-SILAC approach for phosphoproteomics of IGF signaling in producer CHO cells. 2018; 12(Suppl 1):3.

4. Brink BG, Seidel A, Kleinbölting N, Nattkemper TW, Albaum SP. Omics Fusion - A platform for integrative analysis of omics data. J Integr Bioinform. 2016; 13:296.

**Fig. 1 (abstract P-565).**
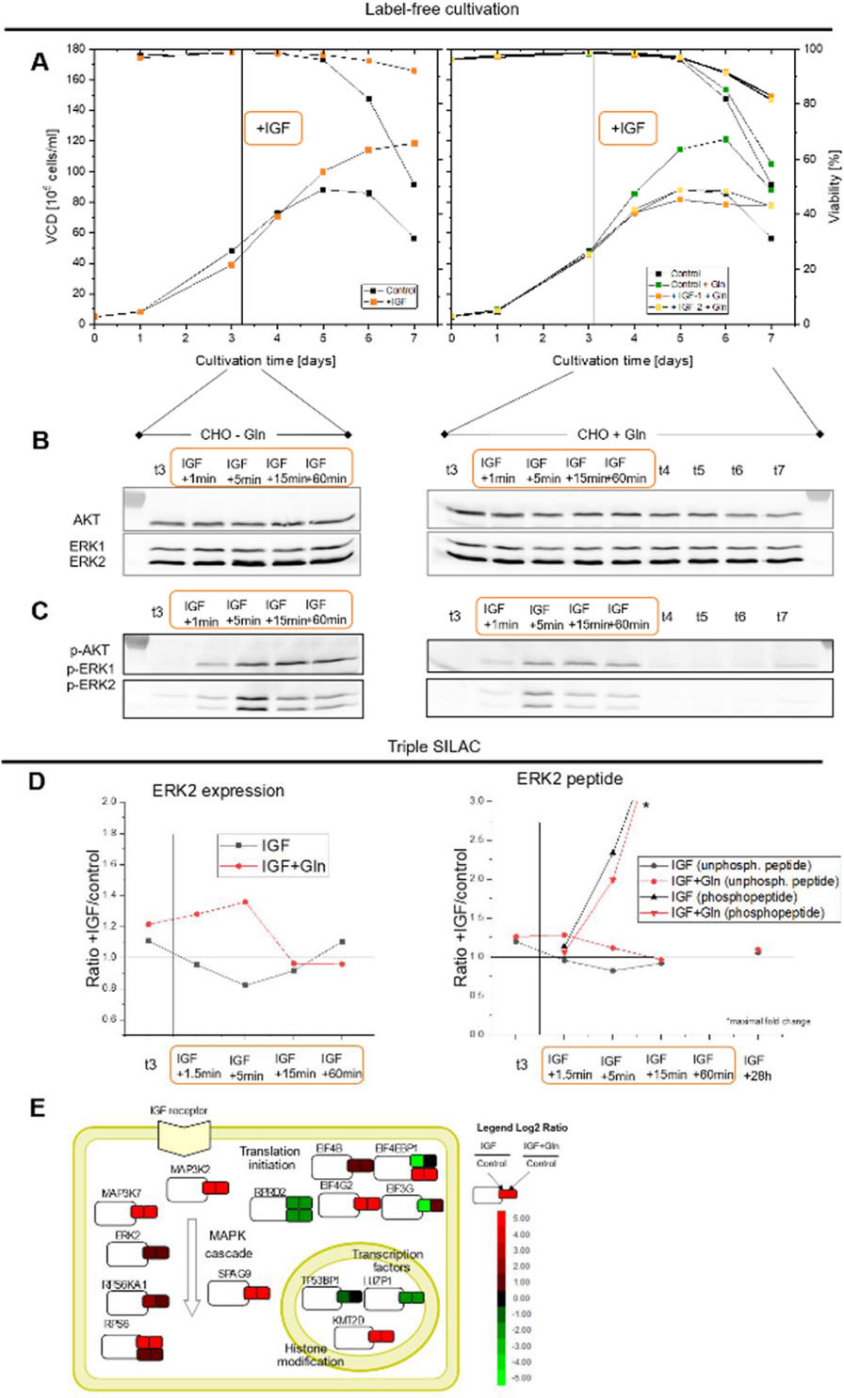
A) Cultivation of mAb-producer CHO cells w/o and with IGF supplementation at day 3 without glutamine (left) and with glutamine (right). B) Western blot to present ERK1/2 and AKT protein level over time past IGF induction in cultures with and w/o glutamine. C) Relative quantification of changes in phosphorylation of AKT and ERK1/2 utilizing phospho-specific antibodies in western blots. Signals were normalized to total loaded protein amount via Coomassie-stained membrane. D) Triple SILAC-MS data of replicate 1 evaluated by Proteome Discoverer software filtered for ERK quantification as time course past IGF induction with and without glutamine. ERK2 protein level (left) and ERK2 peptide expression data as unphosphorylated peptide and double phosphorylated variant (right). E) Significantly regulated phosphopeptides for one selected time point (5 min past IGF induction) with or without glutamine

### P-568 CHO seed culture intensification and its cellular effects on N-stage

#### Markus Schuze, Jens Matuszczyk, Gerhard Greller

##### Sartorius Stedim Biotech, Göttingen, Germany, 37079

###### **Correspondence:** Markus Schuze (markus.schulze@sartorius-stedim.com)

**Background**

Reducing the plant size and footprint of today’s manufacturing processes is one key aspect of sustainable and economic production of biopharmaceuticals. One approach during mammalian cell cultivation covers the intensification of the seed train by applying perfusion as N-1. Currently, latest research mainly assesses the impact of perfusion on cell cultivation by examining product quality and capacity, but ignores cellular responses initiated by process intensification strategies. In this study, both features are considered in fed-batch (FB) processes based on an intensified N-1 seed train using perfusion.

**Material and methods**

A perfusion protocol was developed based on a standard CHO-DG44-Fed-Batch platform producing a monoclonal antibody (mAb; Sartorius Stedim Cellca) [3] and transferred into a wave-mixed bioreactor (BIOSTAT® RM, Sartorius Stedim Biotech). This process used a minimum cell specific perfusion rate (CSPR) of 50 pL∙c^-1^∙d^-1^ at 36.8 °C, pH 6.95 and DO 60%. Agitation (rocks per minute) at 10° rocking angle was aligned to maintain the latter one at HCC. The N-1 perfusion was set to reach 100E6 c∙mL^-1^ within 7 days.

The corresponding N-stage fed-batch process was carried out in single-use, small scale automated bioreactors (ambr15®, Sartorius Stedim Biotech) at 36.8 °C, pH 7.1 and DO 60%. Feeding (Feed A and B, 1:10) was started at day 3 respectively day 5 for additional glucose. Based on the origin of the N-1 cell inoculum, different process scenarios were conducted in triplicates (Table 1).

**Results**

The applied N-1 perfusion process was started at 2.5E6 c∙mL^-1^ and reached 120E6 c∙mL^-1^ within 7 days. Maintained high viabilities (>95%) throughout the whole cultivation indicated the potential use as appropriate N-1 bioreactor.

Based on the N-1 perfusion process two N-stage fed-batch processes (Perf d5 and d7) were started and compared to a process inoculated from a conventional seed train (CTRL). After cells were removed and transferred from the N-1 perfusion bioreactor to the ambr15®, automated inoculation at 0.3E6 c∙mL^-1^ succeeded even for small volume HCC inocula, i. e. 65 μL (Perf d5) and 32.5 μL (Perf d7) using the system’s robotic arm. Each of the three approaches’ cell growth was perfectly in line with the GB expectations and reached peak cell concentrations of 22.2 ± 0.7E6 c∙mL^-1^ between day 7 and 8. After 12 days, final VCCs were at about 13E6 c∙mL^-1^ (Fig. 1A)). Regarding cell viability, generally all approaches were found to be close to the golden batch (GB) as well. Partial data points for Perf d7 were out of the trajectory at the end of the process, but were still above common harvest criteria of 70% (Fig. 1B)) [4].

Product titers were found to be comparable with the GB (3.5 ± 0.4 g/L) for all three approaches (Fig. 1C)), while identical glycosylation patterns (Fig. 1D)) at the N-stage can still be expected and achieved by applying seed train intensification.

**Conclusions**

To summarize, BIOSTAT® RM bioreactors can be used for seed train intensification applying perfusion in order to reach HCC, while simultaneously consistent product titers and qualities can be expected at subsequent N-stage processes. Simultaneously, ambr15® bioreactors allow to reproduce and depict results of large scale processes, thus being optimally suited for multi-parallel parameter screening and optimization in a high throughput manner. Combining both processes and systems shown above allows for identification and design of an intensified CHO process that can then be used as starting point for scale up.

**Acknowledgements**

The authors thank the complete BioProcessing team of Sartorius Stedim Biotech GmbH, Göttingen.

**References**

1. BioPhorum Operations Group, Biomanufacturing Technology Roadmap.

2. Matuszczyk, J, Schulze, M., Janoschek, S., Zijlstra, G., Greller, G. Poster presentation at Bioprocessing Summit Europe (2018).

3. Janoschek S, Schulze M, Zijlstra G, Greller G, Matuszczyk J. Biotechnol Prog. 2019; 35: epub.

4. Fan Y et al. Biotechnol Bioeng. 2015; 112:2172-84.

**Table 1 (abstract P-568).** Inoculation scenarios for intensified N-1 processes

Approach	Source	VCC [E6 c∙mL^**-1**^]
CTRL	Regular seed	3
Perf d5	Perfusion day 5	60
Perf d7	Perfusion day 7	120

**Fig. 1 (abstract P-568).**
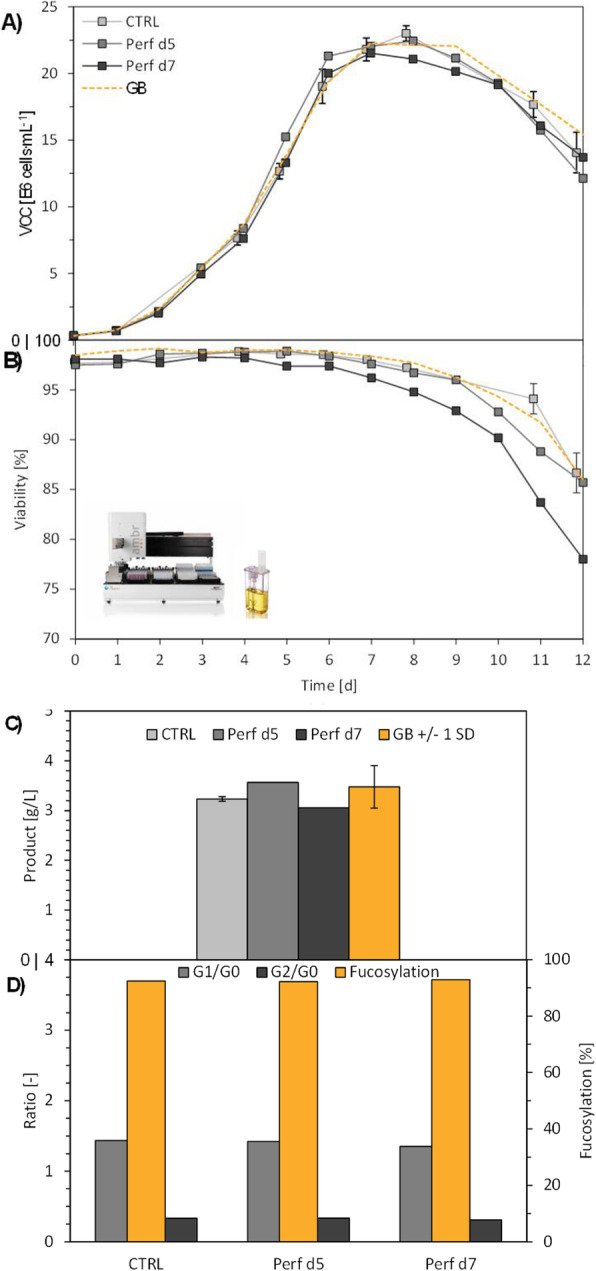
Comparison of A) VCCs, B) viabilities, C) product titer and D) quality, i.e. glycosylation patterns at day 12, of fed-batch processes inoculated either from conventional or intensified seed trains

### P-582 Development of chemically defined medium for Vero cells

#### Gerco van Eikenhorst^1^, Bella Monica^1^, Roni Hazan Brill^2^, Emilie Rodrigues^1^, Yvonne E. Thomassen^1^

##### ^1^Intravacc, P.O. Box 450, 3720 BA Bilthoven, The Netherlands; ^2^Biological Industries Israel Beit Haemek Ltd., Beit Haemek, Israel 25115

###### **Correspondence:** Gerco van Eikenhorst (gerco.van.eikenhorst@intravacc.nl)

**Background**

Intravacc develops processes for viral vaccines. A Vero cell platform is available and can support the propagation of many types of viruses. To further optimize cell and virus culture it is desirable to know the nutrients that are depleted at different cultivation phases. Current available media, including the animal component free media, contain undefined ingredients which makes this analysis more challenging. In collaboration with Biological Industries the development of a chemically defined (BI-CD) medium for the growth of Vero cells and subsequent virus production was pursued.

**Materials and methods**

Vero cells were cultured in 6-well plates and T-flasks for screening several media formulations on growth rate and maximum cell density. VP-SFM (Thermo Fisher Scientific), a commercially available animal component free (ACF) culture medium was used as control. Several iterations of media optimization were carried out. Next, media were evaluated to support Vero cell growth on microcarriers (Cytodex 1, GE Healthcare) in spinner flasks and subsequently in bioreactors (Sartorius). Finally, the ability to support virus propagation was assessed.

**Results**

After several iterations of media optimization in static cultures, six media formulations were tested for their ability to support Vero cell growth on microcarriers in spinner flasks. Maximum cell densities up to 1·10^6^ cells·mL^-1^ were obtained. The three media that supported growth best were further optimized prior to tests in bioreactor cultures. Two of the three improved media formulations yielded a maximum cell concentration of 1.4·10^6^ cells·mL^-1^ in bioreactor.

These two media formulations (BI-CD-J and BI-CD-K) were selected to evaluate the capability to support virus propagation. Both media were capable to propagate all tested viruses: measles (Edmonston), enterovirus 71 (EV A71-C4) and poliovirus Sabin type 1 (sPV T1) (Table 1).

Chemically defined BI-CD-J showed most promising results in bioreactor cultures (Figure 1). After 5 days of cell culture, microcarriers were equally and fully confluent and a maximum cell concentration (C_max_) of 1.5 ± 0.1·10^6^ cells·mL^-1^ (n=3) with a specific growth rate (μ) of 0.026h^-1^ was found. These results are comparable to those for Vero cell growth in VP-SFM (C_max_=1.4·10^6^ cells·mL^-1^; μ=0.025h^-1^).

The BI-CD-J formulation also showed good results as a propagation medium for different viruses (Table 1) in T-flasks.

Virus propagation on Vero cells grown on BI-CD-J medium was assessed in bioreactors (n=3). Infection with EV A71-C4 was performed after three days of cell culture when a cell concentration of 0.7-1.0·10^6^ cells·mL^-1^ was obtained. Virus culture was complete after 74 hours post infection. The virus yield was 6.8±0.1 log_10_CCID_50_·mL^-1^, which is comparable to the virus yield in VP-SFM (6.7 log_10_CCID_50_·mL^-1^).

**Conclusions**

BI-CD-J is a new developed, fully chemically defined and animal component free culture medium that supports Vero cell growth in static and in bioreactor cultures and is able to support propagation of different types of viruses.

In addition, yields for Vero cell growth and infectious virus particles are comparable to those found for VP-SFM. The media is currently available at Biological Industries and is named NutriVero™ Flex 10.

**Table 1 (abstract P-582).** Virus propagation on different media in T-flasks. Virus was quantified using the CCID_50_ method

Medium	Virus titer (log_**10**_ CCID_**50**_/mL)		
	Measles	EV A71-C4	sPV T1
VP-SFM	<LOQ	5.9	7.4
BI-CD-J	4.6	7.6	6.9
BI-CD-K	4.7	6.7	6.5

LOQ limit of quantitation

**Fig. 1 (abstract P-582).**
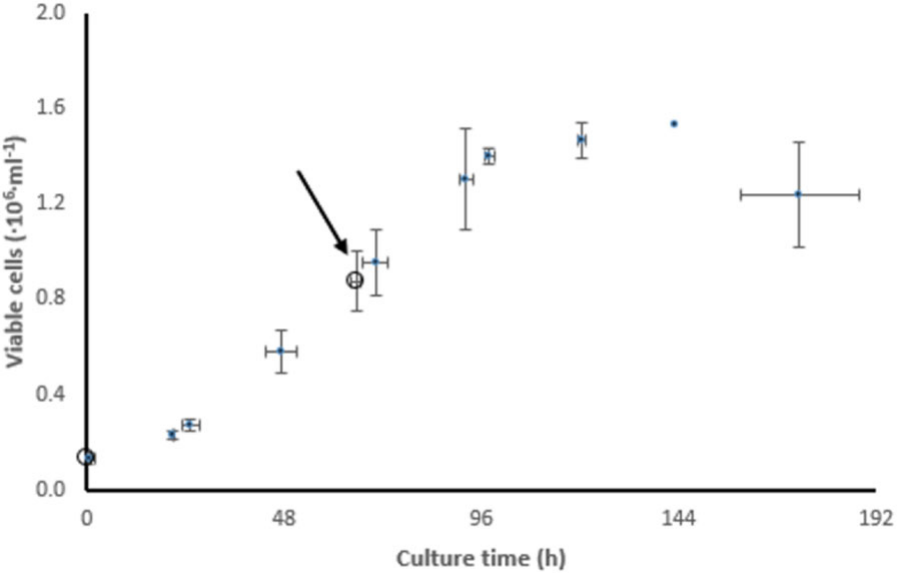
Vero cell growth curve in chemically defined BI-CD-J medium in bioreactors (closed dots; n=3). Open circles represent Vero cells cultured in bioreactors (n=3) with BI-CD-J media which were infected with EV-A71 C4 at 3 days (arrow)

### P-591 Expression of the “difficult-to-express” protein CD19

#### Elisabeth Lobner^1^, Anna Wachernig^1^, Venugopal Gudipati^2^, Patrick Mayrhofer^1^, Willibald Steinfellner^1^, Johannes B. Huppa^2^, Renate Kunert^1^

##### ^1^Department of Biotechnology, BOKU - University of Natural Resources and Life Sciences, 1190 Vienna, Austria; ^2^Institute for Hygiene and Applied Immunology, Center for Pathophysiology, Infectiology and Immunology, Medical University of Vienna, 1090 Vienna, Austria

###### **Correspondence:** Elisabeth Lobner (renate.kunert@boku.ac.at)

**Background**

CD19 (Cluster of Differentiation 19) is a transmembrane protein, expressed exclusively on normal and malignant B cells, which makes it an attractive target for cancer immunotherapy. However, the extracellular domain of CD19 (CD19-ECD) is classified as a “difficult-to-express” protein, characterized by very low product titers and formation of disulfide-bonded oligomeric aggregates attributed to incorrect protein folding. In this study, we designed a novel fusion construct of CD19-ECD and exploited different strategies that resulted in the recombinant expression of the protein in high quality and sufficient amounts to conduct various experiments.

**Materials and methods**

The CD19-ECD was fused to the domain 2 of human serum albumin (CD19-AD2, Fig. 1). This construct was integrated into the *Rosa26* bacterial artificial chromosome (BAC) vector backbone for generation of a recombinant CHO-K1 production cell line [1]. To further enhance protein expression different chemical chaperons were tested regarding their influence on product generation. Purified CD19-AD2 was characterized in regards to correct protein folding as well as biological activity.

**Results**

Addition of valproic acid (VPA) has proven to be highly beneficial in terms of product titers. The resultant recombinantly expressed CD19-AD2 is stable in the monomeric form as shown by non-reduced SDS-PAGE and SEC-MALS measurements. Moreover, flow cytometric analysis revealed specific binding of CD19-AD2 to CAR-T cells. Finally, we demonstrated biological activity of our fusion construct through activation of CAR expressing T cells upon recruitment of CARs and CD19-AD2 into immunological synapses. T cell activation was detected by total internal reflection fluorescence (TIRF) microscopy (Figure 1).

**Conclusions**

The data in this study describe the journey from the molecular design to the successful recombinant expression of the “difficult-to-express” protein CD19. The generated CD19-AD2 fusion protein is natively folded, present in monomeric form and highly stable. Additionally, functionality was verified by activation of CAR-T cells demonstrating full structural integrity and biological activity of CD19-AD2.

**Acknowledgements**

This work was sponsored by the platform for advanced cellular therapies (PACT).

**References**

1. Blaas, L., Musteanu, M., Eferl, R., Bauer, A., Casanova, E. Bacterial artificial chromosomes improve recombinant protein production in mammalian cells. BMC Biotechnol. 2009; 9:3

2. Axmann, M., Schütz, G.J., Huppa, J.B. Single Molecule Fluorescence Microscopy on Planar Supported Bilayers. J Vis Exp. 2015; 104: 53158

**Fig. 1 (abstract P-591).**
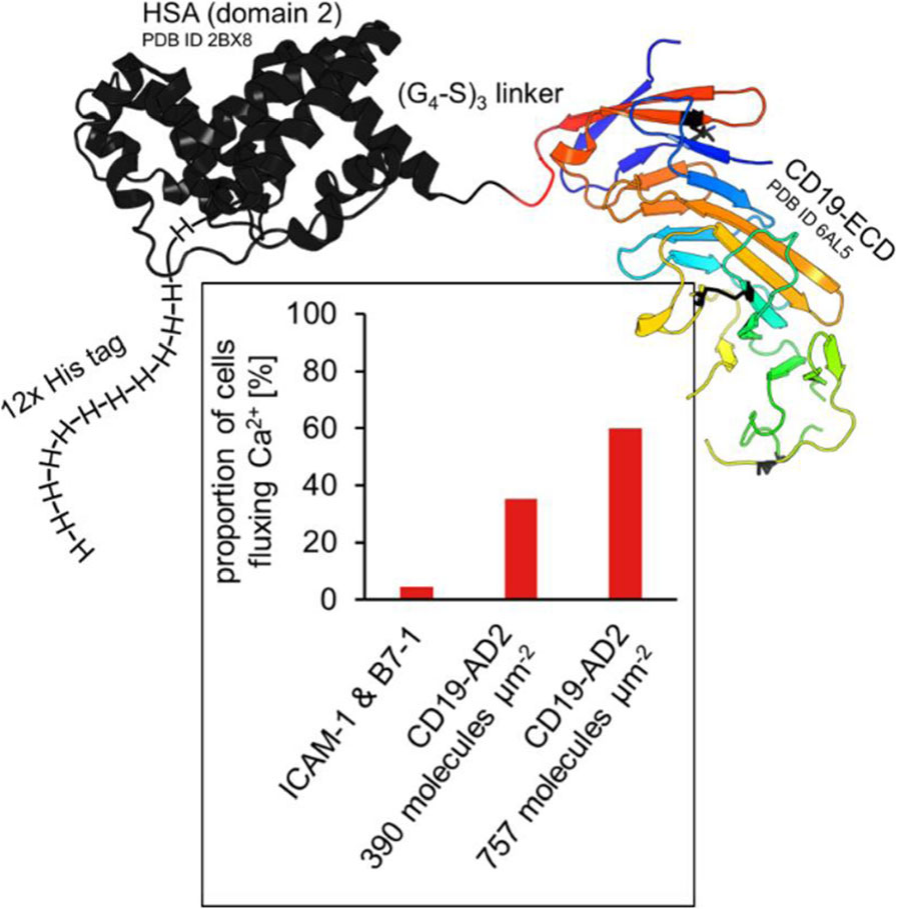
Schematic representation of the expressed CD19-AD2 fusion construct and evaluation of T cells fluxing Ca^2+^ for determination of the biological activity of CD19-AD2. Therefore, a planar glass-supported lipid bilayer (SLB) was functionalized with the adhesion molecule ICAM-1, the costimulatory molecule B7-1 and CD19-AD2 for recognition by CAR-T cells [2]. The proportion of Ca^2+^ signaling cells at two different CD19-AD2 densities on the SLB was measured. As negative control, cells were additionally confronted with antigen-free SLB presenting only ICAM-1 and B7-1

### P-592 Mitigate adventitious agent contamination risks in cell culture media

#### Leila Djemal^1^, Alexandre Gilet^2^, Soraya Alves Caetano^1^, Murielle Philippoz ^1^, Véronique Deparis^1^

##### ^1^Manufacturing Science and Technology; ^2^ Biotech Processes Sciences, Merck KGaA, Fenil-sur-Corsier,1809, Switzerland

###### **Correspondence:** Leila Djemal (leila.djemal@merckgroup.com)

**Background**

Some viral contamination events have been publicly reported in the biopharmaceutical industry. Upstream raw materials were often identified as the potential source of contamination. To mitigate this risk, different inactivation methods could be used such as high temperature short time - HTST, ultraviolet irradiation and gamma irradiation technologies, or viral removal technology like nanofiltration, applied on raw materials or on cell culture media. The aim of this study was to assess the impacts of HTST and UV-c treatment on cell culture media for two different processes (process A and process B). HTST treatment corresponds to the rapid heating of a solution to a targeted temperature and holding time to inactivate adventitious agents like viruses by the disintegration of the viral-capsid [1]. Ultraviolet irradiations are targeting DNA/RNA of viruses. Under these radiations cyclobutane pyrimidine dimers are formed within the double strand leading to defective genetic material. [2]

**Materials and methods**

A FT74-20-MkIII UHT/HTST system from Armfield was used to heat media, and a laboratory prototype was used for UV-c irradiation. Different conditions were tested: temperature and exposure time for HTST, irradiation time and flow for UV-c. For each condition, metabolism, cell growth, productivity and product quality profiles were evaluated in comparison with a non-treated media, using small scale cell culture systems. In parallel, media were also characterized to quantify the impacts of inactivation methods on some critical components.

**Results**

HTST: For process A, when the production medium was treated at 102°C for 10 seconds, no significant impact was observed on cell growth, metabolism, productivity, neither on critical quality attributes. (Figure 1). Other temperatures (98, 100 and 104°C) and holding times (8 and 12 seconds) were tested, and no significant impact on process performance was observed. However, some components in the media seemed to precipitate during heat treatment which led to a pressure increase during filtration of media. Those components will be identified in further analyses to maintain medium integrity and avoid filterability issues. For process B, when the production medium was heated, a negative impact was observed on cell growth and productivity. A loss of 28% of insulin was measured after heat treatment of the production medium. Trace metals (e.g. iron) present in the media were also decreased by heat treatment.

UV-C: For the highest dose of UV-c (lowest flow), no impact on trace metals or amino acids was detected, but negative impacts on vitamins, cell viability and productivity were measured. Vitamins were highly degraded due to their high absorbance at wavelength close to the chosen irradiation. The maximum dose applied for the absence of impact on cell culture was determined. Subsequently, it will be necessary to determine if chosen conditions allow to ensure viral inactivation.

**Conclusions**

For some processes, HTST treatment can be directly implemented, while for others it requires further development. In the case of processes A and B, application of HTST treatment requires to identify and remove heat labile components before treatment and adding them afterwards. UV-c irradiation was less suitable for media tested compared to HTST treatment. One reason is the strong impact that was observed on cell growth profile, and beyond media compatibility, the robustness of viral clearance, the scalability and the cost effectiveness are difficult to ensure for UV-c treatment. Indeed, this technology, is associated to a large set of parameters to control (e.g. absorbance, flow, viscosity, temperature, materials used) that make it challenging to monitor and control at both laboratory and manufacturing scale compared to HTST technology.

**References**

1. Boschetti N., Wyss K., Mischler A, et al. Stability of minute virus of mice against temperature and sodium hydroxide. Biologicals. Volume 31, no 3. 2003 p. 181-185.

2. Sinha R. P., et Häder, D.P. UV-induced DNA damage and repair: a review. Photochemical & Photobiological Sciences. Volume 1, no 4. 2002 p. 225-236.

**Fig. 1 (abstract P-592).**
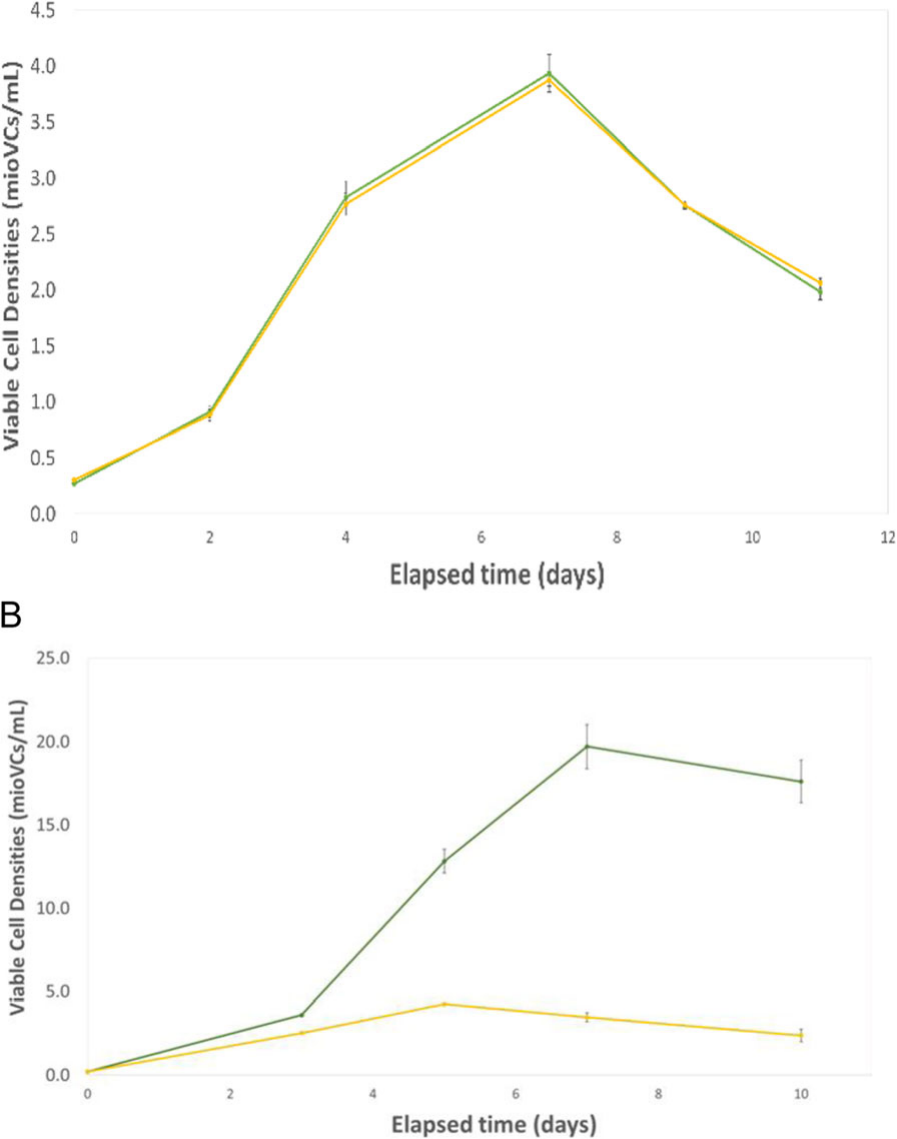
Impact of production media heat treatment (102°C; 10 seconds) on viable cell density (processes A and B). Green: untreated media; yellow: treated media

